# Pre‐season friendly match‐play as a double‐edged sword: Performance gains and inflammatory cost in semi‐professional soccer players (a natural experiment)

**DOI:** 10.1113/EP093797

**Published:** 2026-10-27

**Authors:** Dimitrios I. Bourdas, Eleftherios Mylonis, Natalia Kompodieta, Panteleimon Bakirtzoglou, Athanasios Souglis, Evangelos Bekris, Antonios K. Travlos, Apostolos Theos

**Affiliations:** ^1^ Section of Sport Medicine & Biology of Exercise School of Physical Education and Sports Science National and Kapodistrian University of Athens Daphni Greece; ^2^ School of Physical Education and Sports Science Aristotle University of Thessaloniki Serres Greece; ^3^ School of Physical Education & Sport Science National and Kapodistrian University of Athens Daphni Greece; ^4^ School of Physical Education and Sport Science Aristotle University of Thessaloniki University Campus Thessaloniki Greece; ^5^ Department of Sports Organization and Management Faculty of Human Movement and Quality of Life Sciences University of the Peloponnese Sparta Greece; ^6^ Section of Sports Medicine Department of Community Medicine and Rehabilitation Umeå University Umeå Sweden; ^7^ Umeå School of Sport Sciences Umeå University Umeå Sweden

**Keywords:** aerobic–anaerobic, athletics, biochemical marker, effect, match‐play exposure, performance adaptation, physiological adaptation, physiological stress, pre‐season training, recovery strategy, training intensity

## Abstract

Pre‐season training prepares soccer players for competition, but the longitudinal effects of friendly match exposure remain unclear. We investigated whether cumulative late pre‐season match‐play is associated with physiological adaptation in semi‐professional players. Twenty‐four male players from a randomly selected semi‐professional team competing at national level were assessed at pre‐season start, mid‐pre‐season and pre‐competition. Measurements included body composition, physical performance, aerobic fitness and blood markers reflecting muscle damage, inflammation, hormonal status and haematological adaptation. Based on cumulative playing time across eight friendly matches, as determined by coaching staff, players were classified retrospectively into extended play (EP) or limited play (LP) groups. Controlling for mid‐pre‐season baseline, the EP group showed significantly lower pre‐competition values for body mass ($\eta_{p}^{2}$= 0.276), body fat ($\eta_{p}^{2}$= 0.294) and 35 m sprint time ($\eta_{p}^{2}$= 0.129) and higher values for lower‐limb power (dominant/non‐dominant; $\eta_{p}^{2}$= 0.219–0.424), velocity at maximal oxygen uptake ($\eta_{p}^{2}$= 0.441) and at second ventilatory threshold ($\eta_{p}^{2}$= 0.708), serum glutamic–oxaloacetic transaminase ($\eta_{p}^{2}$= 0.296), myoglobin ($\eta_{p}^{2}$= 0.199), high‐sensitivity C‐reactive protein ($\eta_{p}^{2}$= 0.920), interleukin‐6 ($\eta_{p}^{2}$= 0.893), tumour necrosis factor‐α ($\eta_{p}^{2}$= 0.937) and red blood cell count ($\eta_{p}^{2}$= 0.190) (all *P* ≤ 0.038). Across all participants, total match‐play time was correlated significantly with reductions in body fat (*r* = −0.47) and increases in lower‐limb power (*r* = 0.44–0.67), second ventilatory threshold (*r* = 0.55) and inflammatory markers (*r* = 0.79–0.86) (all *P* ≤ 0.033). Greater friendly match‐play exposure during pre‐season was associated with favourable physiological adaptations and a more pronounced physiological and inflammatory response. These findings support friendly match‐play as a meaningful physiological stimulus and highlight the importance of balancing match exposure with recovery during pre‐season preparation.

## Abstract (highlights)

- **What is the central question of this study?**
  Is cumulative friendly match exposure during late pre‐season associated with differences in multisystem physiological adaptation in semi‐professional soccer players in ecologically valid training conditions?
- **What is the main finding and its importance?**
  Greater cumulative match exposure was associated with coordinated improvements in aerobic efficiency, neuromuscular performance and erythropoietic indices, together with sustained elevations in markers of muscle remodelling and exercise‐related inflammatory activity. These findings suggest that repeated intermittent match‐play is associated with integrated physiological responses reflecting both adaptation and increased recovery demands. This study provides longitudinal human evidence that exposure‐dependent loading is associated with the balance between adaptive and inflammatory responses during intensified preparatory training.

## 1 INTRODUCTION

In modern soccer, the annual training cycle comprises three principal phases: preparatory (pre‐season), competitive and transitional. The pre‐season (∼7–9 weeks) serves as a crucial bridge between off‐season detraining and the high‐intensity, high‐volume demands of competitive play (Williams et al., 2023). It typically includes a general preparation phase (∼3–4 weeks, focused on foundational conditioning), a special preparation phase (∼3–4 weeks, emphasising tactical integration and friendly matches) and a tapering phase (∼1–2 weeks) aimed at optimising readiness for competition (Williams et al., 2023). During this period, repeated exposure to structured training and match‐play provides a potent multisystem stimulus capable of eliciting coordinated adaptations across metabolic, neuromuscular, endocrine, haematological and inflammatory pathways (Mylonis et al., 2025). Characterising these integrative responses is central to understanding how athletes transition from detraining to competitive readiness.

Most research has focused on acute physiological and biochemical responses to isolated matches or training sessions (Ispirlidis et al., 2008; Kostrzewa‐Nowak et al., 2015), whereas fewer investigations have examined longitudinal adaptations across the entire pre‐season period (Mylonis et al., 2025). Moreover, variability in match exposure (well documented during the competitive season; Kraemer et al., 2004) might represent an important yet underexplored modifier of adaptation during pre‐season. First‐team players who accumulate more match time (∼80%) across a competitive season often exhibit enhanced neuromuscular qualities compared with non‐starters (Hoppe et al., 2020; Sanchez‐Sanchez et al., 2019; Sporis et al., 2011), although higher cumulative match loads can also increase injury risk and physiological strain (Jiang et al., 2022). Whether similar exposure‐dependent adaptations occur during the preparatory phase, when training and match stimuli interact dynamically, remains insufficiently understood.

Despite the recognised role of friendly matches as a transitional step between training and formal competition, facilitating tactical execution and team integration (Folgado et al., 2014; Sporis et al., 2011), their contribution as a longitudinal physiological stimulus capable of influencing multisystem adaptation during the pre‐season period has received limited attention. Friendly matches are characterised by repeated high‐intensity actions, substantial locomotor demands and considerable psychophysiological stress, and pre‐season periods incorporating friendly match‐play have been associated with meaningful changes in body composition, aerobic fitness, muscle damage markers and endocrine responses (Caldwell & Peters, 2009; Mylonis et al., 2025). These observations suggest that cumulative match exposure might differentially modulate adaptive remodelling and systemic stress responses. Furthermore, the physiological demands experienced by players are unlikely to be uniform and might vary according to positional role, tactical responsibilities and the timing and duration of match participation. In addition, interindividual differences in baseline fitness, recovery capacity and responsiveness to training might also influence adaptation patterns.

The present study therefore aimed to evaluate longitudinal changes in anthropometric, physiological, biochemical, hormonal and performance markers across the pre‐season period, with specific emphasis on differences associated with cumulative friendly match exposure (extended vs. limited) during the final preparatory phase. By framing match exposure as a naturally occurring variation in training load rather than an assigned intervention, we sought to characterise its association with multisystem physiological adaptation. We hypothesised that: (1) significant within‐group changes would occur across the pre‐season in both exposure groups; (2) distinct exposure‐dependent adaptations would emerge by the end of the preparatory period; and (3) cumulative match exposure would be associated with physiological and performance outcomes at pre‐season completion.

## 2 MATERIALS AND METHODS

### 2.1 Participant recruitment and eligibility

Prior to enrolment (Figure 1), all participants completed medical screening and standardised lifestyle questionnaires assessing smoking status, sleep habits and physical activity (Bourdas & Zacharakis, 2020; Warburton et al., 2011). Inclusion criteria required participants to be male, >18 years old, non‐smokers, injury‐ and medication‐free for the past 6 months, abstinent from alcohol, living <1500 m a.s.l. and committed exclusively to soccer. All were classified as highly active (i.e., >1000 MET‐min week^−1^) and were members of a semi‐professional club competing within the Greek national league system.

**FIGURE 1:**
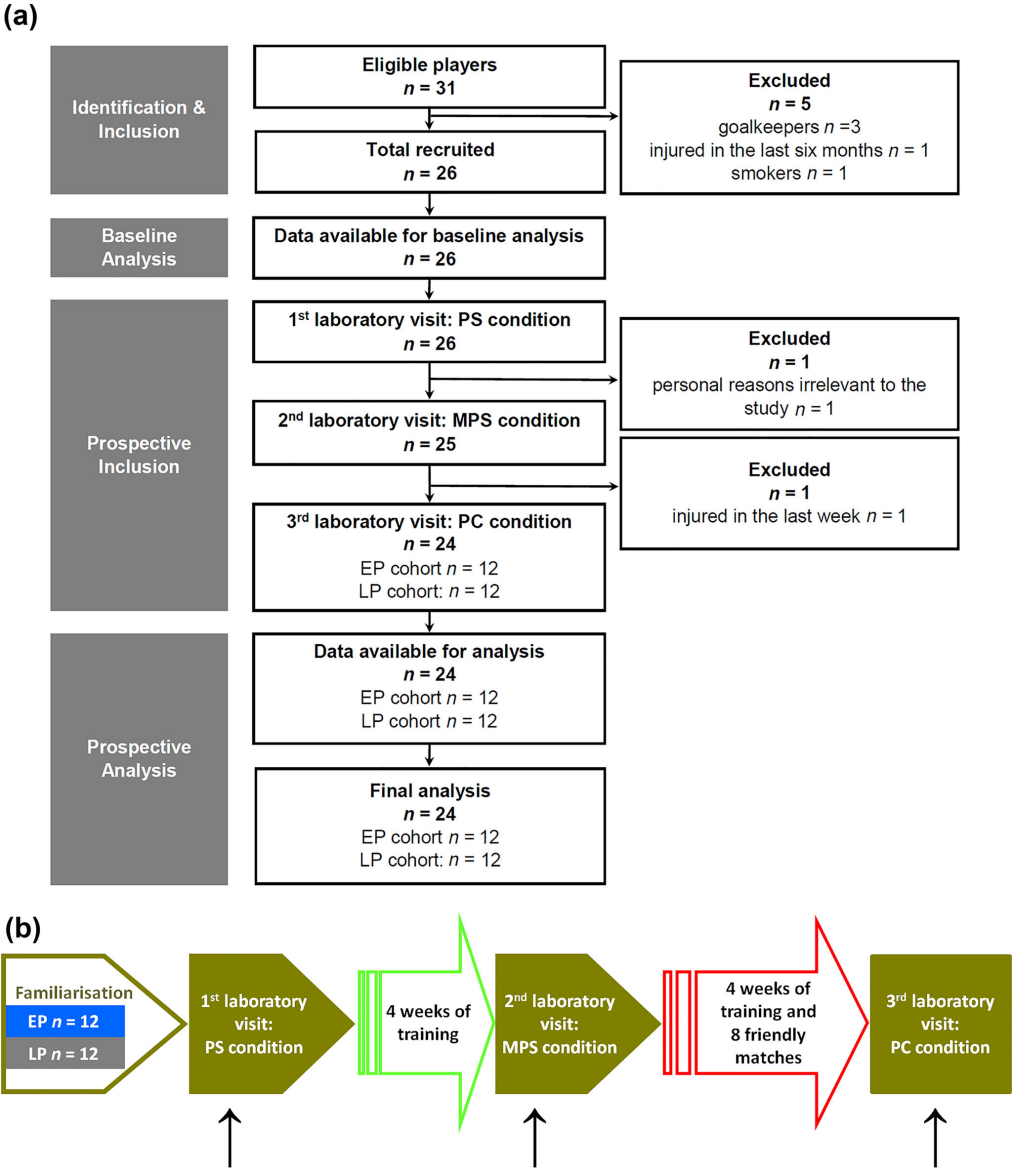
Study design and assessment time line. (a) Flowchart illustrating participant recruitment, group allocation, and progression through the study phases. (b) Schematic overview of the experimental time line, detailing the chronological order and timing of all physiological, biochemical and performance assessments conducted across the preparatory period. Abbreviations: EP, extended play in friendly games during the late pre‐season preparation phase; LP, limited play in friendly games during the late pre‐season preparation phase; MPS, middle of the pre‐season preparation phase; *n*, sample size of soccer players at each stage; PC, 1 week prior to the initiation of the competition phase; PS, prior to the initiation of the 9 weeks of pre‐season preparation phase; ↑, anthropometric, physiological, biochemical, hormonal and performance‐related assessments.

Male and female soccer teams from Greek first, second and third division (*n* = 201) were invited via email to their respective clubs. Of the three interested male teams (one second and two third division), one was selected randomly using RANDBETWEEN function of Excel. From the 33 eligible players on this team, 31 consented to participate, with 7 later excluded owing to ineligibility or withdrawal. The final sample consisted of 24 outfield players.

After completion of data collection, players were classified retrospectively into two cohorts according to cumulative playing time accrued across eight friendly matches (two per week) during the final pre‐season month, reflecting coaching staff decisions rather than investigator‐assigned exposure, with group allocation determined entirely by naturally occurring match exposure and coaching decisions rather than investigator assignment. Demographic details are provided in Table 1. For analytical purposes, a 40% participation threshold was used as an operational cut‐point to distinguish players with relatively greater versus limited match exposure. This threshold was selected a priori to provide two cohorts approximately equal in size while maintaining a clear separation in cumulative match participation and preserving the naturally occurring distribution of playing time determined by the coaching staff. The 40% threshold was therefore used as a pragmatic classification criterion rather than as a prespecified biological or clinical threshold. Importantly, no player's match participation was modified to achieve the classification. The extended play (EP) cohort, comprising 12 players who received ≥40% match exposure, consisted of two central defenders, two full‐backs, two central midfielders, three wide midfielders and three attackers and accumulated 494.08 ± 112.61 min, corresponding to 68.62% ± 15.64% of available minutes. The limited play (LP) cohort, comprising 12 players who received <40% match exposure, consisted of two central defenders, three full‐backs, one central midfielder, three wide midfielders and three attackers and accumulated 145.75 ± 89.61 min, corresponding to 20.24% ± 12.45% of available minutes. Cumulative match exposure differed significantly between cohorts (*P *< 0.001). The matches included two against higher‐level opponents, two against teams of comparable level and four against lower‐level teams (≥3 days between matches).

**TABLE 1:** Demographic details, characteristics, experience and training load data of the EP and LP cohorts, presented as the mean ± SD [95% confidence interval]

Variable	EP (*n* = 12)	LP (*n* = 12)
Age, years	26.42 ± 3.55[24.41–28.43]	20.25 ± 2.45 [18.86–21.64]
Height, cm	177.92 ± 5.26[174.94–180.9]	180.08 ± 5.33 [177.06–183.10]
Body mass, kg	76.71 ± 5.01 [73.88–79.54]	72.24 ± 8.19 [67.61–76.87]
Body fat, %	12.49 ± 1.81 [11.47–13.51]	12.32 ± 3.31 [10.45–14.19]
Experience practising soccer, years	11.67 ± 2.81 [10.08–13.26]	8.75 ± 1.36 [7.98–9.52]
Active in soccer competition, years	4.92 ± 2.75 [3.36–6.48]	1.42 ± 0.67 [1.04–1.80]
Individual (non‐soccer) extra physical and resistance exercise training, h week^−1^	2.50 ± 0.52 [2.21–2.79]	2.42 ± 0.51 [2.13–2.71]

Abbreviations: EP, extended play in friendly games during the late pre‐season preparation phase; LP, limited play in friendly games during the late pre‐season preparation phase.

### 2.2 Equity, diversity and inclusion statement

The authorship team were gender diverse, comprising researchers at various career stages (from graduate level to senior academics) based in Greece and Sweden. The team brought interdisciplinary expertise spanning sports medicine, exercise physiology, statistics, sport science, and strength and conditioning. Although the study was conducted with a male soccer team, efforts were made to recruit female teams of comparable competitive level. However, constraints such as non‐responsiveness and/or declined participation limited the inclusion of female participants.

### 2.3 Experimental design and procedures

To ensure reliability, players visited the laboratory 1 week before the baseline for familiarisation. Data were collected at three key time points during the 9 week pre‐season period: PS (pre‐season start), baseline testing before training onset; MPS (mid‐pre‐season), after 4 weeks of structured training (no match‐play); and PC (pre‐competition), after an additional 4 weeks, including friendly matches, conducted ≥6 days after the final match.

Participants adhered to standardised dietary and behavioural controls throughout the study. To minimise dietary variability, participants followed a balanced macronutrient diet (50%–60% carbohydrates, 25%–30% fats and 15% protein). Three standardised meals (breakfast, lunch and dinner) were provided daily throughout the study period and were formulated to meet the prescribed macronutrient distribution. Participants were instructed to consume only the provided meals and to record any additional food or beverage intake in detailed dietary diaries. Food records were reviewed regularly to assess adherence and document deviations from the prescribed diet. The evening meal prior to testing included ∼65% carbohydrates (e.g., 100 g pasta, 180 g chicken and one banana) (Mylonis et al., 2025). Testing occurred in a fasted state between 09:00 and 11:00 h to minimise circadian variability. Alcohol, medication and ergogenic aid use were prohibited for 1 week prior (Bourdas et al., 2021, 2024). Intense activity was avoided for 48 h before testing; only light technical/tactical sessions were allowed. Sleep, health status and altitude exposure were controlled.

Blinding procedures were implemented to minimise potential measurement and expectancy bias. Participants were not informed of the specific hypotheses concerning differential physiological responses according to cumulative friendly match exposure, and assessors were blinded to the exposure‐group classification of participants during outcome assessment. Individual test results were not disclosed to participants during the study. Testing was conducted in a climate‐controlled laboratory (22°C–25°C and 55%–65% humidity), with all equipment calibrated before each session.

Assessments were completed on the same day at each time point, with ≥10 min of rest between tests. Maximal oxygen uptake (${\dot{V}}_{O_{2}\max}$) testing was scheduled last, following a 30 min passive recovery to limit effects of fatigue. This standardised schedule minimised inter‐session variability and logistical constraints linked to team schedules.

All participants followed an identical soccer‐specific team training programme (∼12 h week^−1^) throughout the study period, as prescribed and supervised by the team's coaching staff. The overview of the players’ training programme, as provided by the coaching staff, is available in the supplementary materials (file: Training program overview.pdf).

### 2.4 Measurements

Participants were instructed to void their bladder prior to performance testing and had unrestricted access to water.

#### 2.4.1 Anthropometrics and body composition

Body mass was determined to the nearest 0.01 kg using a beam balance scale (Beam Balance 710, Seca, Birmingham, UK), with participants lightly clothed in shorts and a t‐shirt. Stature was measured with a Stadiometer^®^ (Seca, Birmingham, UK) following a full inhalation and with shoes removed. Skinfold thickness was recorded at four anatomical sites (biceps, triceps, subscapular and suprailiac regions) using calibrated Harpenden callipers (Baty International, UK). Body fat percentage was estimated using the equations of Durnin and Womersley (1974) according to the age of the participant. Each variable was measured twice, and their average was used unless discrepancies exceeded thresholds of 0.4 g (for mass), 4 mm (for height) or 2 mm (for skinfolds), in which case a third measurement was taken and the median value recorded.

#### 2.4.2 Perceived exertion

The subjective rating of perceived exertion was assessed using the modified Borg CR10 scale (Borg, 1982) to monitor perceived muscular fatigue and overall exertional load.

#### 2.4.3 Haematological and biochemical markers

Venous blood samples were obtained at a consistent time of day, in a seated position following an overnight fast. Samples were obtained from the basilic or mesobasilic vein. For haematological and biochemical analysis (i.e., red blood cell count, haemoglobin, haematocrit, iron, glucose, cholesterol and triglycerides), 10 µL aliquots were applied to specialised cuvettes (LKM 142, 143, 144, 130, 141, 226 and 227; Dr. Lange, Hamburg, Germany) and analysed with a spectrophotometer (Miniphotometer Plus LP20, Dr. Lange). A complete blood count, including white blood cell (leucocyte) and platelet numbers, was measured from EDTA‐K3 tubes using a Coulter MAXM analyser (Beckman Coulter, Indianapolis, IN, USA). Serum testosterone levels were measured with an ADVIA Centaur XP immunoassay system (Siemens, Malvern, PA, USA). ELISA kits (Elabscience, Houston, TX, USA) were used to quantify cortisol (E‐EL‐0157), interleukin‐6 (E‐EL‐H6156) and tumour necrosis factor‐α (TNF‐α; E‐EL‐H0109), read via a standard microplate reader (Spark 10M^®^, Tecan, Männedorf, Switzerland). C‐Reactive protein and high‐sensitivity C‐reactive protein (hs‐CRP) were evaluated using COBAS e411^®^ and c111^®^ immunoassay analysers (F. Hoffmann‐La Roche Ltd, Rotkreuz, Switzerland), respectively. Serum creatine kinase activity was determined with Roche CK test strips^®^ and a Reflotron Plus System^®^ (Roche, Basel, Switzerland). Serum transaminase levels (serum glutamic–oxaloacetic transaminase and serum glutamic–pyruvic transaminase) were assessed using the SMAC automated system with Reference II reagents (Technicon Corp., Tarrytown, NY, USA) (Zanardo et al., 1985). Myoglobin concentrations were determined via the IA‐100 immunoassay system (Sysmex, Kobe, Japan), following Dupuy et al. (2017) and Saita et al. (2023). Ferritin analysis was performed on serum obtained from centrifuged blood samples (360 *g*, 10 min) stored at −20°C and later analysed with a radioimmunoassay kit (Bio‐Rad Laboratories, Hemel Hempstead, UK). All tests were run in duplicate, and mean values were used. Reported intra‐ and interassay coefficients of variation were within 2.4%–8.7% and 3.5%–8.9%, respectively, per manufacturer documentation.

#### 2.4.4 Flexibility

Lower back and hamstring flexibility was evaluated using the standard sit‐and‐reach test. Participants completed a general warm‐up involving 10 min of light aerobic activity and full‐body dynamic/static stretching. Using a purpose‐built sit‐and‐reach box (60 cm×35 cm×35 cm; Cranlea Human Performance Ltd, Birmingham, UK), participants, barefoot and with legs extended, reached forwards with the hands aligned and palms down. The furthest reach of three attempts (separated by 1 min rests) was recorded for analysis, following established protocols (Holt et al., 1999).

#### 2.4.5 Neuromuscular performance

Lower‐limb explosive power was assessed with a countermovement jump test (Bosco et al., 1983) following a 5 min warm‐up on a leg ergometer (894E^®^, Monark, Varberg, Sweden), performed at 60%–70% of age‐predicted maximal heart rate (HR_max_; calculated using HR_max_ = 211 − 0.64 × age) (Nes et al., 2013) and a self‐paced cadence (50–75 rpm). Participants performed three maximal vertical countermovement jumps, allowing a 1 min reset between trials, with arm swing on a contact timing mat (ERGO JUMP Plus—BOSCO SYSTEM, Byomedic, S.C.P., Barcelona, Spain), maintaining a squat start (∼90° knee angle) and neutral upper‐body posture (Luhtanen & Komi, 1978). The highest jump height was recorded.

Next, isotonic‐ballistic testing of knee flexor/extensor strength was conducted using an Ergopower dynamometer (Ergotest Technology A.S., Langesund, Norway). Participants performed three maximal flexion–extension efforts with a 50% submaximal load, each separated by 20 s (Bosco et al., 1995). The peak power value was retained for analysis.

#### 2.4.6 Sprint and change of direction speed

After a warm‐up (5 min) including jogging and short sprints, participants completed three maximal 10 m sprints from a standing start, with the lead foot placed 30 cm behind the start line. Sprint times were measured using photoelectric cells (height = 110 cm; Saint Wien Digital Timer Press H5K, Taipei, Taiwan) placed at start and finish lines. Time resolution was 0.01 s, with ±0.01 s accuracy. The fastest time was used in statistical analysis.

Change of direction speed (CODS) was assessed using the Illinois agility test (Hastad & Lacy, 1994). The test layout measured 10 m in length and 5 m in width, with cones marking the start, finish, turning points and central weaving path (four cones spaced 3.3 m apart). Each participant completed three trials from a standing start (as described above). Timing was again conducted using paired infrared gates, and the best result was retained for further use.

#### 2.4.7 Anaerobic performance

Participants’ peak running velocity was determined through the running‐based anaerobic sprint test (RAST) (Zacharogiannis et al., 2004). The assessment took place on an indoor synthetic turf pitch used for small‐sided soccer (57 m × 37 m; Prestige XM6 60‐13, FieldTurf^®^, Tarkett Sports, Paris, France). The test comprised six maximal effort sprints over 35 m, delineated with marker cones, each separated by a 10 s passive recovery. Athletes initiated each sprint from a standing start on verbal command, alternating the starting side after each run. Sprint times were recorded using the same infrared photoelectric timing gates described earlier (see section 2.4.6).

The RAST was selected owing to its strong associations with the Wingate anaerobic test and its proven consistency in evaluating anaerobic performance and fatigue characteristics in field settings (Zagatto et al., 2009). Unlike the traditional power‐based RAST outputs (e.g., peak/mean power, fatigue index), this study emphasised two time‐dependent metrics: the mean sprint time across all six efforts and a fatigue‐related speed decrement ratio, calculated as speed drop (%) = [(slowest sprint time − fastest sprint time)/fastest sprint time] × 100. These outcome variables were chosen for their enhanced ecological validity, improved resistance to variations in body weight and minor timing inconsistencies, and their greater interpretability for practitioners in applied sport environments.

#### 2.4.8 Aerobic capacity and ventilatory threshold

To determine maximal aerobic capacity (${\dot{V}}_{O_{2}\max}$), participants performed a graded exercise test to voluntary exhaustion on a calibrated motorised treadmill (Technogym Runrace^®^ 1200, Technogym, Gambettola, Italy). Following a 5 min warm‐up at a 0% incline, the test began at 7 km h^−1^ for 60 s, then increased to 8 km h^−1^ for 30 s. From this point, speed was incremented by 0.5 km h^−1^ every 30 s until volitional fatigue, with the incline set at 1%. The use of short (30 s) stages was selected to provide a more fine‐grained assessment of the oxygen uptake (${\dot{V}}_{O_{2}}$) response and ventilatory thresholds in trained soccer players, who might reach steady state rapidly (Robergs et al., 2010).

The ${\dot{V}}_{O_{2}\max}$ and HR_max_ were identified as the highest 15 and 5 s averages, respectively, recorded in the final stages of the test. Standard criteria used to confirm attainment of ${\dot{V}}_{O_{2}\max}$ included a respiratory exchange ratio of >1.10, heart rate within 10 beats min^−1^ of age‐predicted HR_max_, reported exertion ≥18 on the Borg scale (6–20) (Borg, 1982) and/or a plateau in ${\dot{V}}_{O_{2}}$ despite increased workload (<2 mL kg^−1^ min^−1^ change), as recommended by the American College of Sports Medicine (2018).

The second ventilatory threshold (VT_2_) was also determined using expert judgement. Two blinded evaluators independently identified VT_2_ based on changes in ventilatory equivalents and end‐tidal gas values, referencing standard criteria (Cerezuela‐Espejo et al., 2018; Gaskill et al., 2001; Wasserman et al., 1973). Discrepancies were resolved through averaging. The VT_2_ was subsequently expressed in terms of both velocity and heart rate as indicators of aerobic performance.

Gas exchange during the graded exercise test was analysed via calibrated gas analysers (OM‐11 and LB‐2, Beckman Instruments Inc., Indianapolis, IN, USA) using standardised gas concentrations of O_2_ and CO_2_ for calibration.

Heart rate was monitored continuously at 5 s intervals using a wireless telemetry system (Polar Sport Tester, Polar Electro Oy, Kempele, Finland). Capillary blood samples (5–25 µL) were obtained from the left index finger 10 min postexercise to measure blood lactate (La^−^) concentration using a hand‐held device (Lactate Plus, Nova Biomedical, Waltham, MA, USA). Each lactate sample was tested in duplicate, and the average was used for analysis. Manufacturer‐validated coefficients of variation for intra‐ and interassay precision ranged from 3.4% to 5.9% within the 2.6–10.5 mmol L^−1^ measurement range (Nova Biomedical, 2011).

### 2.5 Statistical analyses

Data normality and homogeneity were assessed using the Shapiro–Wilk test and Levene's test, respectively. In addition, assumptions underlying the inferential models were evaluated prior to analysis. For repeated‐measures ANOVA, normality of residuals and sphericity were assessed, with Greenhouse–Geisser corrections applied when the assumption of sphericity was violated. For ANCOVA, linearity of relationships between covariates and dependent variables, homogeneity of regression slopes and homogeneity of variances were examined before model implementation.

The student's unpaired *t*‐test was used to evaluate differences between cohorts in cumulative match‐play time. One‐way repeated‐measures ANOVAs were conducted to locate differences among PS, MPS and PC for all dependent variables for EP and LP cohorts. Greenhouse–Geisser *e* corrections were applied where necessary. Significant main effects were examined further with Bonferroni‐adjusted comparisons.

For between‐group comparisons in the PC condition, one‐way ANCOVAs were conducted, with baseline MPS values as covariates; significant effects were followed by Bonferroni *post hoc* tests. The magnitude of observed effects was quantified using partial eta squared ($\eta_{p}^{2}$). Effect sizes were interpreted according to commonly accepted thresholds for $\eta_{p}^{2}$: small (≥0.01), medium (≥0.06) and large (≥0.14) (Cohen et al., 2018). Because $\eta_{p}^{2}$ can be influenced by model structure and sample characteristics, effect‐size estimates were considered together with their confidence intervals, *P*‐values and the consistency of findings across related outcomes rather than as standalone evidence of practical importance. Given the exploratory and integrative nature of the study, involving multiple physiologically related outcome variables, the number of outcomes and statistical comparisons was considered when interpreting the findings. Accordingly, statistical inference was not based solely on *P*‐values, and individual statistically significant results were not interpreted in isolation.

Pearson correlations assessed relationships between cumulative match‐play time and physiological, biochemical and performance markers in the PC condition across all participants. All analyses were performed using SPSS v.29.0 (IBM Corp., Armonk, NY, USA), with statistical significance set at *P *< 0.05. Where appropriate, 95% confidence intervals were calculated and are reported for key outcome measures to facilitate interpretation of estimate precision.

Furthermore, a *post hoc* power analysis (GraphPad StatMate v.2.0, GraphPad Software Inc., La Jolla, CA, USA) assessed sample size adequacy based on mean body mass ($\eta_{p}^{2}$= 0.276; α = 0.05; *n* = 24) in a between‐group design (extended vs. limited match‐play). Observed power (1 − β) at the PC point exceeded 0.80. Similar power values were found for body fat, myoglobin, hs‐CRP, interleukin‐6, TNF‐α, red blood cell count, lower‐limb power (dominant/non‐dominant), average 35 m time (RAST), and velocities at ${\dot{V}}_{O_{2}\max}$ ($v{\dot{V}}_{O_{2}\max}$) and VT_2_ (*v*VT_2_).

## 3 RESULTS

Descriptive statistics for all variables (encompassing physical, biochemical, hormonal, haematological and performance domains) across PS, MPS and PC time points are summarised in Tables A1, A2 for the EP and LP cohorts, respectively. Detailed Bonferroni‐adjusted pairwise comparisons (PS vs. MPS, PS vs. PC and MPS vs. PC), including P‐values, Cohen's d and 95% confidence intervals for each variable within each cohort, are available in Tables A3, A4, A5, A6, respectively. Percentage changes between time points are illustrated in Figure 2. Across the pre‐season period, both cohorts demonstrated changes in several anthropometric, performance, biochemical and haematological variables, although the magnitude and direction of these responses differed between groups (Figure 2; Tables A3, A4, A5, A6). In general, players with greater friendly match exposure exhibited larger improvements in aerobic and neuromuscular measures, accompanied by greater elevations in markers associated with muscle stress and inflammatory activity. These patterns were considered in conjunction with the magnitude and precision of the estimated effects rather than on the basis of statistical significance alone. One‐way ANCOVAs (adjusting for MPS baseline) revealed significantly greater PC values in the EP cohort compared with LP for knee extension/flexion power (dominant and non‐dominant legs) (*P* = 0.001–0.014, $\eta_{p}^{2}$= 0.219‐0.424), $v{\dot{V}}_{O_{2}\max}$ (*P* = 0.001, $\eta_{p}^{2}$= 0.441), *v*VT_2_ (*P *< 0.001, $\eta_{p}^{2}$= 0.708), serum glutamic–oxaloacetic transaminase (*P* = 0.007, $\eta_{p}^{2}$= 0.296), myoglobin (*P* = 0.033, $\eta_{p}^{2}$= 0.199), hs‐CRP (*P *< 0.001, $\eta_{p}^{2}$= 0.920), interleukin‐6 (*P *< 0.001, $\eta_{p}^{2}$= 0.893), TNF‐α (*P *< 0.001, $\eta_{p}^{2}$= 0.937) and red blood cell count (*P* = 0.038, $\eta_{p}^{2}$= 0.190), with significantly lower‐body mass (*P* = 0.010, $\eta_{p}^{2}$= 0.276), body fat percentage (*P* = 0.008, $\eta_{p}^{2}$= 0.294), average 35 m RAST time (*P *< 0.001, $\eta_{p}^{2}$= 0.474) and lactate at ${\dot{V}}_{O_{2}\max}$ (*P* = 0.007, $\eta_{p}^{2}$= 0.300) (Table 2). Finally, in the full sample (*n* = 24), total match‐play time in friendly games showed significant correlations with multiple variables, including negative associations with body fat (*r* = –0.47, *P* = 0.020), lactate at ${\dot{V}}_{O_{2}\max}$ (*r* = –0.62, *P* = 0.001) and serum iron (*r* = –0.44, *P* = 0.030); and positive associations with lower‐limb power (*r* = 0.44–0.67, P ≤0.033), *v*VT_2_ (*r* = 0.55, P = 0.005), ferritin (*r* = 0.44, P = 0.031), hs‐CRP (*r* = 0.86, P < 0.001), interleukin‐6 (*r* = 0.79, P < 0.001) and TNF‐α (*r* = 0.82, P < 0.001) (Table A7).

**FIGURE 2:**
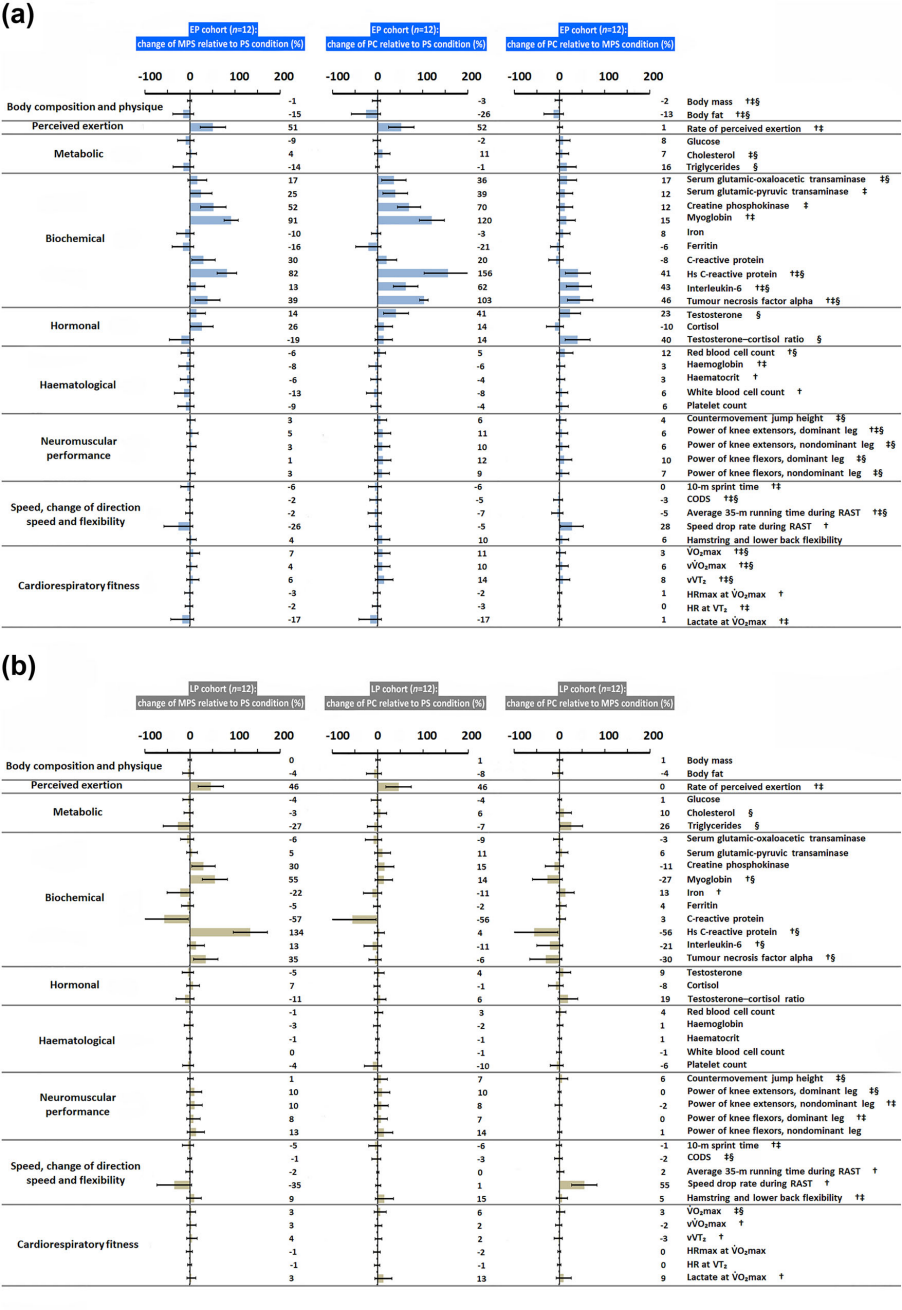
Depiction of changes in mean values for physical, haematological, hormonal, biochemical and performance parameters from PS to MPS (left), from PS to PC (centre) and from MPS to PC (right) for the EP cohort (a) and the LP cohort (b). Error bars present the lower and upper bounds of the 95% confidence interval. †Significant difference between PS and MPS conditions at *P *< 0.05. ‡Significant difference between PS and PC conditions at *P *< 0.05. §Significant difference between MPS and PC conditions at *P *< 0.05. Abbreviations:CODS, change of direction speed; EP, extended play in friendly games during the late pre‐season preparation phase; HR, heart rate; HR_max_, maximal heart rate; LP, limited play in friendly games during the late pre‐season preparation phase; MPS, middle of the pre‐season preparation phase; *n*, sample size; PC, 1 week prior to the initiation of the competition phase; PS, prior to initiation of the 9 weeks of pre‐season preparation phase; RAST, running‐based anaerobic sprint test; ${\dot{V}}_{O_{2}\max}$, maximal oxygen uptake; VT_2_, second ventilatory threshold; $v{\dot{V}}_{O_{2}\max}$, velocity at ${\dot{V}}_{O_{2}\max}$; *v*VT_2_, velocity at VT_2_.

**TABLE 2:** Physical, biochemical, hormonal, haematological and performance estimates in the PC conditions in the EP and LP cohorts according to participants’ time playing in friendly games, adjusted for the covariate^†^ values of the MPS condition

Variable	Cohort: frequency (%)	Measurement estimates (mean ± SE [95% CI])	$\eta_{p}^{2}$	Significance (*P*‐value)	†MPS value used to evaluate covariates in the model
Body mass, kg^*^	EP: 12 (50.00)	72.80 ± 0.47 [71.81–73.79]	0.276	0.010	74.0417
	LP: 12 (50.00)	74.74 ± 0.47 [73.76–75.73]			
Body fat, %^*^	EP: 12 (50.00)	9.66 ± 0.30 [9.04–10.28]	0.294	0.008	11.2092
	LP: 12 (50.00)	10.92 ± 0.3 [10.30–11.54]			
Rating of perceived exertion, Borg scale (1–10)	EP: 12 (50.00)	7.50 ± 0.20 [7.09–7.92]	<0.001	0.980	7.4583
	LP: 12 (50.00)	7.50 ± 0.20 [7.08–7.91]			
Glucose, mg dL^−1^	EP: 12 (50.00)	89.19 ± 1.81 [85.43–92.95]	0.165	0.055	83.0417
	LP: 12 (50.00)	83.98 ± 1.81 [80.22–87.74]			
Cholesterol, mg dL^−1^	EP: 12 (50.00)	168.19 ± 3.74 [160.42–175.96]	0.027	0.454	157.3750
	LP: 12 (50.00)	172.23 ± 3.74 [164.46–180]			
Τriglycerides, mg dL^−1^	EP: 12 (50.00)	85.33 ± 5.18 [74.55–96.12]	0.031	0.423	72.8750
	LP: 12 (50.00)	91.33 ± 5.18 [80.55–102.11]			
Serum glutamic–oxaloacetic transaminase, U L^−1^ ^*^	EP: 12 (50.00)	30.62 ± 1.24 [28.05–33.18]	0.296	0.007	26.2917
	LP: 12 (50.00)	25.38 ± 1.24 [22.82–27.95]			
Serum glutamic–pyruvic transaminase, U L^−1^	EP: 12 (50.00)	26.74 ± 1.55 [23.52–29.95]	0.019	0.532	23.9583
	LP: 12 (50.00)	25.35 ± 1.55 [22.13–28.56]			
Creatine phosphokinase, U L^−1^	EP: 12 (50.00)	284.48 ± 25.16 [232.16–336.8]	0.111	0.120	258.8750
	LP: 12 (50.00)	222.85 ± 25.16 [170.54–275.17]			
Myoglobin, mg L^−1^ ^*^	EP: 12 (50.00)	100.1 ± 10.2 [78.89–121.31]	0.199	0.033	91.7083
	LP: 12 (50.00)	64.57 ± 10.2 [43.36–85.77]			
Iron, µg dL^−1^	EP: 12 (50.00)	85.82 ± 5.93 [73.5–98.15]	0.089	0.167	82.9583
	LP: 12 (50.00)	98.26 ± 5.93 [85.93–110.59]			
Ferritin, mg L^−1^	EP: 12 (50.00)	62.17 ± 4.71 [52.38–71.96]	<0.001	0.967	63.6708
	LP: 12 (50.00)	62.48 ± 4.71 [52.69–72.27]			
C‐Reactive protein, mg L^−1^	EP: 12 (50.00)	0.34 ± 0.03 [0.28–0.39]	<0.001	0.927	0.3508
	LP: 12 (50.00)	0.34 ± 0.03 [0.28–0.39]			
High‐sensitivity C‐reactive protein, mg L^−1^ ^*^	EP: 12 (50.00)	2.3 ± 0.07 [2.16–2.44]	0.920	<0.001	1.7083
	LP: 12 (50.00)	0.78 ± 0.07 [0.64–0.92]			
Interleukin‐6, mg L^−1^ ^*^	EP: 12 (50.00)	13.27 ± 0.35 [12.55–13.99]	0.893	<0.001	8.6208
	LP: 12 (50.00)	6.52 ± 0.35 [5.8–7.24]			
Tumour necrosis factor‐α, pg mL^−1^ ^*^	EP: 12 (50.00)	11.59 ± 0.22 [11.14–12.04]	0.937	<0.001	8.2833
	LP: 12 (50.00)	6.01 ± 0.22 [5.56–6.46]			
Testosterone, ng mL^−1^	EP: 12 (50.00)	6.69 ± 0.42 [5.81–7.57]	0.062	0.254	5.4663
	LP: 12 (50.00)	5.99 ± 0.42 [5.1–6.87]			
Cortisol, µg dL^−1^	EP: 12 (50.00)	16.21 ± 1.09 [13.94–18.48]	0.001	0.889	17.9296
	LP: 12 (50.00)	16.43 ± 1.09 [14.16–18.7]			
Testosterone:cortisol ratio (T:C)	EP: 12 (50.00)	0.45 ± 0.03 [0.38–0.51]	0.075	0.205	0.3167
	LP: 12 (50.00)	0.39 ± 0.03 [0.32–0.45]			
Red blood cell count, ×10^12^ L^−1^ ^*^	EP: 12 (50.00)	5.75 ± 0.12 [5.5–5.99]	0.190	0.038	5.1625
	LP: 12 (50.00)	5.37 ± 0.12 [5.13–5.62]			
Haemoglobin, g dL^−1^	EP: 12 (50.00)	14.81 ± 0.23 [14.33–15.29]	0.002	0.844	14.4833
	LP: 12 (50.00)	14.74 ± 0.23 [14.26–15.22]			
Haematocrit, %	EP: 12 (50.00)	44.99 ± 0.74 [43.46–46.53]	0.011	0.635	44.0250
	LP: 12 (50.00)	44.48 ± 0.74 [42.95–46.01]			
White blood cell count, ×10^9^ L^−1^	EP: 12 (50.00)	6.59 ± 0.28 [6.02–7.16]	0.043	0.343	6.2583
	LP: 12 (50.00)	6.21 ± 0.28 [5.64–6.78]			
Platelet count, ×10^9^ L^−1^	EP: 12 (50.00)	221.41 ± 13.15 [194.06–248.76]	0.040	0.359	213.1575
	LP: 12 (50.00)	203.92 ± 13.15 [176.57–231.27]			
Countermovement jump height, cm	EP: 12 (50.00)	41.74 ± 0.44 [40.83–42.64]	0.035	0.390	40.1333
	LP: 12 (50.00)	42.28 ± 0.44 [41.37–43.19]			
Power of knee extensors, dominant leg, W^*^	EP: 12 (50.00)	212.66 ± 3.16 [206.08–219.24]	0.254	0.014	200.3333
	LP: 12 (50.00)	200.17 ± 3.16 [193.59–206.75]			
Power of knee extensors, non‐dominant leg, W^*^	EP: 12 (50.00)	217.62 ± 3.16 [211.04–224.19]	0.424	0.001	203.9167
	LP: 12 (50.00)	199.88 ± 3.16 [193.31–206.46]			
Power of knee flexors, dominant leg, W^*^	EP: 12 (50.00)	157.18 ± 4.26 [148.32–166.04]	0.367	0.002	138.8333
	LP: 12 (50.00)	134.82 ± 4.26 [125.96–143.68]			
Power of knee flexors, non‐dominant leg, W^*^	EP: 12 (50.00)	145.6 ± 4.06 [137.15–154.06]	0.219	0.003	133.1250
	LP: 12 (50.00)	131.06 ± 4.06 [122.61–139.52]			
10 m sprint time, s	EP: 12 (50.00)	1.71 ± 0.01 [1.68–1.74]	0.129	0.093	1.6996
	LP: 12 (50.00)	1.68 ± 0.01 [1.65–1.70]			
CODS, s	EP: 12 (50.00)	15.82 ± 0.08 [15.64–15.99]	0.081	0.187	16.3392
	LP: 12 (50.00)	15.98 ± 0.08 [15.81–16.15]			
Average 35 m running time during RAST, s^*^	EP: 12 (50.00)	4.81 ± 0.06 [4.70–4.93]	0.474	<0.001	5.0508
	LP: 12 (50.00)	5.15 ± 0.06 [5.04–5.27]			
Speed drop during RAST, %	EP: 12 (50.00)	9.56 ± 0.92 [7.64–11.48]	0.003	0.808	6.7108
	LP: 12 (50.00)	9.23 ± 0.92 [7.31–11.15]			
Hamstring and lower back flexibility, cm	EP: 12 (50.00)	25.53 ± 0.78 [23.9–27.16]	0.020	0.519	23.7500
	LP: 12 (50.00)	24.80 ± 0.78 [23.18–26.43]			
${\dot{V}}_{O_{2}\max}$, mL kg^−1^ min^−1^	EP: 12 (50.00)	57.41 ± 0.41 [56.55–58.26]	0.006	0.734	55.6271
	LP: 12 (50.00)	57.21 ± 0.41 [56.35–58.06]			
$v{\dot{V}}_{O_{2}\max}$, km h^−1^ ^*^	EP: 12 (50.00)	17.59 ± 0.22 [17.14–18.05]	0.441	0.001	16.5833
	LP: 12 (50.00)	16.32 ± 0.22 [15.86–16.78]			
*v*VT_2_, km h^−1^ ^*^	EP: 12 (50.00)	13.79 ± 0.14 [13.50–14.07]	0.708	<0.001	12.7500
	LP: 12 (50.00)	12.40 ± 0.14 [12.11–12.68]			
HR_max_ at ${\dot{V}}_{O_{2}\max}$, beats min^−1^	EP: 12 (50.00)	193.72 ± 1.24 [191.15–196.30]	0.061	0.257	192.3333
	LP: 12 (50.00)	191.61 ± 1.24 [189.04–194.19]			
HR at VT_2_, beats min^−1^	EP: 12 (50.00)	165.23 ± 1.04 [163.07–167.39]	0.003	0.805	165.7500
	LP: 12 (50.00)	164.85 ± 1.04 [162.69–167.01]			
Lactate at ${\dot{V}}_{O_{2}\max}$, mmol L^−1^ ^*^	EP: 12 (50.00)	10.94 ± 0.38 [10.15–11.72]	0.300	0.007	11.1708
	LP: 12 (50.00)	12.57 ± 0.38 [11.78–13.36]			

Abbreviations: CI, confidence interval; CODS, change of direction speed; EP, extended play in friendly games during the late pre‐season preparation phase; LP, limited play in friendly games during the late pre‐season preparation phase; M, mean; MPS, middle of the pre‐season preparation phase; PC, 1 week prior to the initiation of the competition phase; ${\dot{V}}_{O_{2}\max}$, maximal oxygen uptake; VT_2_, second ventilatory threshold; $v{\dot{V}}_{O_{2}\max}$, velocity at ${\dot{V}}_{O_{2}\max}$; *v*VT_2_, velocity at VT_2_; $\eta_{p}^{2}$, partial eta squared (e.g., 0.01, small effect; 0.06, medium effect; 0.14, large effect).

* *P* < 0.05, significant effect of time played in friendly games on measurement cohorts.

## 4 DISCUSSION

This study extends limited evidence regarding how cumulative match exposure during the pre‐season period is associated with physiological adaptation in soccer. Through longitudinal assessment of anthropometric, neuromuscular, aerobic, biochemical, endocrine, inflammatory and haematological indices, we observed that greater late‐phase match exposure was consistently associated with enhanced functional adaptations alongside sustained elevations in markers of muscle disruption and exercise‐related inflammatory activity. Importantly, these associations were evident across interrelated physiological domains, supporting the presence of coordinated multisystem responses rather than isolated changes in performance.

Given that match exposure was determined by routine coaching decisions rather than experimental allocation, the findings should be interpreted as reflecting exposure‐dependent associations within a real‐world training environment. Because exposure was classified retrospectively and not assigned randomly, selection bias and residual confounding arising from pre‐existing differences between players cannot be excluded. Players receiving greater match exposure might have differed systematically from those receiving limited exposure in characteristics that influenced coaching decisions, such as fitness, playing performance, tactical or positional role, experience or perceived readiness. Although participants were drawn from the same team, competitive level, training programme and seasonal context, these factors were not controlled experimentally and might therefore have contributed to the observed between‐cohort differences. Consequently, causal inferences regarding the effects of match‐play exposure cannot be established. Collectively, these observations reinforce the concept that greater friendly match exposure during pre‐season is associated with both adaptive physiological responses and cumulative physiological demands within the broader context of team training, highlighting the dynamic balance between remodelling and strain during intensified preparatory loading.

### 4.1 Body composition and match exposure

Body fat typically increases during the off‐season and declines with pre‐season training in soccer athletes (Mylonis et al., 2025). In this study, the EP cohort exhibited greater reductions in body mass and fat compared with the LP cohort, aligning with prior findings in elite players (Caldwell & Peters, 2009; Hoppe et al., 2020; Milanese et al., 2015). The greater cumulative match exposure in the EP cohort might have increased the overall metabolic stimulus associated with match participation, contributing to enhanced substrate utilisation and reductions in adiposity (Milanese et al., 2015), whereas the limited match involvement of the LP cohort mirrors findings in non‐starters, who often show higher fat levels (Hoppe et al., 2020). These findings support the view that greater competitive match exposure might contribute meaningfully to the overall metabolic stimulus experienced during late pre‐season.

### 4.2 Neuromuscular adaptation

Lower‐limb power is essential for high‐intensity actions in soccer. Although some studies report minimal changes in strength during the season (Saidi et al., 2019), our results showed significant neuromuscular gains in the EP cohort, with enhanced knee extension and flexion power post‐preparation. These improvements are likely to reflect repeated exposure to high mechanical loads and stretch–shortening cycle activity inherent to match‐play, consistent with evidence linking pre‐season play to improved power output (Caldwell & Peters, 2009; Hoppe et al., 2020). The LP group, with less exposure, showed limited gains. Importantly, no limb asymmetries were found, suggesting balanced neuromuscular adaptation that might reduce mechanical inefficiencies and susceptibility to injury (Croisier et al., 2008).

### 4.3 Anaerobic and change‐of‐direction adaptations

Pre‐season training typically enhances sprinting, agility and velocity metrics in soccer athletes (Caldwell & Peters, 2009; Dianzenza et al., 2009; Mylonis et al., 2025). In the present study, both cohorts improved to a similar extent in short sprint and change‐of‐direction performance; however, the EP group showed superior repeated sprint ability, suggesting enhanced metabolic resilience to repeated high‐intensity efforts, probably attributable to increased match‐play exposure and its associated anaerobic demands (Hostrup & Bangsbo, 2023). These findings suggest that greater exposure to repeated match‐play might contribute to the neuromuscular and metabolic stimuli associated with enhanced tolerance to intermittent high‐intensity loading.

### 4.4 Flexibility

Flexibility improved comparably in both groups, consistent with earlier findings (Caldwell & Peters, 2009), suggesting that match‐play had minimal differential effect. The similar response between groups is likely to reflect standardised flexibility training and the relatively low sensitivity of flexibility measures to variations in match exposure.

### 4.5 Aerobic capacity

Observed ${\dot{V}}_{O_{2}\max}$ and velocity values aligned with previously reported normative ranges for soccer players (Mylonis et al., 2025). Both cohorts showed improvements in ${\dot{V}}_{O_{2}\max}$ following pre‐season, confirming the efficacy of aerobic conditioning (Caldwell & Peters, 2009; Perroni et al., 2019; Śliwowski et al., 2013). Notably, the EP cohort achieved greater gains in $v{\dot{V}}_{O_{2}\max}$ and *v*VT_2_, indicating improved running economy and submaximal aerobic efficiency potentially associated with the greater cumulative match exposure, although the absence of individual internal and external load monitoring precludes direct attribution to a greater physiological match‐play load (Bekris et al., 2019) and concurrent reductions in body fat (Bekris et al., 2019; Silva et al., 2021). The parallel improvement in aerobic indices and repeated sprint ability suggests coordinated enhancement of oxygen transport and utilisation in intermittent high‐intensity conditions.

### 4.6 Biochemical and muscle stress markers

Glucose levels remained stable across both cohorts, indicating effective metabolic regulation, consistent with prior findings (Peres et al., 2022). Lipid profiles increased in a similar manner in both groups, probably owing to training‐induced lipid mobilisation and metabolic adaptations. However, the EP cohort showed elevated serum transaminases, reflecting increased muscle stress from higher match‐play exposure, as reported in prior training studies (Tiller & Stringer, 2023).

Sustained elevations in myoglobin and creatine phosphokinase in the EP group also support greater cumulative muscle damage, aligning with previous evidence linking match density to muscle stress (Celenk et al., 2013). In contrast, the LP cohort exhibited only transient increases in myoglobin, indicating lower muscular stress. Importantly, such elevations are likely to represent both structural muscle remodelling and transient tissue perturbation associated with repeated high‐intensity contractions.

### 4.7 Iron metabolism

Iron parameters fluctuated early in the LP cohort, probably reflecting transient redistribution, with both groups achieving comparable iron and ferritin levels by the end of the pre‐season. This suggests adequate haematological adaptation to training and match‐play. Although ferritin remained stable (indicating preserved iron stores), temporary reductions in circulating iron might reflect increased erythropoietic and metabolic demand during higher workloads (Noda et al., 2009). These findings underscore the dynamic regulation of iron availability during intensified training periods.

### 4.8 Perceptual and inflammatory responses to match‐play load

Although many studies rely on simulated protocols, real‐world match‐play imposes substantial neuromuscular and mechanical stress (Nagahara et al., 2016). In the present study, only the EP cohort exhibited a progressive increase in perceived exertion, suggesting cumulative strain, whereas stable ratings in the LP cohort are likely to reflect lower overall physiological load.

The inflammatory response, characterised by elevations in hs‐CRP, interleukin‐6 and TNF‐α, was transient in the LP group but sustained in the EP cohort, indicating a more pronounced systemic inflammatory activation with greater match exposure. This pattern is likely to reflect repeated muscle damage and biomechanical strain associated with frequent high‐intensity actions during match‐play (Kostrzewa‐Nowak & Nowak, 2020). Interleukin‐6 serves as a primary mediator of the acute phase response, driving subsequent increases in hs‐CRP and TNF‐α as part of tissue repair and immune activation (Jeong et al., 2011; Kostrzewa‐Nowak & Nowak, 2020; Mylonis et al., 2025; Sanchis‐Gomar et al., 2015).

Importantly, elevations in inflammatory biomarkers should not necessarily be interpreted as evidence of pathological inflammation. Exercise‐induced cytokine responses are recognised components of normal adaptive signalling, facilitating tissue remodelling, metabolic regulation and recovery following strenuous exercise. In the present study, inflammatory markers were generally within expected physiological ranges for trained individuals, and no evidence of clinical inflammation, illness, injury or performance deterioration was observed (Delongui et al., 2013; Kostrzewa‐Nowak & Nowak, 2020; Wyczalkowska‐Tomasik et al., 2016). Consequently, the observed elevations are more appropriately interpreted as indicators of increased physiological demand and adaptive activity rather than harmful inflammatory dysfunction. Nevertheless, the sustained elevation of inflammatory markers in the EP cohort relative to the LP cohort suggests that greater match exposure was associated with a higher cumulative inflammatory burden (Kostrzewa‐Nowak & Nowak, 2020). Although such responses might contribute to adaptive remodelling, they might also reflect increased requirements for recovery during periods of intensified loading. Accordingly, these findings highlight the importance of considering both the adaptive and physiological‐cost dimensions of repeated match exposure.

The magnitude and persistence of inflammatory markers in EP diverged from some prior reports (Mohr et al., 2016), possibly owing to differences in match density or recovery practices. Interindividual variability, including baseline fitness, genetics, nutrition, sleep and psychological stress, is also likely to modulate inflammatory dynamics (Andersson et al., 2010; Radom‐Aizik et al., 2009; Saidi et al., 2021).

### 4.9 Hormonal and haematological adaptations to match‐play load

Research on longitudinal hormonal dynamics in soccer remains limited (Bekris et al., 2022). Testosterone typically increases following preparatory phases (Bronkhorst et al., 2014), whereas cortisol might decline during stable competitive periods (Muscella et al., 2019; Mylonis et al., 2025; Perroni et al., 2019). In this study, the EP cohort exhibited elevated testosterone and testosterone‐to‐cortisol ratio during late pre‐season, consistent with an enhanced anabolic environment in response to repeated match loading (Vilamitjana et al., 2017). Such endocrine modulation might facilitate tissue repair and protein synthesis during periods of intensified mechanical stress (Pedroso et al., 2024; Saidi et al., 2021).

Haematological responses followed a distinct pattern (Filipovic et al., 2019; Saddam et al., 2017). The EP cohort displayed transient haemodilution followed by increases in erythrocytes and haemoglobin, suggesting adaptive erythropoiesis associated with elevated training and match demands (Filipovic et al., 2019; Saidi et al., 2021). Conversely, the LP cohort maintained relatively stable haematological profiles, consistent with lower cumulative physiological stress.

Leucocyte responses were variable. Although exercise‐induced leucocytosis is commonly reported (da Rocha et al., 2019), immune responses are influenced by workload, recovery status and external factors (Malm et al., 2004; Matonti et al., 2020; Nobari et al., 2021; Pedersen & Hoffman‐Goetz, 2000; Selmi et al., 2022). The more dynamic leucocyte profile in the EP cohort might reflect immune modulation secondary to repeated high‐intensity exposure.

### 4.10 Integrated physiological adaptation

Collectively, these findings suggest that greater match exposure within a common team‐training environment was associated with coordinated responses across metabolic, neuromuscular, endocrine, inflammatory and haematological systems. Although several between‐group effects were large, their interpretation should account for the number of outcomes examined, the exploratory nature of the analyses and the absence of formal adjustment for multiple comparisons. Nevertheless, the convergence of findings across related physiological domains provides a more informative basis for interpretation than any individual statistically significant result. Improvements in aerobic efficiency and muscle power occurred alongside elevations in anabolic hormones and erythrocyte indices, suggesting integrated enhancement of oxygen transport, energy production and mechanical output. However, these adaptive responses were paralleled by sustained inflammatory and muscle damage markers, consistent with greater physiological strain associated with repeated high‐intensity loading. This dual response underscores the dynamic balance between adaptive remodelling and cumulative stress during intensified match exposure.

### 4.11 Correlations

Increased match‐play has been associated with gains in sprint performance, agility, power and lean mass (Hoppe et al., 2020; Sporis et al., 2011), but also with elevated fatigue and injury risk (Jiang et al., 2022; Kraemer et al., 2004). Our findings indicate that greater friendly match exposure was associated with coordinated improvements in body composition, neuromuscular function and aerobic performance within the context of the overall pre‐season training programme. The strong associations with hs‐CRP and TNF‐α reinforce the close coupling between adaptive responses and systemic inflammatory activation. Correlations with iron and ferritin suggest that match‐play might influence iron redistribution, highlighting the need for monitoring during intensified training periods. Despite consistent group‐level trends, interindividual variability was evident, emphasising the importance of personalised load management to optimise adaptation while limiting excessive physiological strain.

### 4.12 Research/policy implications

The present findings indicate that extended friendly match exposure, beyond its tactical function, was associated with coordinated adaptations in body composition, neuromuscular performance and aerobic physiology. However, these adaptations were accompanied by sustained elevations in inflammatory and muscle damage markers, reflecting the systemic physiological cost of intensified match demands. Given that both starting and substitute players must reach comparable readiness prior to the competitive season, these observations highlight the need to consider match‐play not solely as a tactical preparation tool but as a substantial biological stimulus with multisystem consequences. Accordingly, periodisation strategies should integrate individualised exposure, structured recovery and regular monitoring of inflammatory, endocrine and haematological markers to balance adaptive remodelling with cumulative stress. An integrated, multidisciplinary framework, incorporating coaching, sports medicine, physiology and performance science, might optimise adaptive outcomes while supporting recovery from the greater physiological demands associated with increased match exposure. These insights can inform evidence‐based strategies aimed at supporting sustainable performance development and athlete health in semi‐professional soccer environments.

### 4.13 Limitations

Several limitations warrant consideration. Match exposure was quantified using total playing time, which does not capture the external or internal load experienced during matches. The absence of objective tracking metrics (e.g., GPS‐derived total and high‐speed running distance, acceleration–deceleration profiles and other locomotor‐load indices) or video‐based movement analysis might have limited the precision of individual match‐load quantification. Moreover, individual internal responses to both training and match‐play were not monitored systematically using measures such as session‐rating of perceived exertion, heart rate or other validated internal‐load indices. Consequently, players with similar playing time might have experienced substantially different physiological and mechanical loads, and the differences in overall training exposure between players could not be quantified. This limitation is particularly relevant to the interpretation of differences between the EP and LP cohorts because cumulative playing time was used as a proxy for match exposure rather than as a direct measure of physiological workload. Thus, the observed between‐cohort differences cannot be attributed exclusively to differences in the physiological load imposed by match‐play.

Although all participants followed the same team training programme (∼12 h week^−1^), the individual training load generated by this programme was not quantified. Consequently, potential between‐player differences in internal load responses (e.g., perceived exertion, cardiovascular strain or recovery status) could not be accounted for. Furthermore, positional demands, tactical roles and the timing and duration of match participation might have varied between players despite a common training environment. Because different playing positions are characterised by distinct locomotor, metabolic and technical demands, variation in positional exposure might have contributed to differences in physiological adaptation. Given that these factors were not quantified, their potential contribution to individual physiological responses cannot be determined.

Uncontrolled factors, including individual resistance training exposure, psychological stress, sleep quality and dietary intake, might have contributed to interindividual variability in adaptive and inflammatory responses. Importantly, players were classified retrospectively according to match exposure that arose from coaching decisions rather than investigator‐controlled allocation. Thus, the EP and LP cohorts were not assigned randomly, and the possibility of selection bias cannot be excluded. Players receiving greater match exposure might have differed systematically from those receiving limited exposure in pre‐existing characteristics that influenced coaching decisions, such as fitness, playing performance, tactical role, positional demands, experience or perceived readiness. Although participants were drawn from the same team, followed the same training programme and competed within the same competitive environment, these characteristics were not controlled experimentally and might therefore have contributed to the observed between‐cohort differences. Accordingly, the findings should be interpreted as exposure‐dependent associations within a natural experiment rather than evidence of direct causal effects of match‐play exposure. Given the number of statistical tests performed across the study outcomes, some inflation of the overall type I error rate cannot be excluded. Although Bonferroni corrections were applied to the relevant pairwise comparisons, the results should therefore be interpreted with due consideration of the broader multiplicity of the analyses. For this reason, interpretation of the findings was guided not only by statistical significance but also by the magnitude and precision of the estimated effects, including their corresponding confidence intervals. The relatively large $\eta_{p}^{2}$ estimates observed for several outcomes should likewise be interpreted in conjunction with their confidence intervals, *P*‐values, biological coherence and the exploratory nature of the analyses, rather than being considered definitive evidence of the magnitude or causal importance of match exposure. Emphasis was therefore placed on convergent patterns across related physiological domains rather than isolated statistical findings.

All physiological assessments were conducted within a single testing session, which might have introduced residual fatigue effects despite standardised recovery procedures. The study cohort comprised a single male semi‐professional team, thereby restricting generalisability to other competitive levels or age groups or to female athletes.

Future investigations should incorporate individualised monitoring of internal and external training and match loads, including session‐rating of perceived exertion, heart rate‐derived indices, GPS metrics and movement analysis, to distinguish playing‐time exposure from the physiological and mechanical load experienced by each player, expand samples across sex and competitive levels, and include longitudinal assessment of behavioural and recovery‐related variables to characterise the interaction between mechanical load, systemic adaptation and inflammatory regulation.

## 5 CONCLUSION

Greater friendly match exposure during late pre‐season was associated with coordinated improvements in neuromuscular function, aerobic efficiency and body composition, supporting the concept that friendly match‐play acts not only as a tactical preparation tool but also as a meaningful physiological stimulus. The observed associations between match exposure, anabolic signalling, erythropoietic indices and inflammatory mediators underscore the tightly coupled nature of adaptive remodelling and physiological stress during intensified training periods. These findings also support the concept that greater friendly match exposure is associated with coordinated responses across multiple physiological systems during pre‐season preparation. However, the accompanying inflammatory and muscle‐stress responses highlight the importance of carefully balancing match exposure with structured recovery to optimise adaptation while appropriately managing cumulative physiological demands and recovery requirements. Collectively, this study provides a systems‐level perspective on late pre‐season match‐play, emphasising the need for physiologically informed load management to promote sustainable performance development in competitive soccer.

## AUTHOR CONTRIBUTIONS

Dimitrios I. Bourdas: Conception and design: Conceptualization, Methodology, Visualization, Writing—Original Draft Preparation, Writing—Review and Editing; Eleftherios Mylonis: Methodology, Data Curation, Funding Acquisition, Investigation, Validation, Formal Analysis, Resources, Writing—Review and Editing, Supervision, Project Administration; Natalia KompodietaMethodology, Data Curation, Funding Acquisition, Investigation, Validation, Formal Analysis, Resources, Writing—Review and Editing, Supervision, Project Administration; Panteleimon Bakirtzoglou: Data Curation, Funding Acquisition, Investigation, Resources, Writing—Review and Editing; Athanasios Souglis: Data Curation, Funding Acquisition, Investigation, Resources, Writing—Review and Editing; Evangelos Bekris: Conceptualization, Data Curation, Funding Acquisition, Resources, Writing—Review and Editing; Antonios K. Travlos: Conceptualization, Data Curation, Funding Acquisition, Resources, Writing—Review and Editing; Apostolos Theos: Data Curation, Funding Acquisition, Investigation, Resources, Writing—Review and Editing. All authors had full access to all the data from this study. All authors were responsible for the decision to submit the manuscript for publication. All authors have read and agreed to the published version of the manuscript and agree to be accountable for all aspects of the work in ensuring that questions related to the accuracy or integrity of any part of the work are appropriately investigated and resolved. All persons designated as authors qualify for authorship, and all those who qualify for authorship are listed.

## CONFLICT OF INTEREST

The authors declare no conflict of interest with respect to the research, authorship and/or publication of this article.

## FUNDING INFORMATION

No financial support was received for the conduct of this study or preparation of this manuscript.

## GENERATIVE AI STATEMENT

No generative AI tools were used in the preparation of this manuscript.

## Supporting information



Supporting Information

**Supporting Information**: eph70509‐sup‐0002‐SuppMat.pdf

## Data Availability

The datasets generated and analysed during the present study are publicly available in the Zenodo repository (https://doi.org/10.5281/zenodo.18851331). The dataset comprises anonymised anthropometric, performance, biochemical, hormonal and haematological variables obtained at pre‐season start, mid‐pre‐season and pre‐competition time points.

## 1

**TABLE A1:** Physical, biochemical, hormonal, haematological and performance data of the EP cohort (*n* = 12) for PS, MPS and PC conditions, presented as the mean ± SD [95% CI]

Variable	PS	MPS	PC
Body mass, kg^‡^, ^§^	76.71 ± 5.01 [73.88–79.54]	76.15 ± 5.53 [73.02–79.28]	74.74 ± 5.82 [71.45–78.03]
Body fat, %^†^, ^‡^, ^§^	12.49 ± 1.81 [11.47–13.51]	10.63 ± 1.84 [9.59–11.67]	9.25 ± 1.86 [8.20–10.30]
Rating of perceived exertion, Borg scale (1–10)^†^, ^‡^	4.92 ± 0.9 [4.41–5.43]	7.42 ± 0.51 [7.13–7.71]	7.50 ± 0.52 [7.21–7.79]
Glucose, mg dL^−^^1^	91.00 ± 10.43 [85.10–96.90]	82.50 ± 7.68 [78.15–86.85]	89.08 ± 5.90 [85.74–92.42]
Cholesterol, mg dL^−^^1^ ^‡^, ^§^	154.50 ± 25.50 [140.07–168.93]	160.33 ± 27.50 [144.77–175.89]	170.83 ± 29.97 [153.87–187.79]
Τriglycerides, mg dL^−^^1^ ^§^	86.67 ± 34.92 [66.91–106.43]	74.17 ± 20.14 [62.77–85.57]	86.17 ± 20.28 [74.70–97.64]
Serum glutamic–oxaloacetic transaminase, U L^−^^1^ ^‡^, ^§^	21.67 ± 5.68 [18.46–24.88]	25.25 ± 4.27 [22.83–27.67]	29.50 ± 7.14 [25.46–33.54]
Serum glutamic–pyruvic transaminase, U L^−^^1^ ^‡^	19.00 ± 8.43 [14.23–23.77]	23.67 ± 5.97 [20.29–27.05]	26.42 ± 8.36 [21.69–31.15]
Creatine phosphokinase, U L^−^^1^ ^‡^	136.83 ± 63.38 [100.97–172.69]	208.00 ± 74.32 [165.95–250.05]	232.25 ± 86.8 [183.14–281.36]
Myoglobin, mg L^−^^1^ ^†^, ^‡^	37.83 ± 16.79 [28.33–47.33]	72.42 ± 31.53 [54.58–90.26]	83.33 ± 50.44 [54.79–111.87]
Iron, µg dL^−^^1^	82.25 ± 40.01 [59.61–104.89]	73.67 ± 12.92 [66.36–80.98]	79.58 ± 15.22 [70.97–88.19]
Ferritin, mg L^−^^1^	93.28 ± 48.49 [65.84–120.72]	78.70 ± 26.77 [63.55–93.85]	74.09 ± 26.12 [59.31–88.87]
C‐Reactive protein, mg L^−^^1^	0.30 ± 0.06 [0.27–0.33]	0.39 ± 0.11 [0.33–0.45]	0.36 ± 0.11 [0.30–0.42]
High‐sensitivity C‐reactive protein, mg L^−^^1^ ^†^, ^‡^, ^§^	0.89 ± 0.23 [0.76–1.02]	1.62 ± 0.27 [1.47–1.77]	2.28 ± 0.28 [2.12–2.44]
Interleukin‐6, mg L^−^^1^ ^†^, ^‡^, ^§^	8.50 ± 1.83 [7.46–9.54]	9.63 ± 1.94 [8.53–10.73]	13.79 ± 1.44 [12.98–14.60]
Tumour necrosis factor‐α, pg mL^−^^1^ ^†^, ^‡^, ^§^	5.71 ± 0.66 [5.34–6.08]	7.95 ± 1.17 [7.29–8.61]	11.58 ± 0.68 [11.20–11.96]
Testosterone, ng mL^−^^1^ ^‡^, ^§^	4.73 ± 0.60 [4.39–5.07]	5.41 ± 1.36 [4.64–6.18]	6.66 ± 1.69 [5.70–7.62]
Cortisol, µg dL^−^^1^	14.28 ± 4.63 [11.66–16.90]	18.05 ± 1.99 [16.92–19.18]	16.23 ± 3.71 [14.13–18.33]
Testosterone:cortisol ratio (T:C) ^§^	0.37 ± 0.13 [0.30–0.44]	0.30 ± 0.08 [0.25–0.35]	0.42 ± 0.11 [0.36–0.48]
Red blood cell count, ×10^1^ ^2^ L^−^^1^ ^†^, ^§^	5.35 ± 0.60 [5.01–5.69]	5.04 ± 0.47 [4.77–5.31]	5.63 ± 0.57 [5.31–5.95]
Haemoglobin, g dL^−^^1^ ^†^, ^‡^	15.50 ± 1.44 [14.69–16.31]	14.25 ± 1.58 [13.36–15.14]	14.64 ± 1.53 [13.77–15.51]
Haematocrit, %^†^	46.18 ± 3.88 [43.98–48.38]	43.19 ± 3.95 [40.96–45.42]	44.33 ± 4.01 [42.06–46.60]
White blood cell count, ×10^9^ L^−^^1^ ^†^	7.03 ± 1.53 [6.16–7.90]	6.12 ± 0.99 [5.56–6.68]	6.46 ± 1.25 [5.75–7.17]
Platelet count, ×10^9^ L^−^^1^	227.58 ± 34.14 [208.26–246.90]	206.65 ± 73.35 [165.15–248.15]	219.08 ± 46.50 [192.77–245.39]
Countermovement jump height, cm^‡^, ^§^	39.59 ± 2.63 [38.10–41.08]	40.59 ± 2.37 [39.25–41.93]	42.11 ± 2.57 [40.66–43.56]
Power of knee extensors, dominant leg, W^‡^, ^§^	198.17 ± 16.02 [189.11–207.23]	208.83 ± 18.44 [198.40–219.26]	220.33 ± 17.22 [210.59–230.07]
Power of knee extensors, non‐dominant leg, W^‡^, ^§^	201.00 ± 18.76 [190.39–211.61]	207.83 ± 18.70 [197.25–218.41]	221.17 ± 18.01 [210.98–231.36]
Power of knee flexors, dominant leg, W^‡^, ^§^	143.50 ± 23.77 [130.05–156.95]	145.42 ± 9.80 [139.88–150.96]	160.25 ± 10.38 [154.38–166.12]
Power of knee flexors, non‐dominant leg, W^‡^, ^§^	137.83 ± 20.67 [126.14–149.52]	141.42 ± 20.14 [130.02–152.82]	150.92 ± 18.93 [140.21–161.63]
10 m sprint time, s^†^, ^‡^	1.82 ± 0.05 [1.79–1.85]	1.71 ± 0.04 [1.69–1.73]	1.71 ± 0.03 [1.69–1.73]
CODS, s^†^, ^‡^, ^§^	16.50 ± 0.32 [16.32–16.68]	16.25 ± 0.30 [16.08–16.42]	15.73 ± 0.36 [15.53–15.93]
Average 35 m running time during RAST, s^†^, ^‡^, ^§^	5.17 ± 0.15 [5.09–5.25]	5.07 ± 0.13 [5.00–5.14]	4.83 ± 0.16 [4.74–4.92]
Speed drop during RAST, %^†^	9.90 ± 20 [8.77–11.03]	7.36 ± 1.10 [6.74–7.98]	9.39 ± 3.41 [7.46–11.32]
Hamstring and lower back flexibility, cm	23.58 ± 6.32 [20.00–27.16]	24.42 ± 6.71 [20.62–28.22]	26.00 ± 4.79 [23.29–28.71]
${\dot{V}}_{O_{2}\max}$, mL kg^−^^1^ min^−^^1^ ^†^, ^‡^, ^§^	51.89 ± 4.21 [49.51–54.27]	55.57 ± 4.48 [53.04–58.10]	57.37 ± 3.94 [55.14–59.60]
$v{\dot{V}}_{O_{2}\max}$, km h^−^^1^ ^†^, ^‡^, ^§^	15.96 ± 0.86 [15.47–16.45]	16.63 ± 0.88 [16.13–17.13]	17.63 ± 0.96 [17.09–18.17]
*v*VT_2_, km h^−^^1^ ^†^, ^‡^, ^§^	12.08 ± 0.79 [11.63–12.53]	12.83 ± 0.65 [12.46–13.20]	13.83 ± 0.62 [13.48–14.18]
HR_max_ at ${\dot{V}}_{O_{2}\max}$, beats min^−^^1^ ^†^	194.33 ± 7.38 [190.15–198.51]	189.33 ± 7.14 [185.29–193.37]	190.67 ± 8.87 [185.65–195.69]
HR at VT_2_, beats min^−^^1^ ^‡^	166.67 ± 5.73 [163.43–169.91]	163.00 ± 6.27 [159.45–166.55]	162.42 ± 5.42 [159.35–165.49]
Lactate at ${\dot{V}}_{O_{2}\max}$, mmol l^−^^1^ ^†^, ^‡^	13.01 ± 2.08 [11.83–14.19]	10.77 ± 1.45 [9.95–11.59]	10.84 ± 1.51 [9.99–11.69]

Abbreviations: CI, confidence interval; CODS, change of direction speed; EP, extended play in friendly games during the late pre‐season preparation phase; HR, heart rate; HR_max_, maximal heart rate; MPS, middle of the pre‐season preparation phase; *n*, sample size; PC, 1 week prior to the initiation of the competition phase; PS, prior to the initiation of the 9 weeks of pre‐season preparation phase; RAST, running‐based anaerobic sprint test; ${\dot{V}}_{O_{2}\max}$, maximal oxygen uptake; VT_2_, second ventilatory threshold; $v{\dot{V}}_{O_{2}\max}$, velocity at ${\dot{V}}_{O_{2}\max}$; *v*VT_2_, velocity at VT_2_.

^†^ Significant difference between PS and MPS conditions at *P* < 0.05.

^‡^ Significant difference between PS and PC conditions at *P* < 0.05.

^§^ Significant difference between MPS and PC conditions at *P* < 0.05.

**TABLE A2:** Physical, biochemical, hormonal, haematological and performance data of the LP cohort (*n* = 12) for PS, MPS and PC conditions, presented as the mean ± SD [95% CI]

Variable	PS	MPS	PC
Body mass, kg	72.24 ± 8.19 [67.61–76.87]	71.93 ± 8.24 [67.27–76.59]	72.80 ± 7.38 [68.62–76.98]
Body fat, %	12.32 ± 3.31 [10.45–14.19]	11.79 ± 2.98 [10.10–13.48]	11.33 ± 2.17 [10.10–12.56]
Rating of perceived exertion, Borg scale (1–10)^†^, ^‡^	5.33 ± 0.65 [4.96–5.70]	7.50 ± 0.80 [7.05–7.95]	7.50 ± 0.80 [7.05–7.95]
Glucose, mg dL^−^^1^	87.17 ± 13.55 [79.50–94.84]	83.58 ± 9.31 [78.31–88.85]	84.08 ± 6.73 [80.27–87.89]
Cholesterol, mg dL^−^^1^ ^§^	159.42 ± 33.12 [140.68–178.16]	154.42 ± 25.44 [140.03–168.81]	169.58 ± 23.28 [156.41–182.75]
Τriglycerides, mg dL^−^^1^ ^§^	97.50 ± 33.88 [78.33–116.67]	71.58 ± 20.83 [59.79–83.37]	90.50 ± 23.49 [77.21–103.79]
Serum glutamic–oxaloacetic transaminase, U L^−^^1^	29.17 ± 14.92 [20.73–37.61]	27.33 ± 7.18 [23.27–31.39]	26.50 ± 7.96 [22.00–31.00]
Serum glutamic–pyruvic transaminase, U L^−^^1^	23.17 ± 8.00 [18.64–27.70]	24.25 ± 5.31 [21.25–27.25]	25.67 ± 7.88 [21.21–30.13]
Creatine phosphokinase, U L^−^^1^	238.25 ± 90.88 [186.83–289.67]	309.75 ± 106.98 [249.22–370.28]	275.08 ± 150.93 [189.68–360.48]
Myoglobin, mg L^−^^1^ ^†^, ^§^	71.51 ± 31.27 [53.82–89.20]	111.00 ± 32.35 [92.7–129.30]	81.33 ± 31.36 [63.59–99.07]
Iron, µg dL^−^^1^ ^†^	117.67 ± 30.47 [100.43–134.91]	92.25 ± 32.31 [73.97–110.53]	104.50 ± 32.58 [86.07–122.93]
Ferritin, mg L^−^^1^	51.43 ± 26.26 [36.57–66.29]	48.64 ± 21.67 [36.38–60.90]	50.56 ± 22.0 [38.11–63.01]
C‐Reactive protein, mg L^−^^1^	0.72 ± 0.90 [0.21–1.23]	0.31 ± 0.05 [0.28–0.34]	0.32 ± 0.07 [0.28–0.36]
High‐sensitivity C‐reactive protein, mg L^−^^1^ ^†^, ^§^	0.77 ± 0.21 [0.65–0.89]	1.80 ± 0.27 [1.65–1.95]	0.80 ± 0.16 [0.71–0.89]
Interleukin‐6, mg L^−^^1^ ^†^, ^§^	6.73 ± 2.25 [5.46–8.00]	7.62 ± 2.72 [6.08–9.16]	6.00 ± 1.85 [4.95–7.05]
Tumour necrosis factor‐α, pg mL^−^^1^ ^†^, ^§^	6.38 ± 0.96 [5.84–6.92]	8.62 ± 0.71 [8.22–9.02]	6.02 ± 0.74 [5.60–6.44]
Testosterone, ng mL^−^^1^	5.80 ± 0.91 [5.29–6.31]	5.53 ± 1.14 [4.88–6.18]	6.02 ± 1.46 [5.19–6.85]
Cortisol, µg dL^−^^1^	16.66 ± 3.08 [14.92–18.40]	17.81 ± 4.06 [15.51–20.11]	16.42 ± 3.72 [14.32–18.52]
Testosterone:cortisol ratio (T:C)	0.36 ± 0.09 [0.31–0.41]	0.32 ± 0.06 [0.29–0.35]	0.38 ± 0.10 [0.32–0.44]
Red blood cell count, ×10^1^ ^2^ L^−^^1^	5.35 ± 0.62 [5.00–5.70]	5.29 ± 0.65 [4.92–5.66]	5.49 ± 0.74 [5.07–5.91]
Haemoglobin, g dL^−^^1^	15.18 ± 1.51 [14.33–16.03]	14.72 ± 1.02 [14.14–15.3]	14.91 ± 0.82 [14.45–15.37]
Haematocrit, %	45.39 ± 2.83 [43.79–46.99]	44.86 ± 2.24 [43.59–46.13]	45.14 ± 2.98 [43.45–46.83]
White blood cell count, ×10^9^ L^−^^1^	6.39 ± 1.56 [5.51–7.27]	6.40 ± 1.70 [5.44–7.36]	6.34 ± 1.86 [5.29–7.39]
Platelet count, ×10^9^ L^−^^1^	229.67 ± 55.61 [198.21–261.13]	219.67 ± 52.70 [189.85–249.49]	206.25 ± 53.11 [176.20–236.30]
Countermovement jump height, cm^‡^, ^§^	39.18 ± 5.43 [36.11–42.25]	39.68 ± 6.24 [36.15–43.21]	41.91 ± 5.19 [38.97–44.85]
Power of knee extensors, dominant leg, W^†^, ^‡^	175.08 ± 20.81 [163.31–186.85]	191.83 ± 21.87 [179.46–204.20]	192.50 ± 24.07 [178.88–206.12]
Power of knee extensors, non‐dominant leg, W^†^, ^‡^	181.75 ± 20.96 [169.89–193.61]	200.00 ± 21.80 [187.67–212.33]	196.33 ± 24.03 [182.73–209.93]
Power of knee flexors, dominant leg, W	123.00 ± 22.32 [110.37–135.63]	132.25 ± 14.67 [123.95–140.55]	131.75 ± 17.92 [121.61–141.89]
Power of knee flexors, non‐dominant leg, W	110.42 ± 18.37 [100.03–120.81]	124.83 ± 19.95 [113.54–136.12]	125.75 ± 17.82 [115.67–135.83]
10 m sprint time, s^†^, ^‡^	1.77 ± 0.07 [1.73–1.81]	1.69 ± 0.06 [1.66–1.72]	1.67 ± 0.07 [1.63–1.71]
CODS, s^‡^, ^§^	16.54 ± 0.6 [16.20–16.88]	16.43 ± 0.59 [16.10–16.76]	16.07 ± 0.67 [15.69–16.45]
Average 35 m running time during RAST, s^†^	5.13 ± 0.17 [5.03–5.23]	5.04 ± 0.17 [4.94–5.14]	5.14 ± 0.31 [4.96–5.32]
Speed drop during RAST, %^†^	9.29 ± 3.31 [7.42–11.16]	6.06 ± 2.94 [4.40–7.72]	9.39 ± 2.76 [7.83–10.95]
Hamstring and lower back flexibility, cm^†^, ^‡^	21.17 ± 4.84 [18.43–23.91]	23.08 ± 4.66 [20.44–25.72]	24.33 ± 4.91 [21.55–27.11]
${\dot{V}}_{O_{2}\max}$, mL kg^−^^1^ min^−^^1^ ^‡^, ^§^	54.06 ± 4.37 [51.59–56.53]	55.68 ± 4.43 [53.17–58.19]	57.25 ± 3.59 [55.22–59.28]
$v{\dot{V}}_{O_{2}\max}$, km h^−^^1^ ^†^	16.00 ± 0.83 [15.53–16.47]	16.54 ± 0.86 [16.05–17.03]	16.29 ± 1.03 [15.71–16.87]
*v*VT_2_, km h^−^^1^ ^†^	12.13 ± 0.68 [11.75–12.51]	12.67 ± 0.78 [12.23–13.11]	12.35 ± 0.61 [12.00–12.70]
HR_max_ at ${\dot{V}}_{O_{2}\max}$, beats min^−^^1^	198.17 ± 7.91 [193.69–202.65]	195.33 ± 8.77 [190.37–200.29]	194.67 ± 9.32 [189.40–199.94]
HR at VT_2_, beats min^−^^1^	169.58 ± 9.08 [164.44–174.72]	168.5 ± 8.03 [163.96–173.04]	167.67 ± 10.11 [161.95–173.39]
Lactate at ${\dot{V}}_{O_{2}\max}$, mmol L^−^^1^ ^‡^	11.23 ± 0.65 [10.86–11.60]	11.58 ± 1.53 [10.71–12.45]	12.67 ± 1.06 [12.07–13.27]

Abbreviations: CI, confidence interval; CODS, change of direction speed; HR, heart rate; HR_max_, maximal heart rate; LP, limited play in friendly games during the late pre‐season preparation phase; MPS, middle of the pre‐season preparation phase; *n*, sample size; PC, 1 week prior to the initiation of the competition phase; PS, prior to the initiation of the 9 weeks of pre‐season preparation phase; RAST, running‐based anaerobic sprint test; ${\dot{V}}_{O_{2}\max}$, maximal oxygen uptake; VT_2_, second ventilatory threshold; $v{\dot{V}}_{O_{2}\max}$, velocity at ${\dot{V}}_{O_{2}\max}$; *v*VT_2_, velocity at VT_2_.

^†^ Significant difference between PS and MPS conditions at *P* < 0.05.

^‡^ Significant difference between PS and PC conditions at *P* < 0.05.

^§^ Significant difference between MPS and PC conditions at *P* < 0.05.

**TABLE A3:** Bonferroni‐adjusted *P*‐values for pairwise comparisons between conditions (PS vs. MPS, PS vs. PC and MPS vs. PC) across all variables of the EP cohort (*n* = 12)

Variable	PS vs. MPS	PS vs. PC	MPS vs. PC
Body mass^‡^, ^§^	0.991	0.026	0.047
Body fat^†^, ^‡^, ^§^	<0.001	<0.001	0.001
Rating of perceived exertion^†^, ^‡^	<0.001	<0.001	>0.999
Glucose	0.248	>0.999	0.056
Cholesterol^‡^, ^§^	0.465	0.025	0.009
Τriglycerides^§^	0.489	>0.999	0.039
Serum glutamic–oxaloacetic transaminase^‡^, ^§^	0.090	0.006	0.023
Serum glutamic–pyruvic transaminase^‡^	0.173	0.019	0.176
Creatine phosphokinase^‡^	0.088	0.017	0.679
Myoglobin^†^, ^‡^	<0.001	0.011	0.927
Iron	>0.999	>0.999	>0.999
Ferritin	0.459	0.296	>0.999
C‐Reactive protein	0.082	0.427	0.831
High‐sensitivity C‐reactive protein^†^, ^‡^, ^§^	<0.001	<0.001	<0.001
Interleukin‐6^†^, ^‡^, ^§^	0.035	<0.001	<0.001
Tumour necrosis factor α^†^, ^‡^, ^§^	<0.001	<0.001	<0.001
Testosterone^‡^, ^§^	0.128	0.006	0.026
Cortisol	0.075	0.226	0.414
Testosterone:cortisol ratio^§^	0.768	0.131	0.014
Red blood cell count^†^, ^§^	0.006	0.221	0.001
Haemoglobin^†^, ^‡^	0.008	0.017	0.509
Haematocrit^†^	0.006	0.199	0.458
White blood cell count^†^	0.048	0.517	0.441
Platelet count	>0.999	>0.999	>0.999
Countermovement jump height^‡^, ^§^	0.478	0.019	0.006
Power of knee extensors, dominant leg^‡^, ^§^	0.130	0.010	0.019
Power of knee extensors, non‐dominant leg^‡^, ^§^	0.409	0.010	0.002
Power of knee flexors, dominant leg^‡^, ^§^	>0.999	0.036	0.007
Power of knee flexors, non‐dominant leg^‡^, ^§^	0.613	<0.001	0.004
10 m sprint time^†^, ^‡^	<0.001	0.001	>0.999
Agility^†^, ^‡^, ^§^	0.002	<0.001	<0.001
Average 35 m running time during RAST^†^, ^‡^, ^§^	<0.001	<0.001	0.001
Speed drop during RAST^†^	0.019	>0.999	0.232
Hamstring and lower back flexibility	>0.999	0.061	0.437
${\dot{V}}_{O_{2}\max}$ ^†^, ^‡^, ^§^	<0.001	<0.001	0.039
$v{\dot{V}}_{O_{2}\max}$ ^†^, ^‡^, ^§^	0.038	<0.001	0.004
*v*VT_2_ ^†^, ^‡^, ^§^	0.002	<0.001	<0.001
HR_max_ at ${\dot{V}}_{O_{2}\max}$ ^†^	0.002	0.072	0.765
HR at VT_2_ ^‡^	0.057	0.019	>0.999
Lactate at ${\dot{V}}_{O_{2}\max}$ ^†^, ^‡^	0.040	0.048	>0.999

Abbreviations: EP, extended play in friendly games during the late pre‐season preparation phase; HR, heart rate; HR_max_, maximal heart rate; MPS, middle of the pre‐season preparation phase; *n*, sample size; PC, 1 week prior to the initiation of the competition phase; PS, prior to the initiation of the 9 weeks of pre‐season preparation phase; RAST, running‐based anaerobic sprint test; ${\dot{V}}_{O_{2}\max}$, maximal oxygen uptake; VT_2_, second ventilatory threshold; $v{\dot{V}}_{O_{2}\max}$, velocity at ${\dot{V}}_{O_{2}\max}$; *v*VT_2_, velocity at VT_2_.

^†^ Significant difference between PS and MPS conditions at *P* ≤ 0.05.

^‡^ Significant difference between PS and PC conditions at *P* ≤ 0.05.

^§^ Significant difference between MPS and PC conditions at *P* ≤ 0.05.

**TABLE A4:** Bonferroni‐adjusted *P*‐values for pairwise comparisons between conditions (PS vs. MPS, PS vs. PC and MPS vs. PC) across all variables of the LP cohort (*n* = 12)

Variable	PS vs. MPS	PS vs. PC	MPS vs. PC
Body mass	>0.999	>0.999	0.268
Body fat	0.372	0.188	0.951
Rating of perceived exertion^†^, ^‡^	<0.001	<0.001	>0.999
Glucose	>0.999	>0.999	>0.999
Cholesterol^§^	>0.999	0.447	0.018
Τriglycerides^§^	0.070	>0.999	0.046
Serum glutamic–oxaloacetic transaminase	>0.999	>0.999	>0.999
Serum glutamic–pyruvic transaminase	>0.999	>0.999	>0.999
Creatine phosphokinase	0.125	>0.999	0.631
Myoglobin^†^, ^§^	0.015	0.825	0.010
Iron^†^	0.042	0.305	0.165
Ferritin	0.839	>0.999	>0.999
C‐Reactive protein	0.418	0.466	>0.999
High‐sensitivity C‐reactive protein^†^, ^§^	<0.001	>0.999	<0.001
Interleukin‐6^†^, ^§^	0.036	0.446	0.049
Tumour necrosis factor‐α^†^, ^§^	<0.001	>0.999	<0.001
Testosterone	0.987	>0.999	>0.999
Cortisol	>0.999	>0.999	>0.999
Testosterone:cortisol ratio	>0.999	>0.999	0.074
Red blood cell count	>0.999	>0.999	0.329
Haemoglobin	0.305	>0.999	>0.999
Haematocrit	0.836	>0.999	>0.999
White blood cell count	>0.999	>0.999	>0.999
Platelet count	0.332	0.060	0.266
Countermovement jump height^‡^, ^§^	>0.999	<0.001	0.010
Power of knee extensors, dominant leg^†^, ^‡^	0.025	0.025	>0.999
Power of knee extensors, non‐dominant leg^†^, ^‡^	0.010	0.021	0.860
Power of knee flexors, dominant leg	0.708	0.488	>0.999
Power of knee flexors, non‐dominant leg	0.088	0.053	>0.999
10 m sprint time^†^, ^‡^	0.001	0.008	0.976
Agility^‡^, ^§^	0.578	0.003	0.018
Average 35 m running time during RAST^†^	0.003	>0.999	0.391
Speed drop during RAST^†^	0.036	>0.999	0.087
Hamstring and lower back flexibility^†^, ^‡^	<0.001	0.001	0.425
${\dot{V}}_{O_{2}\max}$ ^‡^, ^§^	0.076	0.001	0.002
$v{\dot{V}}_{O_{2}\max}$ ^†^	0.009	0.394	0.825
*v*VT_2_ ^†^	0.035	0.387	0.441
HR_max_ at ${\dot{V}}_{O_{2}\max}$	0.130	0.134	>0.999
HR at VT_2_	>0.999	>0.999	>0.999
Lactate at ${\dot{V}}_{O_{2}\max}$ ^‡^	>0.999	0.017	0.164

Abbreviations: HR, heart rate; HR_max_, maximal heart rate; LP, limited play in friendly games during the late pre‐season preparation phase; MPS, middle of the pre‐season preparation phase; *n*, sample size; PC, 1 week prior to the initiation of the competition phase; PS, prior to the initiation of the 9 weeks of pre‐season preparation phase; RAST, running‐based anaerobic sprint test; ${\dot{V}}_{O_{2}\max}$, maximal oxygen uptake; VT_2_, second ventilatory threshold; $v{\dot{V}}_{O_{2}\max}$, velocity at ${\dot{V}}_{O_{2}\max}$; *v*VT_2_, velocity at VT_2_.

^†^ Significant difference between PS and MPS conditions at *P* ≤ 0.05.

^‡^ Significant difference between PS and PC conditions at *P* ≤ 0.05.

^§^ Significant difference between MPS and PC conditions at *P* ≤ 0.05.

**TABLE A5:** Cohen's *d* effect sizes and 95% confidence intervals, enclosed in square brackets, for pairwise comparisons between conditions (PS vs. MPS, PS vs. PC and MPS vs. PC) across all variables of the EP cohort (*n* = 12)

Variable	PS vs. MPS	PS vs. PC	MPS vs. PC
Body mass^‡^, ^§^	−0.11 [−0.68–0.46]	−0.36 [−0.95–0.23]	−0.25 [−0.82–0.32]
Body fat^†^, ^‡^, ^§^	−1.02 [−1.73 to −0.31]	−1.76 [−2.66 to −0.86]	−0.75 [−1.40 to −0.10]
Rating of perceived exertion^†^, ^‡^	3.21 [1.80–4.62]	3.31 [1.88–4.74]	0.15 [−0.42 to 0.72]
Glucose	−0.91 [−1.58 to −0.24]	−0.21 [−0.78 to 0.36]	0.95 [0.26–1.64]
Cholesterol^‡^, ^§^	0.22 [−0.35 to 0.79]	0.58 [−0.03 to 1.19]	0.36 [−0.23 to 0.95]
Τriglycerides^§^	−0.41 [−1.00 to 0.18]	−0.02 [−0.59 to 0.55]	0.59 [−0.02 to 1.20]
Serum glutamic–oxaloacetic transaminase^‡^, ^§^	0.70 [0.07–1.33]	1.20 [0.46–1.94]	0.68 [0.05–1.31]
Serum glutamic–pyruvic transaminase^‡^	0.62 [−0.01 to 1.25]	0.88 [0.21–1.55]	0.37 [−0.22 to 0.96]
Creatine phosphokinase^‡^	1.02 [0.31–1.73]	1.23 [0.49–1.97]	0.30 [−0.29 to 0.89]
Myoglobin^†^, ^‡^	1.27 [0.51–2.03]	1.02 [0.31–1.73]	0.25 [−0.32 to 0.82]
Iron	−0.24 [−0.81 to 0.33]	−0.08 [−0.65 to 0.49]	0.42 [−0.17 to 1.01]
Ferritin	−0.35 [−0.94 to 0.24]	−0.46 [−1.05 to 0.13]	−0.17 [−0.74 to 0.40]
C‐Reactive protein	0.90 [0.23–1.57]	0.60 [−0.01 to 1.21]	−0.27 [−0.84 to 0.30]
High‐sensitivity C‐reactive protein^†^, ^‡^, ^§^	2.92 [1.63–4.21]	5.35 [3.14–7.56]	2.36 [1.26–3.46]
Interleukin‐6^†^, ^‡^, ^§^	0.60 [−0.01 to 1.21]	3.17 [1.78–4.56]	2.39 [1.27–3.51]
Tumour necrosis factor‐α^†^, ^‡^, ^§^	2.20 [1.16–3.24]	8.76 [5.21–12.31]	3.56 [2.03–5.09]
Testosterone^‡^, ^§^	0.58 [−0.03 to 1.19]	1.30 [0.54–2.06]	0.81 [0.16–1.46]
Cortisol	0.94 [0.25–1.63]	0.46 [−0.13 to 1.05]	−0.57 [−1.18 to 0.04]
Testosterone:cortisol ratio^§^	−0.64 [−1.27 to −0.01]	0.42 [−0.17 to 1.01]	1.20 [0.46–1.94]
Red blood cell count^†^, ^§^	−0.56 [−1.17 to 0.05]	0.47 [−0.12 to 1.06]	−1.11 [0.38–1.84]
Haemoglobin^†^, ^‡^	−0.83 [−1.48 to −0.18]	−0.58 [−1.19 to 0.03]	0.25 [−0.32 to 0.82]
Haematocrit^†^	−0.76 [−1.41 to −0.11]	−0.47 [−1.06 to 0.12]	0.29 [−0.28 to 0.86]
White blood cell count^†^	−0.68 [−1.31 to −0.05]	−0.40 [−0.99 to 0.19]	0.30 [−0.29 to 0.89]
Platelet count	−0.33 [−0.92 to 0.26]	−0.20 [−0.77 to 0.37]	0.19 [−0.38 to 0.76]
Countermovement jump height^‡^, ^§^	0.40 [−0.19 to 0.99]	0.97 [0.28–1.66]	0.61 [0.00–1.22]
Power of knee extensors, dominant leg^‡^, ^§^	0.61 [0.00–1.22]	1.33 [0.55–2.11]	0.64 [0.01–1.27]
Power of knee extensors, non‐dominant leg^‡^, ^§^	0.36 [−0.23 to 0.95]	1.10 [0.37–1.83]	0.73 [0.10–1.36]
Power of knee flexors, dominant leg^‡^, ^§^	0.09 [−0.48 to 0.66]	0.81 [0.16–1.46]	1.47 [0.65–2.29]
Power of knee flexors, non‐dominant leg^‡^, ^§^	0.18 [−0.39 to 0.75]	0.66 [0.03–1.29]	0.49 [−0.12 to 1.10]
10 m sprint time^†^, ^‡^	−2.20 [−3.24 to −1.16]	−2.75 [−3.98 to −1.52]	0.00 [−0.57 to 0.57]
Agility^†^, ^‡^, ^§^	−0.81 [−1.46 to −0.16]	−2.26 [−3.32 to −1.20]	−1.58 [−2.42 to −0.74]
Average 35 m running time during RAST^†^, ^‡^, ^§^	−0.71 [−1.34 to −0.08]	−2.13 [−3.15 to −1.11]	−1.60 [−2.46 to −0.74]
Speed drop during RAST^†^	−1.47 [−2.29 to −0.65]	−0.17 [−0.74 to 0.40]	0.67 [0.04–1.3]
Hamstring and lower back flexibility	0.13 [−0.44 to 0.70]	0.42 [−0.17 to 1.01]	0.26 [−0.31 to 0.83]
${\dot{V}}_{O_{2}\max}$ ^†^, ^‡^, ^§^	0.85 [0.18–1.52]	1.34 [0.56–2.12]	0.42 [−0.17 to 1.01]
$v{\dot{V}}_{O_{2}\max}$ ^†^, ^‡^, ^§^	0.77 [0.12–1.42]	1.84 [0.92–2.76]	1.09 [0.38–1.80]
*v*VT_2_ ^†^, ^‡^, ^§^	1.03 [0.32–1.74]	2.43 [1.31–3.55]	1.56 [0.72–2.40]
HR_max_ at ${\dot{V}}_{O_{2}\max}$ ^†^	−0.69 [−1.32 to −0.06]	−0.44 [−1.03 to 0.15]	0.16 [−0.41 to 0.73]
HR at VT_2_ ^‡^	−0.61 [−1.22 to 0.00]	−0.76 [−1.41 to −0.11]	−0.10 [−0.67 to 0.47]
Lactate at ${\dot{V}}_{O_{2}\max}$ ^†^, ^‡^	−1.21 [−1.95 to −0.47]	−1.17 [−1.90 to −0.44]	0.05 [−0.52 to 0.62]

Abbreviations: EP, extended play in friendly games during the late pre‐season preparation phase; HR, heart rate; HR_max_, maximal heart rate; MPS, middle of the pre‐season preparation phase; *n*, sample size; PC, 1 week prior to the initiation of the competition phase; PS, prior to the initiation of the 9 weeks of pre‐season preparation phase; RAST, running‐based anaerobic sprint test; ${\dot{V}}_{O_{2}\max}$, maximal oxygen uptake; VT_2_, second ventilatory threshold; $v{\dot{V}}_{O_{2}\max}$, velocity at ${\dot{V}}_{O_{2}\max}$; *v*VT_2_, velocity at VT_2_.

^†^ Significant difference between PS and MPS conditions at *P* ≤ 0.05.

^‡^ Significant difference between PS and PC conditions at *P* ≤ 0.05.

^§^ Significant difference between MPS and PC conditions at *P* ≤ 0.05.

**TABLE A6:** Cohen's *d* effect sizes and 95% confidence intervals, enclosed in square brackets, for pairwise comparisons between conditions (PS vs. MPS, PS vs. PC and MPS vs. PC) across all variables of the LP cohort (*n* = 12)

Variable	PS vs. MPS	PS vs. PC	MPS vs. PC
Body mass	−0.04 [−0.61 to 0.53]	0.07 [−0.50 to 0.64]	0.11 [−0.46 to 0.68]
Body fat	−0.17 [−0.74 to 0.40]	−0.34 [−0.93 to 0.25]	−0.17 [−0.74 to 0.40]
Rating of perceived exertion^†^, ^‡^	2.96 [1.65–4.27]	2.96 [1.65–4.27]	0.00 [−0.57 to 0.57]
Glucose	−0.3 [−0.89 to 0.29]	−0.26 [−0.83 to 0.31]	0.06 [−0.51 to 0.63]
Cholesterol^§^	−0.17 [−0.74 to 0.40]	0.34 [−0.25 to 0.93]	0.62 [−0.01 to 1.25]
Τriglycerides^§^	−0.88 [−1.55 to −0.21]	−0.23 [−0.8 to 0.34]	0.85 [0.18–1.52]
Serum glutamic–oxaloacetic transaminase	−0.14 [−0.71 to 0.43]	−0.21 [−0.78 to 0.36]	−0.11 [−0.68 to 0.46]
Serum glutamic–pyruvic transaminase	0.15 [−0.42 to 0.72]	0.31 [−0.28 to 0.90]	0.20 [−0.37 to 0.77]
Creatine phosphokinase	0.72 [0.09–1.35]	0.28 [−0.29 to 0.85]	−0.26 [−0.83 to 0.31]
Myoglobin^†^, ^§^	1.24 [0.50–1.98]	0.31 [−0.28 to 0.90]	−0.93 [−1.62 to −0.24]
Iron^†^	−0.81 [−1.46 to −0.16]	−0.42 [−1.01 to 0.17]	0.38 [−0.21 to 0.97]
Ferritin	−0.11 [−0.68 to 0.46]	−0.04 [−0.61 to 0.53]	0.09 [−0.48 to 0.66]
C‐Reactive protein	−0.47 [−1.06 to 0.12]	−0.46 [−1.05 to 0.13]	0.17 [−0.40 to 0.74]
High‐sensitivity C‐reactive protein^†^, ^§^	4.12 [2.38–5.86]	0.16 [−0.41 to 0.73]	−4.17 [−5.93 to −2.41]
Interleukin‐6^†^, ^§^	0.35 [−0.24 to 0.94]	−0.35 [−0.94 to 0.24]	−0.67 [−1.30 to −0.04]
Tumour necrosis factor‐α^†^, ^§^	2.60 [1.42–3.78]	−0.41 [−1.00 to 0.18]	−3.56 [−5.09 to −2.03]
Testosterone	−0.26 [−0.83 to 0.31]	0.17 [−0.40 to 0.74]	0.37 [−0.22 to 0.96]
Cortisol	0.31 [−0.28 to 0.90]	−0.07 [−0.64 to 0.50]	−0.36 [−0.95 to 0.23]
Testosterone:cortisol ratio	−0.50 [−1.11 to 0.11]	0.20 [−0.37 to 0.77]	0.67 [0.04–1.30]
Red blood cell count	−0.09 [−0.66 to 0.48]	0.20 [−0.37 to 0.77]	0.29 [−0.28 to 0.86]
Haemoglobin	−0.35 [−0.94 to 0.24]	−0.21 [−0.78 to 0.36]	0.20 [−0.37 to 0.77]
Haematocrit	−0.20 [−0.77 to 0.37]	−0.09 [−0.66 to 0.48]	0.10 [−0.47 to 0.67]
White blood cell count	0.01 [−0.56 to 0.58]	−0.03 [−0.60 to 0.54]	−0.03 [−0.60 to 0.54]
Platelet count	−0.18 [−0.75 to 0.39]	−0.43 [−1.02 to 0.16]	−0.25 [−0.82 to 0.32]
Countermovement jump height^‡^, ^§^	0.09 [−0.48 to 0.66]	0.51 [−0.10 to 1.12]	0.39 [−0.20 to 0.98]
Power of knee extensors, dominant leg^†^, ^‡^	0.78 [0.13–1.43]	0.77 [0.12–1.42]	0.03 [−0.54 to 0.60]
Power of knee extensors, non‐dominant leg^†^, ^‡^	0.85 [0.18–1.52]	0.64 [0.01–1.27]	−0.16 [−0.73 to 0.41]
Power of knee flexors, dominant leg	0.47 [−0.12 to 1.06]	0.43 [−0.16 to 1.02]	−0.03 [−0.60 to 0.54]
Power of knee flexors, non‐dominant leg	0.75 [0.10–1.40]	0.85 [0.18–1.52]	0.05 [−0.52 to 0.62]
10 m sprint time^†^, ^‡^	−1.14 [−1.87 to −0.41]	−1.43 [−2.23 to −0.63]	−0.29 [−0.86 to 0.28]
Agility^‡^, ^§^	−0.18 [−0.75 to 0.39]	−0.73 [−1.36 to −0.10]	−0.57 [−1.18 to 0.04]
Average 35 m running time during RAST^†^	−0.53 [−1.14 to 0.08]	0.04 [−0.53 to 0.61]	0.37 [−0.22 to 0.96]
Speed drop during RAST^†^	−1.03 [−1.74 to −0.32]	0.03 [−0.54 to 0.60]	1.17 [0.44–1.90]
Hamstring and lower back flexibility^†^, ^‡^	0.40 [−0.19 to 0.99]	0.65 [0.02–1.28]	0.26 [−0.31 to 0.83]
${\dot{V}}_{O_{2}\max}$ ^‡^, ^§^	0.37 [−0.22 to 0.96]	0.79 [0.14–1.44]	0.38 [−0.21 to 0.97]
$v{\dot{V}}_{O_{2}\max}$ ^†^	0.64 [0.01–1.27]	0.31 [−0.28 to 0.90]	−0.26 [−0.83 to 0.31]
*v*VT_2_ ^†^	0.73 [0.10–1.36]	0.34 [−0.25 to 0.93]	−0.45 [−1.04 to 0.14]
HR_max_ at ${\dot{V}}_{O_{2}\max}$	−0.34 [−0.93 to 0.25]	−0.40 [−0.99 to 0.19]	−0.07 [−0.64 to 0.50]
HR at VT_2_	−0.13 [−0.70 to 0.44]	−0.20 [−0.77 to 0.37]	−0.09 [−0.66 to 0.48]
Lactate at ${\dot{V}}_{O_{2}\max}$ ^‡^	0.26 [−0.31 to 0.83]	1.55 [0.71–2.39]	0.80 [0.15–1.45]

Abbreviations: HR, heart rate; HR_max_, maximal heart rate; LP, limited play in friendly games during the late pre‐season preparation phase; MPS, middle of the pre‐season preparation phase; *n*, sample size; PC, 1 week prior to the initiation of the competition phase; PS, prior to the initiation of the 9 weeks of pre‐season preparation phase; RAST, running‐based anaerobic sprint test; ${\dot{V}}_{O_{2}\max}$, maximal oxygen uptake; VT_2_, second ventilatory threshold; $v{\dot{V}}_{O_{2}\max}$, velocity at ${\dot{V}}_{O_{2}\max}$; *v*VT_2_, velocity at VT_2_.

^†^ Significant difference between PS and MPS conditions at *P* ≤ 0.05.

^‡^ Significant difference between PS and PC conditions at *P* ≤ 0.05.

^§^ Significant difference between MPS and PC conditions at *P* ≤ 0.05.

**TABLE A7:** Pearson's correlation coefficients (*r*) from pairwise comparisons between total time played in friendly games and physical, biochemical, hormonal, haematological and performance variables that were observed 1 week prior to the initiation of the competition phase in EP and LP cohorts and across all participants

Variable	EP (*n* = 12)	LP (*n* = 12)	Participants overall (total *n* = 24)
Body mass	0.36	0.44	0.32
Body fat	−0.13	−0.13	−0.47*
Rating of perceived exertion	0.54	0.39	−0.02
Glucose	0.02	0.08	0.36
Cholesterol	0.13	0.15	0.09
Τriglycerides	−0.14	−0.09	−0.14
Serum glutamic–oxaloacetic transaminase	0.15	0.04	0.22
Serum glutamic–pyruvic transaminase	0.04	0.30	0.12
Creatine phosphokinase	0.44	−0.11	−0.10
Myoglobin	0.46	−0.25	0.13
Iron	−0.09	−0.14	−0.44*
Ferritin	−0.11	0.42	0.44*
C‐Reactive protein	0.15	0.32	0.28
High‐sensitivity C‐reactive protein	0.38	−0.29	0.86*
Interleukin‐6	−0.35	0.17	0.79*
Tumour necrosis factor‐α	−0.43	−0.03	0.82*
Testosterone	0.03	0.09	0.21
Cortisol	−0.15	0.14	−0.03
Testosterone:cortisol ratio	0.10	−0.08	0.21
Red blood cell count	0.03	0.20	0.15
Haemoglobin	0.34	−0.33	−0.03
Haematocrit	0.36	−0.16	−0.02
White blood cell count	0.19	0.23	0.13
Platelet count	0.19	0.25	0.22
Countermovement jump height	0.23	0.14	0.10
Power of knee extensors, dominant leg	−0.09	0.33	0.55*
Power of knee extensors, non‐dominant leg	−0.24	0.14	0.44*
Power of knee flexors, dominant leg	−0.15	0.39	0.67*
Power of knee flexors, non‐dominant leg	−0.16	0.13	0.49*
10 m sprint time	−0.38	0.39	0.38
Agility	−0.22	−0.22	−0.37
Average 35 m running time during RAST	−0.13	−0.13	−0.52*
Speed drop rate during RAST	0.49	0.48	0.24
Hamstring and lower back flexibility	0.12	−0.11	0.16
${\dot{V}}_{O_{2}\max}$	−0.06	−0.42	−0.09
$v{\dot{V}}_{O_{2}\max}$	−0.26	−0.38	0.38
*v*VT_2_	−0.40	−0.48	0.55*
HR_max_ at ${\dot{V}}_{O_{2}\max}$	−0.41	0.14	−0.27
HR at VT_2_	−0.41	0.13	−0.32
Lactate at ${\dot{V}}_{O_{2}\max}$	−0.42	0.03	−0.62*

Abbreviations: EP, extended play; HR, heart rate; HR_max_, maximal heart rate; *n*, sample size; RAST, running‐based anaerobic sprint test; ${\dot{V}}_{O_{2}\max}$, maximal oxygen uptake; VT_2_, second ventilatory threshold; $v{\dot{V}}_{O_{2}\max}$, velocity at ${\dot{V}}_{O_{2}\max}$; *v*VT_2_, velocity at VT_2_.

* Significant correlation at *P* < 0.05.
