# Supplementary material for: Pre‐season friendly match‐play as a double‐edged sword: Performance gains and inflammatory cost in semi‐professional soccer players (a natural experiment)
Source: Exp Physiol. 2026 Oct 27:10.1113/EP093797. Online ahead of print. doi: 10.1113/EP093797 (PMC13711044; doi:10.1113/EP093797)
Supplement: Supplementary file 1 [file EPH-9999-0-s001.pdf]

Disclaimer: The authors had no involvement in the design, planning, or implementation of the training programme described in this study. All aspects of the programme—including structure, content, and delivery—were the sole responsibility of the team's coaching and conditioning staff. The training overview is presented exactly as it was provided by the technical staff for the purposes of this publication. The authors make no representations or warranties regarding the scientific justification, accuracy, or reliability of the programme, including but not limited to the selection of exercises, terminology used, work-to-rest ratios, or any methodological considerations. Furthermore, the authors disclaim all liability and responsibility for any reliance placed on the training content and are not responsible for any errors, omissions, or misinterpretations that may have arisen from the translation, adaptation, or reporting of the material by the technical staff.

Week 1: General Preparation Phase – Training Programme (12 hours/week)

Objective: Develop foundational physical fitness, general strength, aerobic base, mobility, and technical skill execution under low tactical demand.

Overview

- **Total weekly duration**: 12 hours
- **Training days**: 6 (Monday–Saturday)
- **Rest day**: Sunday
- **Daily average duration**: ~2 hours (varies slightly depending on training emphasis)
- **Intensity**: Low to moderate (RPE 4–6/10), progressive loading
- **Session structure**: Warm-up → Main training component → Technical drills → Cool-down/mobility

---

Day 1 – Monday

Focus: Aerobic endurance, basic strength (upper/lower body), technical drills
Duration: 2 hours

- **Warm-up (15 min):**
  - Dynamic mobility (hip openers, hamstring sweeps, high knees)
  - Light aerobic jogging (5 min)
  - Movement prep drills (A/B skips, carioca, arm swings)
- **Aerobic Conditioning (30 min):**
  - **Mode:** Extensive interval running
  - **Format:** 4 × 4 min at 75–80% HRmax, 2 min passive recovery
  - **Goal:** Build aerobic capacity and tolerance for volume
- **Strength Training (Upper Body – 30 min):**
  - 3 sets of 8–10 reps each, 60–90s rest
  - Bench press (moderate load, 65–70% 1RM)
  - Pull-ups or lat pulldown
  - Dumbbell shoulder press
  - Core circuit: plank holds (3 × 30s), side plank + reach, Russian twists
- **Technical Drills (30 min):**
  - Passing under pressure (4v2 rondos, 3 × 4 min bouts)
  - First touch and wall passes in pairs (5 × 1.5 min, 30s rest)
  - Dribbling through cones with progressive tempo (3 × 2 min, 1 min rest)
- **Cool-down (15 min):**
  - Light jogging (5 min), static stretching (10 min)

Day 2 – Tuesday

Focus: Strength (lower body), sprint mechanics, technical control
Duration: 2 hours

- **Warm-up (15 min):**
  - Activation: miniband glute bridges, monster walks
  - Sprint drills: A-skips, butt kicks, ankling
- **Lower Body Strength (45 min):**
  - Squats (4 × 6 reps @ 70% 1RM)
  - Romanian deadlifts (3 × 8 reps)
  - Step-ups with dumbbells (3 × 8 reps/leg)
  - Hamstring curls or Nordic curls (3 × 6 reps)
- **Sprint Mechanics & Acceleration (30 min):**
  - Wall drills (3 × 10s holds per leg)
  - Falling starts (6 × 10m)
  - Sled resisted sprints (4 × 10m, 90s rest)
  - Free sprints (4 × 20m, full recovery)
- **Technical Drills (20 min):**
  - Juggling circuit
  - Ball control under pressure (1v1 shielding in 5m x 5m grid, 3 × 2 min)
- **Cool-down (10 min)**

Day 3 – Wednesday

Focus: Aerobic endurance, flexibility, technical-tactical foundation
Duration: 2 hours

- **Warm-up (15 min)**
- **Aerobic Continuous Running (40 min):**
  - **Intensity:** 65–70% HRmax (conversational pace)
  - **Mode:** Grass surface, with ball (dribble laps every 5 min)
- **Flexibility/Movement Training (20 min):**
  - Mobility flow (e.g., hip 90/90s, thoracic rotations)
  - Partner-assisted stretches and proprioceptive neuromuscular facilitation (PNF)
- **Technical–Tactical Foundation (45 min):**
  - Shadow play (positional unopposed movements, 10 min)
  - Basic tactical pattern play (e.g., buildup from back, 3 × 5 min with coach cueing)
  - 7v0 passing shape drill (3 × 5 min)
- **Cool-down (10 min)**

Day 4 – Thursday

Focus: Strength (full-body circuit), agility, ball control under fatigue
Duration: 2 hours

- **Warm-up (15 min)**
- **Strength Circuit (45 min):**
    - **Format:** 5 stations, 3 rounds, 45s work / 15s transition
    - Box jumps, push-ups, kettlebell swings, Bulgarian split squats, medicine ball slams
    - Rest 2 minutes between rounds
- **Agility & Change of Direction (30 min):**
    - T-drill and zig-zag runs (4 × each)
    - Reactive cone drills with coach signal (4 × 15s bouts, 45s rest)
    - Ladder drills (3 rounds of 4 patterns)
- **Technical Drills (20 min):**
    - Small-sided rondo under fatigue (3v1, 4 × 2 min)
    - Passing accuracy challenge (5 reps × target zones, scored)
- **Cool-down (10 min)**

Day 5 – Friday

Focus: Small-sided game, tactical concepts, aerobic maintenance
Duration: 2 hours

- **Warm-up (15 min)**
- **Small-Sided Games (SSGs) (45 min):**
    - 4 × 5 min games (4v4 + goalkeepers, 2 min rest)
    - Emphasis on pressing, transition, team shape
    - Coach constraints: 2-touch, play wide, target forward runs
- **Tactical Principles (30 min):**
    - Unit coordination (defensive line movement in 3s/4s)
    - Attacking overlaps and underlaps (positional groups)
    - 6v4 overload in final third (3 × 3 min with cues)
- **Aerobic Maintenance (20 min):**
    - Ball possession in large grid (7v7 + 2 neutrals, 3 × 5 min bouts)
- **Cool-down (10 min)**

Day 6 – Saturday

Focus: Recovery modalities, mobility, low-load technical work
Duration: 1 hour

- **Warm-up & Mobility (15 min):**
    - Dynamic yoga sequence
    - Foam rolling

- **Low-Intensity Technical Circuit (30 min):**
    - Passing & receiving (5 patterns × 2 min each)
    - Target dribbling through cones
    - Light juggling, volley passes in pairs
- **Stretching & Breathing (15 min):**
    - Guided breath work
    - Static stretching (hip flexors, hamstrings, calves, shoulders)

Day 7 – Sunday

Rest day

- No structured training
- Hydration/nutrition guidelines provided
- Passive recovery (encouraged: hydrotherapy, nutrition, soft tissue)
- Optional light stretching or mindfulness session
- Emphasis on sleep hygiene and nutrition

Week 2: General Preparation Phase – Training Programme (12 hours/week)

Objective: Progress general conditioning, introduce anaerobic elements, enhance lower-limb power, increase technical demands under pressure, and begin tactical cueing in small-sided scenarios.

Overview

- **Total weekly duration**: 12 hours
- **Training days**: 6 (Monday–Saturday)
- **Rest day**: Sunday
- **Daily average duration**: ~2 hours
- **Intensity:** Moderate to moderately high (RPE 5–7/10)
- **Structure:** Warm-up → Main components → Technical/tactical drills → Cool-down

---

Day 1 – Monday

Focus: Aerobic intervals, upper body strength, passing/possession
Duration: 2 hours

- **Warm-up (15 min):**
  - Movement prep, coordination ladder, ball juggling
- **Aerobic Intervals (30 min):**
  - 5 × 4 min runs at 80% HRmax, 2 min walk recovery
  - Final rep with the ball (continuous dribble)
- **Strength – Upper Body (30 min):**
  - Bench press (4 × 6 reps @ 75% 1RM)
  - Pull-ups (3 × 8 reps)
  - Push press (3 × 6 reps)
  - Core circuit: weighted sit-ups, hanging leg raises, bird-dogs (3 rounds)
- **Technical Drill – Positional Rondo (30 min):**
  - 5v2 Rondo, 4 × 3 min bouts
  - Positional play in 6v4 zone-to-zone progression
  - 1-touch pass rule in final bout
- **Cool-down (15 min)**

Day 2 – Tuesday

Focus: Lower-limb strength and power, sprint work, dribbling under fatigue
Duration: 2 hours

- **Warm-up (15 min):**
  - Dynamic mobility + plyometric activation (mini-hops, pogo jumps)
- **Strength – Lower Body (40 min):**
  - Back squats (4 × 6 @ 75% 1RM)
  - Bulgarian split squats (3 × 8/leg)
  - Romanian deadlifts (3 × 8 reps)
  - Box jumps (3 × 5 reps)
- **Sprint Mechanics & Resisted Acceleration (30 min):**
  - Band-assisted wall sprints (4 × 10s)
  - Free sprints (4 × 20m)
  - Resisted sled sprints (4 × 15m @ 15% BW)
  - Contrast sprints: 2 × 10m resisted + 10m free
- **Technical Drill – Dribbling + Finish (20 min):**
  - 4 × 2 min dribble + shoot (through gates then finish)
  - 1v1 sprint-start dribble duels
- **Cool-down (15 min)**

Day 3 – Wednesday

Focus: Aerobic continuous running, tactical positioning, agility
Duration: 2 hours

- **Warm-up (15 min):**
  - Light jog, mobility circuit, ball rolling
- **Continuous Aerobic Conditioning (40 min):**
  - 70% HRmax jog on pitch with ball carries (light pressure every 10 min)
- **Tactical Positional Play (40 min):**
  - Shadow play: transitions from defence to midfield (15 min)
  - Positional circuit with coach cue (4 × 5 min)
  - Tactical pattern with CM buildup to final third (3 reps per unit)
- **Agility & Reaction Training (15 min):**
  - Light cone drills + coach clap/shout response
  - 1v1 mirror reaction games (3 × 20s per player)
- **Cool-down (10 min)**

Day 4 – Thursday

Focus: Functional strength circuit, technical circuits, possession under fatigue
Duration: 2 hours

- **Warm-up (15 min)**
- **Functional Strength Circuit (40 min):**
  - 6 stations: sled push, kettlebell swings, single-leg hops, push-ups, jump squats, MB rotational throws
  - 3 rounds, 40s work / 20s rest, 2 min rest between rounds
- **Technical Circuit (30 min):**
  - Station 1: Wall passes + cone dribble
  - Station 2: Aerial control + pass
  - Station 3: Inside-outside dribble weave
  - 2 rounds, 1 min per station, 30s transition
- **Possession Game – Fatigue (20 min):**
  - 5v5 in 25m x 25m grid with 2 floaters
  - 4 × 3 min bouts, 1 min rest
  - Last 2 bouts under high tempo with touch limit
- **Cool-down (15 min)**

Day 5 – Friday

Focus: Small-sided game, anaerobic conditioning, defensive shape
Duration: 2 hours

- **Warm-up (15 min)**
- **SSG – High Intensity (45 min):**
  - 4v4 + GKs, 3 × 4 min, 2 min rest
  - Constraints: 2-touch, offside line, scoring bonus for recovery runs
- **Anaerobic Runs (20 min):**
  - 6 × 20s maximal shuttle (20m distance), 1:3 work:rest
  - 4 × 30m sprint → deceleration + backpedal
- **Tactical – Defensive Shape (25 min):**
  - Defensive line movement as 4s
  - 6v5 transition game (press + recover)
  - Team coordination drill with shifting and covering zones
- **Cool-down (15 min)**

Day 6 – Saturday

Focus: Active recovery, technique refinement, mobility
Duration: 1 hour

- **Warm-up (10 min)**
- **Technical Drills (30 min):**
  - Free juggling, long passing challenge (accuracy scoring)
  - Volley-passes and heading control with partner
  - 5v2 possession (light intensity)
- **Mobility and Recovery (20 min):**

    - Foam rolling, dynamic mobility flow
    - Breathing drills + static stretching

Day 7 – Sunday

Rest day

- No structured training
- Hydration/nutrition guidelines provided
- Passive recovery (encouraged: hydrotherapy, nutrition, soft tissue)
- Optional light stretching or mindfulness session
- Emphasis on sleep hygiene and nutrition

Week 3: General Preparation Phase – Training Programme (12 hours/week)

Objective: Progress aerobic-anaerobic capacity, increase neuromuscular and metabolic load, integrate full-pitch tactical patterns, emphasize reactive decision-making and efficiency under fatigue.

Overview

- **Total weekly duration**: 12 hours
- **Training days**: 6 (Monday–Saturday)
- **Rest day**: Sunday
- **Average daily duration**: ~2 hours
- **Overall intensity:** Moderate to high (RPE 6–8/10)
- **Focus:** Training specificity increases; transition toward special preparation

---

Day 1 – Monday

Focus: Aerobic power intervals, upper body hypertrophy, possession under pressure
Duration: 2 hours

- **Warm-up (15 min)**
  - Dynamic drills, progressive skipping + dribbling, 2 min rondo
- **Aerobic Power – Intervals (30 min)**
  - 6 × 3 min at 85–90% HRmax, 1.5 min recovery jog
  - Last 2 reps with ball carry or passing partners every 20m
- **Strength – Upper Body Hypertrophy (30 min)**
  - Incline DB press (4 × 8 @ 70% 1RM)
  - Lat pulldown (3 × 10), push-up rows (3 × 8), dips (2 × 10)
  - Core superset: planks (45s), MB Russian twists (15/side), reverse crunches (3 rounds)
- **Possession Drill – High Pressure (30 min)**
  - 6v4 rondo in 15×15m grid, 5 × 2 min bouts
  - 6v6 conditioned possession: only 1-touch allowed after second pass (3 × 3 min)
- **Cool-down (15 min)**

Day 2 – Tuesday

Focus: Explosive lower-body strength, deceleration mechanics, 1v1 transitions
Duration: 2 hours

- **Warm-up (15 min)**
  - Ladder drills, mini hurdle hops, single-leg balance series
- **Strength – Lower Body Max/Power (40 min)**
  - Deadlift (4 × 5 @ 80% 1RM)
  - Jump squats (3 × 6 @ 40% 1RM)
  - Lateral bounds (3 × 8/side)
  - Sled push (3 × 15m @ 20% BW)
- **Agility & Deceleration (20 min)**
  - Y-drill decision sprint (3 reps/side)
  - Sprint 10m → decel zone → cone cut (6 reps)
  - Single-leg landing + control (2 × 5 each leg)
- **1v1 Transitions (25 min)**
  - Player A sprints 10m and defends against Player B carrying the ball
  - 3 × 30s bouts per pair, 2 rotations each
  - Focus: delay, angle, regain + counterattack
- **Cool-down (20 min)**

Day 3 – Wednesday

Focus: Long duration aerobic tempo + tactical full-pitch scenarios
Duration: 2 hours

- **Warm-up (15 min)**
  - Ball rolling + tactical visualization (coach-led)
- **Aerobic Tempo Run (40 min)**
  - 70–75% HRmax continuous run on pitch, with 4 × 2 min interruptions for shadow pressing
- **Tactical Drill – Full-Pitch Offensive Shape (40 min)**
  - Build-up from back 4 with pressing opposition mannequins
  - Full 11v0 shadow transitions with zone entry + final third execution
  - 3 × 6 min sequences, alternating flank and central build-up
- **Small Tactical Game (15 min)**
  - 8v8 + 2 floaters, 1-touch condition in attacking third
  - Emphasis on spacing, compactness in transition
- **Cool-down (10 min)**

Day 4 – Thursday

Focus: Functional circuits, ball control + movement mechanics
Duration: 2 hours

- **Warm-up (15 min)**
  - Mobility flow + plyometric warm-up (skater hops, pike jumps)
- **Functional Power Circuit (40 min):**
  - Stations (3 rounds, 45s work / 15s rest):
    1. MB slams
    2. Kettlebell goblet squat
    3. TRX rows
    4. Standing cable rotation
    5. Single-leg box hop
    6. Farmer carry (20m return)
- **Technical Drill – Control + Movement (30 min):**
  - Wall pass + angle dribble (5 reps each side)
  - Control + volley pass in small triangle drills
  - Repeated ball recovery + 1-touch control under pressure (3 reps × 1 min each)
- **Cognitive Load Drill – Reactive Decisions (20 min):**
  - Coach calls trigger: players must switch drill station or movement instantly
  - Quick transitions from control to pass, or receive and switch side
  - Decision reaction drill: color or voice-coded ball target
- **Cool-down (15 min)**

Day 5 – Friday

Focus: Anaerobic capacity + advanced small-sided games
Duration: 2 hours

- **Warm-up (15 min)**
- **Anaerobic Running (25 min):**
  - 5 × 20s all-out shuttle runs (20m zone), 1:3 work:rest
  - 4 × 30m sprint with flying start
  - 3 × 15m repeated sprint with decel to 50%
- **SSG – Tactical Overload (45 min):**
  - 6v5 + GKs in 30x25m pitch, focus on recovery and vertical passes
  - 3 × 5 min bouts with 2 min recovery
  - Constraint: 2 passes minimum before attacking move
  - Coach adjusts pressing cues after each bout
- **Set-Piece Drill (15 min):**
  - Repeated corners, indirect free kicks, and 2nd-ball scenarios
  - 2× each per team (offensive/defensive)
- **Cool-down (20 min)**

Day 6 – Saturday

Focus: Regenerative day, free play, team-building
Duration: 1 hour

- **Warm-up (10 min)**
  - Coordination games, light dynamic movements
- **Free Play Technical (25 min):**
  - Optional activities: keep-up challenge, volley tennis, rondo king of the hill
  - Fun + skill emphasis (no competitive pressure)
- **Mobility + Recovery (25 min):**
  - Foam rolling, guided breathwork, progressive stretching
  - Emphasis on hip, hamstring, adductor flexibility

Day 7 – Sunday

Rest Day

- No structured training
- Hydration/nutrition guidelines provided
- Passive recovery (encouraged: hydrotherapy, nutrition, soft tissue)
- Optional light stretching or mindfulness session
- Emphasis on sleep hygiene and nutrition

Week 4: General Preparation Phase – Training Programme (12 hours/week)

Objective: Consolidate training adaptations, enhance anaerobic efficiency, refine tactical principles. Final two days structured for tapering and recovery.

Overview

- **Total weekly duration:** 12 hours
- **Training days:** 6 (Monday–Saturday)
- **Rest day:** Sunday
- **Average daily duration:** ~2 hours (lighter on Fri–Sat)
- **Overall intensity:** Moderate to high (Mon–Thu); Low (Fri–Sat)
- **Focus:** Maximize adaptations while preventing cumulative fatigue before lab testing

---

Day 1 – Monday

Focus: Anaerobic intervals, upper-body strength, high-pressure possession
Duration: 2 hours

- **Warm-up (15 min)**
  - Dynamic mobility, coordination ladder, passing in pairs
- **Anaerobic Intervals (30 min)**
  - 3 sets of: 3 × 30s shuttle runs (15m zones) @ 90–95% max speed, 30s recovery
  - 3 min rest between sets
  - Emphasis on full stop-decel technique
- **Strength – Upper Body Power (30 min)**
  - Bench press (4 × 5 @ 80% 1RM)
  - Pull-ups (3 × 8)
  - Plyometric push-ups (3 × 5)
  - Landmine rotation (3 × 10/side)
- **Possession Under Pressure (30 min)**
  - 5v3 rondo (4 × 90s bouts, quick transition to next group)
  - 7v7 in 30×25m pitch, maximum 2 touches per player, 3 × 4 min bouts
  - Focus: quick scanning, close control, reactive pressure escape
- **Cool-down (15 min)**

Day 2 – Tuesday

Focus: Explosive lower-body strength, multidirectional speed, 2v2 game transitions
Duration: 2 hours

- **Warm-up (15 min)**
  - Hurdle mobility, linear skipping, single-leg bounding
- **Strength – Lower Body Explosiveness (35 min)**
  - Back squats (4 × 4 @ 85% 1RM)
  - Bulgarian split squat (3 × 6/leg)
  - Power cleans (3 × 4)
  - Nordic hamstring curls (2 × 5)
- **Agility & Speed Mechanics (20 min)**
  - Flying sprints (15m accel zone)
  - 5-10-5 shuttle with deceleration cues
  - Reactive cone-change patterns (4 reps each side)
- **Tactical Transition Drill – 2v2 (30 min)**
  - Start from midfield with controlled turnover
  - Teams of 2 must progress ball to end line under pressure
  - 4 × 90s bouts with 2 min rest, player rotation every set
- **Cool-down (20 min)**

Day 3 – Wednesday

Focus: High-intensity aerobic intervals, full-pitch tactical patterning
Duration: 2 hours

- **Warm-up (15 min)**
  - Jogging while passing, rondo 5v2 in 12m circle
- **Aerobic Intervals (30 min)**
  - 4 × 4 min at 85–90% HRmax with 2 min passive recovery
  - Ball included on last 2 reps: pass-and-move coordination within 20m lanes
- **Tactical Drill – Build-up Variability (35 min)**
  - Full-pitch 10v0 structured patterns:
    - Right/left flank rotation, inverted fullbacks
    - 3 × 5 min per pattern
  - Add passive defenders to increase perception-action coupling in final 10 min
- **Small-Sided Tactical Game (30 min)**
  - 6v6 + 2 floaters (25×20m pitch), emphasis on transitional compactness
  - 2-touch rule in defensive half, unlimited in attack
  - 3 × 4 min with 2 min rest
- **Cool-down (10 min)**

Day 4 – Thursday

Focus: Functional strength circuit, technical finishing, recovery strategies
Duration: 2 hours

- **Warm-up (15 min)**
  - Resistance bands, low hurdles, multi-plane mobility
- **Functional Circuit (35 min)**
  - 3 rounds:
    1. KB front squat (10 reps)
    2. Push-up to T-rotation (8/side)
    3. Med ball side throw (6/side)
    4. TRX hamstring curl (12 reps)
    5. Farmer carry 15m return
- **Finishing Drill (30 min)**
  - Cross + finish under defensive pressure (2v1 in final 18m box)
  - Ground ball, air cross, and cutback delivery
  - 2 × 4 min rounds with position rotation
- **Recovery Education & Application (30 min)**
  - Guided stretching, foam rolling
  - Player-led small group discussions: soreness rating, readiness scale, breathwork drills
- **Cool-down (10 min)**

Day 5 – Friday

Focus: Low-intensity technical drills and cognitive patterning
Duration: 1.5 hours

- **Warm-up (10 min)**
  - Mobility flow and light jogging
- **Technical Maintenance (40 min)**
  - Rondo 5v2 and 6v3 (6 × 1.5 min)
  - Pass-and-move in triangle circuits
  - Long passing accuracy to targets with receiver cue
- **Tactical Review – Walkthrough Patterns (30 min)**
  - Full-pitch walkthrough at <60% effort
  - Emphasis on positioning, verbal cues, visual triggers
- **Breath-Control + Mobility (10 min)**
  - Diaphragmatic breathing + hip/adductor mobility

Day 6 – Saturday

Focus: Light positional rondo, pre-test tactical overview
Duration: 1.5 hours

- **Warm-up (10 min)**
  - Dynamic stretching, light cone dribbling
- **Positional Rondo – Low Load (30 min)**
  - Groups of 3–5 within zonal constraints
  - Walk-to-jog tempo, one-touch emphasis in central channel
- **Tactical Review – Match Model (30 min)**
  - Coach walkthrough of team principles using whiteboard + half-pitch demo
  - Visual rehearsal of pressing, possession, and rest defense principles
- **Cool-down + Testing Brief (20 min)**
  - Light jog, foam roll, clear schedule and instructions for MPS testing

----

Day 7 – Sunday

Full Rest Day

- No structured training
- Hydration/nutrition guidelines provided
- Passive recovery (encouraged: hydrotherapy, nutrition, soft tissue)
- Optional light stretching or mindfulness session
- Emphasis on sleep hygiene and nutrition
- **Players reminded of test protocol for Monday (MPS time point)**

Week 5: Special Preparation Phase (12 hours/week)

Objective: Integrate match-specific fitness and tactical sharpness via increased match exposure (2 friendly matches). Manage cumulative fatigue through adjusted training load and recovery strategies.

Overview

- **Total weekly duration:** 12 hours
- **Training days:** 6 (Monday–Saturday)
- **Rest day:** Sunday
- **Average daily duration:** ~2 hours
- **Laboratory Testing (MPS time point):** Monday morning
- **Friendly Matches 2:** Wednesday & Saturday
- **Overall intensity:** Moderate-high (Tue, Fri); High (Wed, Sat); Light (Mon, post-testing)

Day 1 – Monday

Focus: Laboratory testing (MPS); recovery session afterward
Duration: 2 hours total (1.5 hours testing + 30 min active recovery)

- **Morning – Laboratory Testing (2-3 hours)**
    - **Measures:** Body mass, body composition, blood sampling, $\dot{V}O_2$max, jump power (CMJ, SJ), RAST test, and haematological markers
- **Afternoon – Recovery and Mobility (30 min)**
    - Guided breathwork
    - Low-intensity cycling (10 min)
    - Static stretching and foam rolling (20 min)

Day 2 – Tuesday

Focus: Tactical Strategy + Set Pieces (Match Prep)
Duration: 2 hours

- **Warm-up (15 min)**
    - Ball-feel + coordination ladder
    - Short-passing precision game
- **Tactical Periodization Drill (45 min)**
    - 10v8 functional game (e.g., midfield overload scenario)
    - High ball circulation focus + structured press escape

- **Set Pieces (30 min)**
    - Corners (defensive and offensive)
    - Free-kick wall formations and cues
    - Throw-in press traps
- **Small-Sided Game (20 min)**
    - 6v6 with tactical rules (limited touches, pressing zones)
- **Cool-down (10 min)**

Day 3 – Wednesday

Focus: FRIENDLY MATCH 1
Opponent: External team of similar competitive level
Duration: ~90 minutes (+ prep/recovery = 2.5 hours total)

- **Pre-Match Routine (30 min)**
    - Mobility + activation drills + dynamic warm-up
    - Tactical reminders and set piece cues
- **Match Play (2 x 45 min)**
    - All players ≥40 min participation; 3-period structure possible
    - Match filmed + GPS monitored
- **Post-Match Recovery (30 min)**
    - Light jogging or cycling (10 min)
    - Guided foam rolling and hydration protocol

Day 4 – Thursday

Focus: Active Recovery + Video Review
Duration: 1.5 hours

- **Video Review (20 min)**
    - Key tactical patterns from Match 1
    - Player feedback and correction points
- **Mobility & Ball Mastery (30 min)**
    - Active ROM, banded hip/glute work
    - Tight-space control drills
- **Low-Load Possession Game (20 min)**
    - 5v5 (keep intensity <70% HRmax)
    - Focus on ball movement, spatial awareness
- **Cool-down (10 min)**

Day 5 – Friday

Focus: Match 2 Tactical Prep + Activation
Duration: 1.5 hours

- **Warm-up & Ball Circulation (20 min)**
    - Passing under pressure + movement off ball
    - Reaction drills with dual-tasking
- **Tactical Shadow Play (40 min)**
    - 11v0 or 10v1 pattern rehearsal
    - Transition and zonal movement
- **Finishing Drills (15 min)**
    - 2v1 and 3v2 attacking situations
    - Low volume, high precision
- **Cool-down (15 min)**

Day 6 – Saturday

Focus: FRIENDLY MATCH 2
Opponent: Different external team
Duration: ~2.5 hours (incl. prep/recovery)

- **Pre-Match Prep (30 min)**
    - Coordination + light dynamic warm-up
    - Brief tactical prompts
- **Match Play (2 x 45 min)**
    - Moderate-to-high intensity, full-pitch dynamics
    - Tactical emphasis: pressing, recovery runs
- **Post-Match Recovery (30 min)**
    - Low-intensity cycle + nutrition & stretching

Day 7 – Sunday

Full Rest Day

- No structured training
- Hydration/nutrition guidelines provided
- Passive recovery (encouraged: hydrotherapy, nutrition, soft tissue)
- Optional light stretching or mindfulness session
- Emphasis on sleep hygiene and nutrition

Week 6: Special Preparation Phase (12 hours/week)

Objective: Continued tactical-tactical integration and competitive readiness through dual match exposure. Emphasis on functional intensity, situational awareness, and recovery between match-loads.

Overview

- **Total weekly duration:** 12 hours
- **Training days:** 6 (Monday–Saturday)
- **Rest day:** Sunday
- **Average daily duration:** ~2 hours
- **Friendly Matches 2:** Wednesday & Saturday
- **Overall intensity:** Moderate-high; light taper into Saturday match

---

Day 1 – Monday

Focus: Regeneration + Light Technical Execution
Duration: 1.5 hours

- **Warm-Up & Mobility (20 min)**
    - Soft tissue release (foam roll) + banded mobility
    - Low-impact core stability drills
- **Technical Circuit (40 min)**
    - Tight-space rondos (5v2, 6v3)
    - Short-passing precision and first-touch under light pressure
    - Short transitional movement drills with the ball
- **Small Positional Game (20 min)**
    - 3v3 + neutral player (target zones)
    - Low-intensity (HR < 70% max)
- **Cool-down (10 min)**
    - Static flexibility & guided diaphragmatic breathing

Day 2 – Tuesday

Focus: Tactical Systems + Match Prep
Duration: 2 hours

- **Warm-Up (15 min)**
    - Dynamic mobility with technical touches
    - Agility ladder with pass/receive under pressure
- **Functional Tactical Drill (45 min)**

- Full-width, half-pitch shape game (9v7)
- Focus: buildup from back, mid-third rotation, compact defensive shifts

- **Set Piece Scenarios (30 min)**
  - Simulated match corner/free kick routines
  - Offensive and defensive adjustments
- **Tactical Match Simulation (20 min)**
  - 10v10 short game with constraints (e.g., mandatory overlap, zonal presses)
- **Cool-down (10 min)**

Day 3 – Wednesday

Focus: FRIENDLY MATCH 1
Opponent: Balanced external team
Duration: ~2.5 hours

- **Pre-Match Routine (30 min)**
  - Functional activation + targeted passing warm-up
  - Team briefing with key objectives
- **Match Play (2 × 45 min)**
  - All players rotate to meet ≥45 min playing time, 3-period structure possible
  - Key focuses: vertical compactness, final-third entries
- **Post-Match Recovery (30 min)**
  - Stationary cycle (10 min), stretching, hydration, cold immersion (optional)

Day 4 – Thursday

Focus: Recovery + Tactical Review
Duration: 1.5 hours

- **Video Analysis (20 min)**
  - Review of positional behaviors and transitions from Match 1
- **Mobility & Ball Control (30 min)**
  - Band resistance stretches, deep lunge matrix
  - 2-touch technical passing games in grids
- **5v5 Tactical Possession Game (20 min)**
  - Minimal pressure
  - Target: ~65–70% HRmax, focus on spacing and support angles
- **Cool-down (10 min)**

Day 5 – Friday

Focus: Tactical Adjustments + Activation for Match 2
Duration: 1.5 hours

- **Warm-Up & Coordination (20 min)**
  - Fast feet drills, light deceleration work
  - Ball-mastery under fatigue
- **Tactical Adjustments (40 min)**
  - 11v0 pattern execution
  - Key roles rehearsed (e.g., press triggers, counters)
- **Finishing + Set Pieces (20 min)**
  - Crossing and finishing in groups of 3
  - Set piece variation dry-runs
- **Cool-down (10 min)**

Day 6 – Saturday

Focus: FRIENDLY MATCH 2
Opponent: Moderately stronger team
Duration: ~2.5 hours

- **Pre-Match Routine (30 min)**
  - Dynamic stretching, mobility flow, reactive coordination
  - Key verbal cues
- **Match Play (2 × 45 min)**
  - 80–90% match intensity
  - Emphasis on decision-making in possession under pressure
- **Post-Match Recovery (30 min)**
  - Foam roll, hydration, light jog/walk, compression (optional)

Day 7 – Sunday

Full Rest Day

- No structured training
- Hydration/nutrition guidelines provided
- Passive recovery (encouraged: hydrotherapy, nutrition, soft tissue)
- Optional light stretching or mindfulness session
- Emphasis on sleep hygiene and nutrition

Week 7: Special Preparation Phase – Training Programme (12 hours/week)

Objective: Optimize tactical readiness and in-game decision-making under fatigue with **Friendly Matches 5 & 6**, while maintaining neuromuscular and aerobic fitness.

Overview

- **Total weekly duration:** 12 hours
- **Training days:** 6 (Monday–Saturday)
- **Rest day:** Sunday
- **Friendly Match 5:** Wednesday
- **Friendly Match 6:** Saturday
- **Overall intensity:** Moderate; balanced around match days

---

Day 1 – Monday

Focus: Active recovery and cognitive reactivation
Duration: 1.5 hours

- **Warm-up (15 min)**
  - Low-intensity ball routines
  - Partner juggling and dynamic stretching
- **Tactical Recap & Walkthrough (30 min)**
  - On-field walk-through of key pressing and build-up patterns
  - Unopposed shape corrections (positional alignment)
- **Technical Routine (30 min)**
  - 5v2 rondos – 4 × 3 min bouts
  - Triangle passing with third-man runs
- **Mobility & Regeneration (15 min)**
  - Foam rolling, breathing drills, hydration protocol

Day 2 – Tuesday

Focus: Tactical execution and set-piece preparation
Duration: 2 hours

- **Warm-up (15 min)**
  - Dynamic mobility + speed ladder
- **Tactical Scenarios (45 min)**
  - 11v11 scenario-based play

        - High press transitions, wing overloads
        - 3 × 10 min segments, 3 min recovery
- **Set-Piece Focus (30 min)**
    - Offensive/defensive corners, free kicks (zonal & man-marked)
    - Simulated game-speed execution
- **Position-Specific Conditioning (20 min)**
    - Centre-backs: diagonal clearances under pressure
    - Midfielders: shuttle & decision sprints (3 × 15m + pass)
    - Forwards: curved runs to finish (2 × 6)
- **Cool-down (10 min)**

Day 3 – Wednesday

Focus: Friendly Match 5 – Execution Under Mid-Week Load
Duration: ~2 hours

- **Pre-Match Warm-up (25 min)**
    - Mobility + acceleration prep
    - Quick-fire ball drills
- **Match Play (90 min)**
    - Full competitive fixture vs. external team
- **Post-Match Recovery (30 min)**
    - Static stretching + cold water immersion
    - Team cooldown jog, RPE tracking

Day 4 – Thursday

Focus: Recovery & targeted technical activation
Duration: 1.5 hours

- **Mobility Activation (20 min)**
    - Deep tissue foam roll, breathing protocols
    - Ankle and hip mobility flow
- **Technical Circuit (30 min)**
    - Dribbling relay with precision passing
    - Wall passing & layoff work – 3 × 4 min rounds
- **Tactical Rehearsal (30 min)**
    - Functional positioning (3v2 and 4v3 patterns)
    - Emphasis on movement off the ball
- **Cool-down (10 min)**

Day 5 – Friday

Focus: Sharpness, tactical roles, and match readiness
Duration: 1.5 hours

- **Warm-up (15 min)**
  - Light plyometrics, neural activation
- **Tactical Rehearsal (30 min)**
  - Walk-through: specific roles and match expectations
  - Full-field positioning and transitions
- **Finishing Routine (20 min)**
  - Rebound volleys, timed headers, off-balance shots
  - 2 × 6 min bouts
- **Set-Pieces (15 min)**
  - Short corner and direct FK patterns
- **Cool-down (10 min)**

Day 6 – Saturday

Focus: Friendly Match 6 – End-of-Week Match Play
Duration: ~2 hours

- **Pre-Match Warm-up (25 min)**
  - Activation, sprint prep, short pass grids
- **Match Play (90 min)**
  - Full 11v11 match vs. external opponent
- **Post-Match Recovery (30 min)**
  - Cool-down jog, stretch, hydration check
  - Group feedback and match metrics review

Day 7 – Sunday

Full Rest Day

- No structured training
- Hydration/nutrition guidelines provided
- Passive recovery (encouraged: hydrotherapy, nutrition, soft tissue)
- Optional light stretching or mindfulness session
- Emphasis on sleep hygiene and nutrition

Week 8: Special Preparation Phase – Training Programme (12 hours/week)

Objective: Finalize tactical cohesion and match exposure (2 friendly games), while reducing cumulative load toward the end of the week to facilitate recovery ahead of post-conditioning (PC) lab testing.

Overview

- **Total weekly duration:** ~12 hours
- **Training days:** 6 (Monday–Saturday)
- **Rest day:** Sunday
- **Friendly Match 7:** Tuesday
- **Friendly Match 8:** Thursday
- **Low-intensity taper:** Friday & Saturday
- **Lab testing prep:** Sunday rest → PC testing Monday

Day 1 – Monday

Focus: Tactical rehearsal & offensive organization
Duration: 2 hours

- **Warm-up (15 min)**
  - Ball mobility + lateral movement prep
  - Pair-passing with change-of-direction accelerations
- **Tactical Organisation (45 min)**
  - Offensive shape under low-medium block
  - Progression and link-up play patterns
- **Set-Piece Reinforcement (30 min)**
  - Attacking corner routines (short, in-swing, near-post runs)
  - FK simulations (wide zone entries)
- **Cool-down (10 min)**
  - Static stretch + hydration feedback

Day 2 – Tuesday

Focus: Friendly Match 7
Duration: ~2 hours

- **Pre-Match Warm-up (25 min)**
  - Dynamic mobility + short-passing rondos
  - 3 × 15m accelerations, change-of-direction prep

- **Match Play (90 min)**
  - Full 11v11 match vs external opposition
- **Post-Match Recovery (30 min)**
  - Group cooldown jog
  - Guided foam rolling + cold immersion (as available)
  - RPE and match data collection

Day 3 – Wednesday

Focus: Recovery and brief positional technique
Duration: 1.5 hours

- **Mobility Circuit (20 min)**
  - Ankle/knee/hip joint flows + breathing reset drills
- **Technical Skills (30 min)**
  - 3v1 rondos (tight space)
  - Partner volleys and inside-foot control under time pressure
  - Quick-fire wall-passing
- **Tactical Concepts (30 min)**
  - Repetition of defensive block shape (no opposition pressure)
  - Low-tempo pattern runs for outside backs and midfield triangles
- **Cool-down (10 min)**

Day 4 – Thursday

Focus: Friendly Match 8
Duration: ~2 hours

- **Pre-Match Warm-up (25 min)**
  - Neural activation drills (fast feet, skips)
  - Short-sided ball grids
- **Match Play (90 min)**
  - Full 11v11 fixture vs external team
- **Post-Match Recovery (30 min)**
  - Recovery jog + contrast showers (if available)
  - Hydration review + muscle soreness self-report

Day 5 – Friday

Focus: Light technical/tactical taper
Duration: 1 hour

- **Warm-up (10 min)**
  - Ball juggling and light dynamic warm-up

- **Light Tactical Routine (30 min)**
  - Walk-through positional alignments
  - Communication-focused 5v5 shape (50% intensity)
- **Technical Maintenance (15 min)**
  - First-touch and one-touch rondos
  - Low-load shooting drills (no sprints)
- **Cool-down (5 min)**
  - Static stretching

Day 6 – Saturday

Focus: Pre-Test Recovery Session
Duration: 1 hour

- **Mobility & Core Stability (20 min)**
  - Deep breathing
  - Band work for shoulders/hips
  - Controlled glute activation
- **Technical Fun Drill (20 min)**
  - Low-pressure possession games (6v2 or 7v3)
  - Light finishing games with minimal running
- **Stretch & Visualization (20 min)**
  - Static mobility + mental rehearsal cues for testing

Day 7 – Sunday

Full Rest Day

- No structured training
- Hydration/nutrition guidelines provided
- Passive recovery (encouraged: hydrotherapy, nutrition, soft tissue)
- Optional light stretching or mindfulness session
- Emphasis on sleep hygiene and nutrition
- **Players reminded of test protocol for Monday (PC time point)**

Week 9: Tapering Phase – Training Programme (12 hours/week)

Objective: Facilitate physiological recovery, consolidate tactical organization, and maintain neuromuscular sharpness while avoiding residual fatigue.
Key Feature:

- **PC Laboratory Testing on Monday (Day 1)**
- **Total weekly volume:** ~12 hours
- **Training intensity:** Low to moderate, peaking briefly mid-week then tapering again
- **No friendly matches this week**
- **Final rest day on Sunday**

Day 1 – Monday: PC Laboratory Testing

Duration: ~2.5 hours
Focus: Post-conditioning (PC) testing of physiological and performance markers

- **Testing Protocols (Laboratory setting):**
    - Body composition (via skinfolds/BIA)
    - Blood sampling: creatine kinase, myoglobin, inflammatory cytokines, RBCs, etc.
    - Aerobic fitness test (e.g., $v\dot{V}O_2max$, vVT_2 determination)
    - Neuromuscular power (e.g., vertical jump with dominant/non-dominant leg)
    - Repeated anaerobic sprint test (RAST)
    - 35-m sprint performance
    - Subjective wellness and fatigue inventories
- **Post-Test Recovery (30 min):**
    - Low-intensity cycling or walking (10 min)
    - Guided foam rolling/stretch
    - Hydration and recovery nutrition guidance

Day 2 – Tuesday

Focus: Tactical organization & sharpness (low-moderate load)
Duration: 2 hours

- **Warm-up (15 min)**
    - Dynamic movements + technical ball control
    - Ladder footwork + light passing
- **Tactical Drill (40 min)**
    - Medium-intensity 8v6 attacking transition in zones

    - Position-specific decision-making sequences
- **Small-Sided Game (30 min)**
    - 5v5 with 2 neutral players (3 x 5 min, 2 min rest)
    - Moderate tempo, focus on possession under pressure
- **Finishing Drill (20 min)**
    - Low- to moderate-speed combinations ending in shots
    - 4 attackers vs passive 2 defenders
- **Cool-down (15 min)**
    - Static stretching + core activation

Day 3 – Wednesday

Focus: Recovery + Technical Precision
Duration: 1.5 hours

- **Mobility & Core (20 min)**
    - Hip/shoulder flossing
    - Breathing alignment + dead bug variations
- **Ball Mastery (30 min)**
    - Tight-area ball control
    - Short-distance passing under time constraints
    - First-touch drills with passive pressure
- **Technical-Tactical Circuit (30 min)**
    - Low-load pattern play (no opposition)
    - Defensive and attacking transitions walk-through
    - Repetitions of pressing triggers (50% intensity)
- **Cool-down (10 min)**

Day 4 – Thursday

Focus: Tactical cohesion & tempo stimulation
Duration: 2 hours

- **Warm-up (15 min)**
    - Short passing, dynamic mobility, and agility ladder
- **Positional Play (30 min)**
    - 7v7 half-pitch with restricted touches
    - Emphasis on zone occupation and transitions
- **High-Speed Tactical Sequence (30 min)**
    - 11v11 walk-through → 50% tempo → 80% tempo run-through
    - Instructions on set pressing and final-third movement
- **Shooting Routines (20 min)**
    - Fast passing combinations ending in 1-touch shots
    - Strikers and midfielders rotate roles
- **Cool-down (10 min)**
    - Light jog and dynamic stretch

- Breathwork and hydration

Day 5 – Friday

Focus: Activation and Ball Feeling
Duration: 1.5 hours

- **Dynamic Prep (15 min)**
  - Mini-bands + coordination footwork
  - Partner passing sequences
- **Light Tactical Drill (30 min)**
  - Positional rondos
  - Midfield pivot drills (switching + off-ball movement)
- **Mini-Games (20 min)**
  - 4v4 tournaments with 1-touch limit
  - Short field, short duration (3 x 3 min)
- **Cool-down (10 min)**
  - Static stretch and foam rolling

Day 6 – Saturday

Focus: Final tuning session (light, enjoyable, low load)
Duration: 1 hour

- **Warm-up (10 min)**
  - Ball control + technical relay challenges
- **Fun-Based Possession Drill (20 min)**
  - 7v3 keep-away game with varying rules
  - Encourage high communication and creativity
- **Tactical Review (20 min)**
  - Coach-led walk-through of set pieces, matchday strategy
  - Emphasis on team shape, roles, and cue recognition
- **Cool-down (10 min)**
  - Team circle stretch + motivation talk

Day 7 – Sunday

Full Rest Day

- No formal training
- Optional mobility routine at home
- Nutritional readiness and sleep quality emphasized
