# Supplementary material for: Pre‐season friendly match‐play as a double‐edged sword: Performance gains and inflammatory cost in semi‐professional soccer players (a natural experiment)
Source: Exp Physiol. 2026 Oct 27:10.1113/EP093797. Online ahead of print. doi: 10.1113/EP093797 (PMC13711044; doi:10.1113/EP093797)
Supplement: Supplementary file 2 [file EPH-9999-0-s002.docx]

| **Table A1.** Physical, biochemical, hormonal, haematological, and performance data of the EP cohort (n=12) for PS, MPS, and PC conditions, presented as M±SD[95%CI]. | | | |
| --- | --- | --- | --- |
| **Variable** | **PS** | **MPS** | **PC** |
| ‡§ Body mass (kg) | 76.71±5.01[73.88–79.54] | 76.15±5.53[73.02–79.28] | 74.74±5.82[71.45–78.03] |
| †‡§ Body fat (%) | 12.49±1.81[11.47–13.51] | 10.63±1.84[9.59–11.67] | 9.25±1.86[8.20–10.30] |
| †‡ Rate of perceived exertion (Borg scale, 1–10) | 4.92±0.9[4.41–5.43] | 7.42±0.51[7.13–7.71] | 7.50±0.52[7.21–7.79] |
| Glucose (mg·dl⁻¹) | 91.00±10.43[85.10–96.90] | 82.50±7.68[78.15–86.85] | 89.08±5.90[85.74–92.42] |
| ‡§ Cholesterol (mg·dl⁻¹) | 154.50±25.50[140.07–168.93] | 160.33±27.50[144.77–175.89] | 170.83±29.97[153.87–187.79] |
| § Τriglycerides (mg·dl⁻¹) | 86.67±34.92[66.91–106.43] | 74.17±20.14[62.77–85.57] | 86.17±20.28[74.70–97.64] |
| ‡§ Serum glutamic-oxaloacetic transaminase (U·l⁻¹) | 21.67±5.68[18.46–24.88] | 25.25±4.27[22.83–27.67] | 29.50±7.14[25.46–33.54] |
| ‡ Serum glutamic-pyruvic transaminase (U·l⁻¹) | 19.00±8.43[14.23–23.77] | 23.67±5.97[20.29–27.05] | 26.42±8.36[21.69–31.15] |
| ‡ Creatine phosphokinase (U·l⁻¹) | 136.83±63.38[100.97–172.69] | 208.00±74.32[165.95–250.05] | 232.25±86.8[183.14–281.36] |
| †‡ Myoglobin (mg·l⁻¹) | 37.83±16.79[28.33–47.33] | 72.42±31.53[54.58–90.26] | 83.33±50.44[54.79–111.87] |
| Iron (μg·dl⁻¹) | 82.25±40.01[59.61–104.89] | 73.67±12.92[66.36–80.98] | 79.58±15.22[70.97–88.19] |
| Ferritin (mg·l⁻¹) | 93.28±48.49[65.84–120.72] | 78.70±26.77[63.55–93.85] | 74.09±26.12[59.31–88.87] |
| C-reactive protein (mg·l⁻¹) | 0.30±0.06[0.27–0.33] | 0.39±0.11[0.33–0.45] | 0.36±0.11[0.30–0.42] |
| †‡§ Hs C-reactive protein (mg·l⁻¹) | 0.89±0.23[0.76–1.02] | 1.62±0.27[1.47–1.77] | 2.28±0.28[2.12–2.44] |
| †‡§ Interleukin-6 (mg·l⁻¹) | 8.50±1.83[7.46–9.54] | 9.63±1.94[8.53–10.73] | 13.79±1.44[12.98–14.60] |
| †‡§ Tumour necrosis factor alpha (pg·ml⁻¹) | 5.71±0.66[5.34–6.08] | 7.95±1.17[7.29–8.61] | 11.58±0.68[11.20–11.96] |
| ‡§ Testosterone (ng·ml⁻¹) | 4.73±0.60[4.39–5.07] | 5.41±1.36[4.64–6.18] | 6.66±1.69[5.70–7.62] |
| Cortisol (μg·dl⁻¹) | 14.28±4.63[11.66–16.90] | 18.05±1.99[16.92–19.18] | 16.23±3.71[14.13–18.33] |
| § Testosterone–cortisol ratio (T:C) | 0.37±0.13[0.30–0.44] | 0.30±0.08[0.25–0.35] | 0.42±0.11[0.36–0.48] |
| †§ Red blood cell count (10¹²RBC·l⁻¹) | 5.35±0.60[5.01–5.69] | 5.04±0.47[4.77–5.31] | 5.63±0.57[5.31–5.95] |
| †‡ Haemoglobin (g·dl⁻¹) | 15.50±1.44[14.69–16.31] | 14.25±1.58[13.36–15.14] | 14.64±1.53[13.77–15.51] |
| † Haematocrit (%) | 46.18±3.88[43.98–48.38] | 43.19±3.95[40.96–45.42] | 44.33±4.01[42.06–46.60] |
| † White blood cell count (10⁹WBC·l⁻¹) | 7.03±1.53[6.16–7.90] | 6.12±0.99[5.56–6.68] | 6.46±1.25[5.75–7.17] |
| Platelet count (10⁹PLT·l⁻¹) | 227.58±34.14[208.26–246.90] | 206.65±73.35[165.15–248.15] | 219.08±46.50[192.77–245.39] |
| ‡§ Countermovement jump height (cm) | 39.59±2.63[38.10–41.08] | 40.59±2.37[39.25–41.93] | 42.11±2.57[40.66–43.56] |
| ‡§ Power of knee extensors, dominant leg (watt) | 198.17±16.02[189.11–207.23] | 208.83±18.44[198.40–219.26] | 220.33±17.22[210.59–230.07] |
| ‡§ Power of knee extensors, non-dominant leg (watt) | 201.00±18.76[190.39–211.61] | 207.83±18.70[197.25–218.41] | 221.17±18.01[210.98–231.36] |
| ‡§ Power of knee flexors, dominant leg (watt) | 143.50±23.77[130.05–156.95] | 145.42±9.80[139.88–150.96] | 160.25±10.38[154.38–166.12] |
| ‡§ Power of knee flexors, non-dominant leg (watt) | 137.83±20.67[126.14–149.52] | 141.42±20.14[130.02–152.82] | 150.92±18.93[140.21–161.63] |
| †‡ 10-m sprint time (s) | 1.82±0.05[1.79–1.85] | 1.71±0.04[1.69–1.73] | 1.71±0.03[1.69–1.73] |
| †‡§ CODS (s) | 16.50±0.32[16.32–16.68] | 16.25±0.30[16.08–16.42] | 15.73±0.36[15.53–15.93] |
| †‡§ Average 35-m running time during RAST (s) | 5.17±0.15[5.09–5.25] | 5.07±0.13[5.00–5.14] | 4.83±0.16[4.74–4.92] |
| † Speed drop during RAST (%) | 9.90±20[8.77–11.03] | 7.36±1.10[6.74–7.98] | 9.39±3.41[7.46–11.32] |
| Hamstring and lower back flexibility (cm) | 23.58±6.32[20.00–27.16] | 24.42±6.71[20.62–28.22] | 26.00±4.79[23.29–28.71] |
| †‡§ V̇O₂max (ml·kg⁻¹·min⁻¹) | 51.89±4.21[49.51–54.27] | 55.57±4.48[53.04–58.10] | 57.37±3.94[55.14–59.60] |
| †‡§ vV̇O₂max (km·hr⁻¹) | 15.96±0.86[15.47–16.45] | 16.63±0.88[16.13–17.13] | 17.63±0.96[17.09–18.17] |
| †‡§ vVT₂ (km·hr⁻¹) | 12.08±0.79[11.63–12.53] | 12.83±0.65[12.46–13.20] | 13.83±0.62[13.48–14.18] |
| † HRmax at V̇O₂max (b·min⁻¹) | 194.33±7.38[190.15–198.51] | 189.33±7.14[185.29–193.37] | 190.67±8.87[185.65–195.69] |
| ‡ HR at VT₂ (b·min⁻¹) | 166.67±5.73[163.43–169.91] | 163.00±6.27[159.45–166.55] | 162.42±5.42[159.35–165.49] |
| †‡ Lactate at V̇O₂max (mmol·l⁻¹) | 13.01±2.08[11.83–14.19] | 10.77±1.45[9.95–11.59] | 10.84±1.51[9.99–11.69] |
| † Significant difference between PS and MPS conditions at p<0.05.  ‡ Significant difference between PS and PC conditions at p<0.05.  § Significant difference between MPS and PC conditions at p<0.05.  Abbreviations—CI: confidence interval; HR: heart rate; HRmax: maximal heart rate; EP: extended-play in friendly games during the late pre-season preparation phase; M: mean; MPS: mid of the pre-season preparation phase; RAST: running based anaerobic sprint test; n: sample size; PS: prior to the initiation of the nine weeks of pre-season preparation phase; PC: one week prior to the initiation of the competition phase; SD: standard deviation; CODS: change of direction speed; V̇O₂max: maximal oxygen uptake; VT₂: second ventilatory threshold; vV̇O₂max: velocity at V̇O₂max; vVT₂: velocity at VT₂. | | | |

| **Table A2.** Physical, biochemical, hormonal, haematological, and performance data of the LP cohort (n=12) for PS, MPS, and PC conditions, presented as M±SD[95%CI]. | | | |
| --- | --- | --- | --- |
| **Variable** | **PS** | **MPS** | **PC** |
| Body mass (kg) | 72.24±8.19[67.61–76.87] | 71.93±8.24[67.27–76.59] | 72.80±7.38[68.62–76.98] |
| Body fat (%) | 12.32±3.31[10.45–14.19] | 11.79±2.98[10.10–13.48] | 11.33±2.17[10.10–12.56] |
| †‡ Rate of perceived exertion (Borg scale, 1–10) | 5.33±0.65[4.96–5.70] | 7.50±0.80[7.05–7.95] | 7.50±0.80[7.05–7.95] |
| Glucose (mg·dl⁻¹) | 87.17±13.55[79.50–94.84] | 83.58±9.31[78.31–88.85] | 84.08±6.73[80.27–87.89] |
| § Cholesterol (mg·dl⁻¹) | 159.42±33.12[140.68–178.16] | 154.42±25.44[140.03–168.81] | 169.58±23.28[156.41–182.75] |
| § Τriglycerides (mg·dl⁻¹) | 97.50±33.88[78.33–116.67] | 71.58±20.83[59.79–83.37] | 90.50±23.49[77.21–103.79] |
| Serum glutamic-oxaloacetic transaminase (U·l⁻¹) | 29.17±14.92[20.73–37.61] | 27.33±7.18[23.27–31.39] | 26.50±7.96[22.00–31.00] |
| Serum glutamic-pyruvic transaminase (U·l⁻¹) | 23.17±8.00[18.64–27.70] | 24.25±5.31[21.25–27.25] | 25.67±7.88[21.21–30.13] |
| Creatine phosphokinase (U·l⁻¹) | 238.25±90.88[186.83–289.67] | 309.75±106.98[249.22–370.28] | 275.08±150.93[189.68–360.48] |
| †§ Myoglobin (mg·l⁻¹) | 71.51±31.27[53.82–89.20] | 111.00±32.35[92.7–129.30] | 81.33±31.36[63.59–99.07] |
| † Iron (μg·dl⁻¹) | 117.67±30.47[100.43–134.91] | 92.25±32.31[73.97–110.53] | 104.50±32.58[86.07–122.93] |
| Ferritin (mg·l⁻¹) | 51.43±26.26[36.57–66.29] | 48.64±21.67[36.38–60.90] | 50.56±22.0[38.11–63.01] |
| C-reactive protein (mg·l⁻¹) | 0.72±0.90[0.21–1.23] | 0.31±0.05[0.28–0.34] | 0.32±0.07[0.28–0.36] |
| †§ Hs C-reactive protein (mg·l⁻¹) | 0.77±0.21[0.65–0.89] | 1.80±0.27[1.65–1.95] | 0.80±0.16[0.71–0.89] |
| †§ Interleukin-6 (mg·l⁻¹) | 6.73±2.25[5.46–8.00] | 7.62±2.72[6.08–9.16] | 6.00±1.85[4.95–7.05] |
| †§ Tumour necrosis factor alpha (pg·ml⁻¹) | 6.38±0.96[5.84–6.92] | 8.62±0.71[8.22–9.02] | 6.02±0.74[5.60–6.44] |
| Testosterone (ng·ml⁻¹) | 5.80±0.91[5.29–6.31] | 5.53±1.14[4.88–6.18] | 6.02±1.46[5.19–6.85] |
| Cortisol (μg·dl⁻¹) | 16.66±3.08[14.92–18.40] | 17.81±4.06[15.51–20.11] | 16.42±3.72[14.32–18.52] |
| Testosterone–cortisol ratio (T:C) | 0.36±0.09[0.31–0.41] | 0.32±0.06[0.29–0.35] | 0.38±0.10[0.32–0.44] |
| Red blood cell count (10¹²RBC·l⁻¹) | 5.35±0.62[5.00–5.70] | 5.29±0.65[4.92–5.66] | 5.49±0.74[5.07–5.91] |
| Haemoglobin (g·dl⁻¹) | 15.18±1.51[14.33–16.03] | 14.72±1.02[14.14–15.3] | 14.91±0.82[14.45–15.37] |
| Haematocrit (%) | 45.39±2.83[43.79–46.99] | 44.86±2.24[43.59–46.13] | 45.14±2.98[43.45–46.83] |
| White blood cell count (10⁹WBC·l⁻¹) | 6.39±1.56[5.51–7.27] | 6.40±1.70[5.44–7.36] | 6.34±1.86[5.29–7.39] |
| Platelet count (10⁹PLT·l⁻¹) | 229.67±55.61[198.21–261.13] | 219.67±52.70[189.85–249.49] | 206.25±53.11[176.20–236.30] |
| ‡§ Countermovement jump height (cm) | 39.18±5.43[36.11–42.25] | 39.68±6.24[36.15–43.21] | 41.91±5.19[38.97–44.85] |
| †‡ Power of knee extensors, dominant leg (watt) | 175.08±20.81[163.31–186.85] | 191.83±21.87[179.46–204.20] | 192.50±24.07[178.88–206.12] |
| †‡ Power of knee extensors, non-dominant leg (watt) | 181.75±20.96[169.89–193.61] | 200.00±21.80[187.67–212.33] | 196.33±24.03[182.73–209.93] |
| Power of knee flexors, dominant leg (watt) | 123.00±22.32[110.37–135.63] | 132.25±14.67[123.95–140.55] | 131.75±17.92[121.61–141.89] |
| Power of knee flexors, non-dominant leg (watt) | 110.42±18.37[100.03–120.81] | 124.83±19.95[113.54–136.12] | 125.75±17.82[115.67–135.83] |
| †‡ 10-m sprint time (s) | 1.77±0.07[1.73–1.81] | 1.69±0.06[1.66–1.72] | 1.67±0.07[1.63–1.71] |
| ‡§ CODS (s) | 16.54±0.6[16.20–16.88] | 16.43±0.59[16.10–16.76] | 16.07±0.67[15.69–16.45] |
| † Average 35-m running time during RAST (s) | 5.13±0.17[5.03–5.23] | 5.04±0.17[4.94–5.14] | 5.14±0.31[4.96–5.32] |
| † Speed drop during RAST (%) | 9.29±3.31[7.42–11.16] | 6.06±2.94[4.40–7.72] | 9.39±2.76[7.83–10.95] |
| †‡ Hamstring and lower back flexibility (cm) | 21.17±4.84[18.43–23.91] | 23.08±4.66[20.44–25.72] | 24.33±4.91[21.55–27.11] |
| ‡§ V̇O₂max (ml·kg⁻¹·min⁻¹) | 54.06±4.37[51.59–56.53] | 55.68±4.43[53.17–58.19] | 57.25±3.59[55.22–59.28] |
| † vV̇O₂max (km·hr⁻¹) | 16.00±0.83[15.53–16.47] | 16.54±0.86[16.05–17.03] | 16.29±1.03[15.71–16.87] |
| † vVT₂ (km·hr⁻¹) | 12.13±0.68[11.75–12.51] | 12.67±0.78[12.23–13.11] | 12.35±0.61[12.00–12.70] |
| HRmax at V̇O₂max (b·min⁻¹) | 198.17±7.91[193.69–202.65] | 195.33±8.77[190.37–200.29] | 194.67±9.32[189.40–199.94] |
| HR at VT₂ (b·min⁻¹) | 169.58±9.08[164.44–174.72] | 168.5±8.03[163.96–173.04] | 167.67±10.11[161.95–173.39] |
| ‡ Lactate at V̇O₂max (mmol·l⁻¹) | 11.23±0.65[10.86–11.60] | 11.58±1.53[10.71–12.45] | 12.67±1.06[12.07–13.27] |
| † Significant difference between PS and MPS conditions at p<0.05.  ‡ Significant difference between PS and PC conditions at p<0.05.  § Significant difference between MPS and PC conditions at p<0.05.  Abbreviations—CI: confidence interval; HR: heart rate; HRmax: maximal heart rate; LP: limited-play in friendly games during the late pre-season preparation phase; M: mean; MPS: mid of the pre-season preparation phase; RAST: running based anaerobic sprint test; n: sample size; PS: prior to the initiation of the nine weeks of pre-season preparation phase; PC: one week prior to the initiation of the competition phase; SD: standard deviation; CODS: change of direction speed; V̇O₂max: maximal oxygen uptake; VT₂: second ventilatory threshold; vV̇O₂max: velocity at V̇O₂max; vVT₂: velocity at VT₂. | | | |

| **Table A3.** Bonferroni-adjusted p-values for pairwise comparisons between conditions (PS-MPS, PS-PC, and MPS-PC) across all variables of the EP cohort (n=12). | | | |
| --- | --- | --- | --- |
| **Variable** | **PS-MPS** | **PS-PC** | **MPS-PC** |
| ‡§ Body mass | 0.991 | 0.026 | 0.047 |
| †‡§ Body fat | <0.001 | <0.001 | 0.001 |
| †‡ Rate of perceived exertion | <0.001 | <0.001 | >0.999 |
| Glucose | 0.248 | >0.999 | 0.056 |
| ‡§ Cholesterol | 0.465 | 0.025 | 0.009 |
| § Τriglycerides | 0.489 | >0.999 | 0.039 |
| ‡§ Serum glutamic-oxaloacetic transaminase | 0.090 | 0.006 | 0.023 |
| ‡ Serum glutamic-pyruvic transaminase | 0.173 | 0.019 | 0.176 |
| ‡ Creatine phosphokinase | 0.088 | 0.017 | 0.679 |
| †‡ Myoglobin | <0.001 | 0.011 | 0.927 |
| Iron | >0.999 | >0.999 | >0.999 |
| Ferritin | 0.459 | 0.296 | >0.999 |
| C-reactive protein | 0.082 | 0.427 | 0.831 |
| †‡§ Hs C-reactive protein | <0.001 | <0.001 | <0.001 |
| †‡§ Interleukin-6 | 0.035 | <0.001 | <0.001 |
| †‡§ Tumor necrosis factor alpha | <0.001 | <0.001 | <0.001 |
| ‡§ Testosterone | 0.128 | 0.006 | 0.026 |
| Cortisol | 0.075 | 0.226 | 0.414 |
| § Testosterone–cortisol ratio | 0.768 | 0.131 | 0.014 |
| †§ Red blood cell count | 0.006 | 0.221 | 0.001 |
| †‡ Haemoglobin | 0.008 | 0.017 | 0.509 |
| † Haematocrit | 0.006 | 0.199 | 0.458 |
| † White blood cell count | 0.048 | 0.517 | 0.441 |
| Platelet count | >0.999 | >0.999 | >0.999 |
| ‡§ Countermovement jump height | 0.478 | 0.019 | 0.006 |
| ‡§ Power of knee extensors, dominant leg | 0.130 | 0.010 | 0.019 |
| ‡§ Power of knee extensors, non-dominant leg | 0.409 | 0.010 | 0.002 |
| ‡§ Power of knee flexors, dominant leg | >0.999 | 0.036 | 0.007 |
| ‡§ Power of knee flexors, non-dominant leg | 0.613 | <0.001 | 0.004 |
| †‡ 10-m sprint time | <0.001 | 0.001 | >0.999 |
| †‡§ Agility | 0.002 | <0.001 | <0.001 |
| †‡§ Average 35-m running time during RAST | <0.001 | <0.001 | 0.001 |
| † Speed drop during RAST | 0.019 | >0.999 | 0.232 |
| Hamstring and lower back flexibility | >0.999 | 0.061 | 0.437 |
| †‡§ V̇O₂max | <0.001 | <0.001 | 0.039 |
| †‡§ vV̇O₂max | 0.038 | <0.001 | 0.004 |
| †‡§ vVT₂) | 0.002 | <0.001 | <0.001 |
| † HRmax at V̇O₂max | 0.002 | 0.072 | 0.765 |
| ‡ HR at VT₂ | 0.057 | 0.019 | >0.999 |
| †‡ Lactate at V̇O₂max | 0.040 | 0.048 | >0.999 |
| † Significant difference between PS and MPS conditions at p ≤ 0.05.  ‡ Significant difference between PS and PC conditions at p ≤ 0.05.  § Significant difference between MPS and PC conditions at p ≤ 0.05.  Abbreviations—HR: heart rate; HRmax: maximal heart rate; EP: extended-play in friendly games during the late pre-season preparation phase; MPS: mid of the pre-season preparation phase; n: sample size; RAST: running based anaerobic sprint test; PS: prior to the initiation of the nine weeks of pre-season preparation phase; PC: one week prior to the initiation of the competition phase; V̇O₂max: maximal oxygen uptake; VT₂: second ventilatory threshold; vV̇O₂max: velocity at V̇O₂max; vVT₂: velocity at VT₂. | | | |

| **Table A4.** Bonferroni-adjusted p-values for pairwise comparisons between conditions (PS-MPS, PS-PC, and MPS-PC) across all variables of the LP cohort (n=12). | | | |
| --- | --- | --- | --- |
| **Variable** | **PS-MPS** | **PS-PC** | **MPS-PC** |
| Body mass | >0.999 | >0.999 | 0.268 |
| Body fat | 0.372 | 0.188 | 0.951 |
| †‡ Rate of perceived exertion | <0.001 | <0.001 | >0.999 |
| Glucose | >0.999 | >0.999 | >0.999 |
| § Cholesterol | >0.999 | 0.447 | 0.018 |
| § Τriglycerides | 0.070 | >0.999 | 0.046 |
| Serum glutamic-oxaloacetic transaminase | >0.999 | >0.999 | >0.999 |
| Serum glutamic-pyruvic transaminase | >0.999 | >0.999 | >0.999 |
| Creatine phosphokinase | 0.125 | >0.999 | 0.631 |
| †§ Myoglobin | 0.015 | 0.825 | 0.010 |
| † Iron | 0.042 | 0.305 | 0.165 |
| Ferritin | 0.839 | >0.999 | >0.999 |
| C-reactive protein | 0.418 | 0.466 | >0.999 |
| †§ Hs C-reactive protein | <0.001 | >0.999 | <0.001 |
| †§ Interleukin-6 | 0.036 | 0.446 | 0.049 |
| †§ Tumor necrosis factor alpha | <0.001 | >0.999 | <0.001 |
| Testosterone | 0.987 | >0.999 | >0.999 |
| Cortisol | >0.999 | >0.999 | >0.999 |
| Testosterone–cortisol ratio | >0.999 | >0.999 | 0.074 |
| Red blood cell count | >0.999 | >0.999 | 0.329 |
| Haemoglobin | 0.305 | >0.999 | >0.999 |
| Haematocrit | 0.836 | >0.999 | >0.999 |
| White blood cell count | >0.999 | >0.999 | >0.999 |
| Platelet count | 0.332 | 0.060 | 0.266 |
| ‡§ Countermovement jump height | >0.999 | <0.001 | 0.010 |
| †‡ Power of knee extensors, dominant leg | 0.025 | 0.025 | >0.999 |
| †‡ Power of knee extensors, non-dominant leg | 0.010 | 0.021 | 0.860 |
| Power of knee flexors, dominant leg | 0.708 | 0.488 | >0.999 |
| Power of knee flexors, non-dominant leg | 0.088 | 0.053 | >0.999 |
| †‡ 10-m sprint time | 0.001 | 0.008 | 0.976 |
| ‡§ Agility | 0.578 | 0.003 | 0.018 |
| † Average 35-m running time during RAST | 0.003 | >0.999 | 0.391 |
| † Speed drop during RAST | 0.036 | >0.999 | 0.087 |
| †‡ Hamstring and lower back flexibility | <0.001 | 0.001 | 0.425 |
| ‡§ V̇O₂max | 0.076 | 0.001 | 0.002 |
| † vV̇O₂max | 0.009 | 0.394 | 0.825 |
| † vVT₂ | 0.035 | 0.387 | 0.441 |
| HRmax at V̇O₂max | 0.130 | 0.134 | >0.999 |
| HR at VT₂ | >0.999 | >0.999 | >0.999 |
| ‡ Lactate at V̇O₂max | >0.999 | 0.017 | 0.164 |
| † Significant difference between PS and MPS conditions at p ≤ 0.05.  ‡ Significant difference between PS and PC conditions at p ≤ 0.05.  § Significant difference between MPS and PC conditions at p ≤ 0.05.  Abbreviations—HR: heart rate; HRmax: maximal heart rate; LP: limited-play in friendly games during the late pre-season preparation phase; MPS: mid of the pre-season preparation phase; n: sample size; RAST: running based anaerobic sprint test; PS: prior to the initiation of the nine weeks of pre-season preparation phase; PC: one week prior to the initiation of the competition phase; V̇O₂max: maximal oxygen uptake; VT₂: second ventilatory threshold; vV̇O₂max: velocity at V̇O₂max; vVT₂: velocity at VT₂. | | | |

| **Table A5.** Cohen’s *d* effect sizes and 95% CIs, enclosed in square brackets, for pairwise comparisons between conditions (PS-MPS, PS-PC, and MPS-PC) across all variables of the EP cohort (n=12). | | | |
| --- | --- | --- | --- |
| **Variable** | **PS-MPS** | **PS-PC** | **MPS-PC** |
| ‡§ Body mass | -0.11 [-0.68 – 0.46] | -0.36 [-0.95 – 0.23] | -0.25 [-0.82 – 0.32] |
| †‡§ Body fat | -1.02 [-1.73 – -0.31] | -1.76 [-2.66 – -0.86] | -0.75 [-1.40 – -0.10] |
| †‡ Rate of perceived exertion | 3.21 [1.80 – 4.62] | 3.31 [1.88 – 4.74] | 0.15 [-0.42 – 0.72] |
| Glucose | -0.91 [-1.58 – -0.24] | -0.21 [-0.78 – 0.36] | 0.95 [0.26 – 1.64] |
| ‡§ Cholesterol | 0.22 [-0.35 – 0.79] | 0.58 [-0.03 – 1.19] | 0.36 [-0.23 – 0.95] |
| § Τriglycerides | -0.41 [-1.00 – 0.18] | -0.02 [-0.59 – 0.55] | 0.59 [-0.02 – 1.20] |
| ‡§ Serum glutamic-oxaloacetic transaminase | 0.70 [0.07 – 1.33] | 1.20 [0.46 – 1.94] | 0.68 [0.05 – 1.31] |
| ‡ Serum glutamic-pyruvic transaminase | 0.62 [-0.01 – 1.25] | 0.88 [0.21 – 1.55] | 0.37 [-0.22 – 0.96] |
| ‡ Creatine phosphokinase | 1.02 [0.31 – 1.73] | 1.23 [0.49 – 1.97] | 0.30 [-0.29 – 0.89] |
| †‡ Myoglobin | 1.27 [0.51 – 2.03] | 1.02 [0.31 – 1.73] | 0.25 [-0.32 – 0.82] |
| Iron | -0.24 [-0.81 – 0.33] | -0.08 [-0.65 – 0.49] | 0.42 [-0.17 – 1.01] |
| Ferritin | -0.35 [-0.94 – 0.24] | -0.46 [-1.05 – 0.13] | -0.17 [-0.74 – 0.40] |
| C-reactive protein | 0.90 [0.23 – 1.57] | 0.60 [-0.01 – 1.21] | -0.27 [-0.84 – 0.30] |
| †‡§ Hs C-reactive protein | 2.92 [1.63 – 4.21] | 5.35 [3.14 – 7.56] | 2.36 [1.26 – 3.46] |
| †‡§ Interleukin-6 | 0.60 [-0.01 – 1.21] | 3.17 [1.78 – 4.56] | 2.39 [1.27 – 3.51] |
| †‡§ Tumor necrosis factor alpha | 2.20 [1.16 – 3.24] | 8.76 [5.21 – 12.31] | 3.56 [2.03 – 5.09] |
| ‡§ Testosterone | 0.58 [-0.03 – 1.19] | 1.30 [0.54 – 2.06] | 0.81 [0.16 – 1.46] |
| Cortisol | 0.94 [0.25 – 1.63] | 0.46 [-0.13 – 1.05] | -0.57 [-1.18 – 0.04] |
| § Testosterone–cortisol ratio | -0.64 [-1.27 – -0.01] | 0.42 [-0.17 – 1.01] | 1.20 [0.46 – 1.94] |
| †§ Red blood cell count | -0.56 [-1.17 – 0.05] | 0.47 [-0.12 – 1.06] | 1.11 [0.38 – 1.84] |
| †‡ Haemoglobin | -0.83 [-1.48 – -0.18] | -0.58 [-1.19 – 0.03] | 0.25 [-0.32 – 0.82] |
| † Haematocrit | -0.76 [-1.41 – -0.11] | -0.47 [-1.06 – 0.12] | 0.29 [-0.28 – 0.86] |
| † White blood cell count | -0.68 [-1.31 – -0.05] | -0.40 [-0.99 – 0.19] | 0.30 [-0.29 – 0.89] |
| Platelet count | -0.33 [-0.92 – 0.26] | -0.20 [-0.77 – 0.37] | 0.19 [-0.38 – 0.76] |
| ‡§ Countermovement jump height | 0.40 [-0.19 – 0.99] | 0.97 [0.28 – 1.66] | 0.61 [0.00 – 1.22] |
| ‡§ Power of knee extensors, dominant leg | 0.61 [0.00 – 1.22] | 1.33 [0.55 – 2.11] | 0.64 [0.01 – 1.27] |
| ‡§ Power of knee extensors, non-dominant leg | 0.36 [-0.23 – 0.95] | 1.10 [0.37 – 1.83] | 0.73 [0.10 – 1.36] |
| ‡§ Power of knee flexors, dominant leg | 0.09 [-0.48 – 0.66] | 0.81 [0.16 – 1.46] | 1.47 [0.65 – 2.29] |
| ‡§ Power of knee flexors, non-dominant leg | 0.18 [-0.39 – 0.75] | 0.66 [0.03 – 1.29] | 0.49 [-0.12 – 1.10] |
| †‡ 10-m sprint time | -2.20 [-3.24 – -1.16] | -2.75 [-3.98 – -1.52] | 0.00 [-0.57 – 0.57] |
| †‡§ Agility | -0.81 [-1.46 – -0.16] | -2.26 [-3.32 – -1.20] | -1.58 [-2.42 – -0.74] |
| †‡§ Average 35-m running time during RAST | -0.71 [-1.34 – -0.08] | -2.13 [-3.15 – -1.11] | -1.60 [-2.46 – -0.74] |
| † Speed drop during RAST | -1.47 [-2.29 – -0.65] | -0.17 [-0.74 – 0.40] | 0.67 [0.04 – 1.3] |
| Hamstring and lower back flexibility | 0.13 [-0.44 – 0.70] | 0.42 [-0.17 – 1.01] | 0.26 [-0.31 – 0.83] |
| †‡§ V̇O₂max | 0.85 [0.18 – 1.52] | 1.34 [0.56 – 2.12] | 0.42 [-0.17 – 1.01] |
| †‡§ vV̇O₂max | 0.77 [0.12 – 1.42] | 1.84 [0.92 – 2.76] | 1.09 [0.38 – 1.80] |
| †‡§ vVT₂) | 1.03 [0.32 – 1.74] | 2.43 [1.31 – 3.55] | 1.56 [0.72 – 2.40] |
| † HRmax at V̇O₂max | -0.69 [-1.32 – -0.06] | -0.44 [-1.03 – 0.15] | 0.16 [-0.41 – 0.73] |
| ‡ HR at VT₂ | -0.61 [-1.22 – 0.00] | -0.76 [-1.41 – -0.11] | -0.10 [-0.67 – 0.47] |
| †‡ Lactate at V̇O₂max | -1.21 [-1.95 – -0.47] | -1.17 [-1.90 – -0.44] | 0.05 [-0.52 – 0.62] |
| † Significant difference between PS and MPS conditions at p ≤ 0.05.  ‡ Significant difference between PS and PC conditions at p ≤ 0.05.  § Significant difference between MPS and PC conditions at p ≤ 0.05.  Abbreviations—CI: confidence interval; HR: heart rate; HRmax: maximal heart rate; EP: extended-play in friendly games during the late pre-season preparation phase; MPS: mid of the pre-season preparation phase; n: sample size; RAST: running based anaerobic sprint test; PS: prior to the initiation of the nine weeks of pre-season preparation phase; PC: one week prior to the initiation of the competition phase; V̇O₂max: maximal oxygen uptake; VT₂: second ventilatory threshold; vV̇O₂max: velocity at V̇O₂max; vVT₂: velocity at VT₂. | | | |

| **Table A6.** Cohen’s *d* effect sizes and 95% CIs, enclosed in square brackets, for pairwise comparisons between conditions (PS-MPS, PS-PC, and MPS-PC) across all variables of the LP cohort (n=12). | | | |
| --- | --- | --- | --- |
| **Variable** | **PS-MPS** | **PS-PC** | **MPS-PC** |
| Body mass | -0.04 [-0.61 – 0.53] | 0.07 [-0.50 – 0.64] | 0.11 [-0.46 – 0.68] |
| Body fat | -0.17 [-0.74 – 0.40] | -0.34 [-0.93 – 0.25] | -0.17 [-0.74 – 0.40] |
| †‡ Rate of perceived exertion | 2.96 [1.65 – 4.27] | 2.96 [1.65 – 4.27] | 0.00 [-0.57 – 0.57] |
| Glucose | -0.3 [-0.89 – 0.29] | -0.26 [-0.83 – 0.31] | 0.06 [-0.51 – 0.63] |
| § Cholesterol | -0.17 [-0.74 – 0.40] | 0.34 [-0.25 – 0.93] | 0.62 [-0.01 – 1.25] |
| § Τriglycerides | -0.88 [-1.55 – -0.21] | -0.23 [-0.8 – 0.34] | 0.85 [0.18 – 1.52] |
| Serum glutamic-oxaloacetic transaminase | -0.14 [-0.71 – 0.43] | -0.21 [-0.78 – 0.36] | -0.11 [-0.68 – 0.46] |
| Serum glutamic-pyruvic transaminase | 0.15 [-0.42 – 0.72] | 0.31 [-0.28 – 0.90] | 0.20 [-0.37 – 0.77] |
| Creatine phosphokinase | 0.72 [0.09 – 1.35] | 0.28 [-0.29 – 0.85] | -0.26 [-0.83 – 0.31] |
| †§ Myoglobin | 1.24 [0.50 – 1.98] | 0.31 [-0.28 – 0.90] | -0.93 [-1.62 – -0.24] |
| † Iron | -0.81 [-1.46 – -0.16] | -0.42 [-1.01 – 0.17] | 0.38 [-0.21 – 0.97] |
| Ferritin | -0.11 [-0.68 – 0.46] | -0.04 [-0.61 – 0.53] | 0.09 [-0.48 – 0.66] |
| C-reactive protein | -0.47 [-1.06 – 0.12] | -0.46 [-1.05 – 0.13] | 0.17 [-0.40 – 0.74] |
| †§ Hs C-reactive protein | 4.12 [2.38 – 5.86] | 0.16 [-0.41 – 0.73] | -4.17 [-5.93 – -2.41] |
| †§ Interleukin-6 | 0.35 [-0.24 – 0.94] | -0.35 [-0.94 – 0.24] | -0.67 [-1.30 – -0.04] |
| †§ Tumor necrosis factor alpha | 2.60 [1.42 – 3.78] | -0.41 [-1.00 – 0.18] | -3.56 [-5.09 – -2.03] |
| Testosterone | -0.26 [-0.83 – 0.31] | 0.17 [-0.40 – 0.74] | 0.37 [-0.22 – 0.96] |
| Cortisol | 0.31 [-0.28 – 0.90] | -0.07 [-0.64 – 0.50] | -0.36 [-0.95 – 0.23] |
| Testosterone–cortisol ratio | -0.50 [-1.11 – 0.11] | 0.20 [-0.37 – 0.77] | 0.67 [0.04 – 1.30] |
| Red blood cell count | -0.09 [-0.66 – 0.48] | 0.20 [-0.37 – 0.77] | 0.29 [-0.28 – 0.86] |
| Haemoglobin | -0.35 [-0.94 – 0.24] | -0.21 [-0.78 – 0.36] | 0.20 [-0.37 – 0.77] |
| Haematocrit | -0.20 [-0.77 – 0.37] | -0.09 [-0.66 – 0.48] | 0.10 [-0.47 – 0.67] |
| White blood cell count | 0.01 [-0.56 – 0.58] | -0.03 [-0.60 – 0.54] | -0.03 [-0.60 – 0.54] |
| Platelet count | -0.18 [-0.75 – 0.39] | -0.43 [-1.02 – 0.16] | -0.25 [-0.82 – 0.32] |
| ‡§ Countermovement jump height | 0.09 [-0.48 – 0.66] | 0.51 [-0.10 – 1.12] | 0.39 [-0.20 – 0.98] |
| †‡ Power of knee extensors, dominant leg | 0.78 [0.13 – 1.43] | 0.77 [0.12 – 1.42] | 0.03 [-0.54 – 0.60] |
| †‡ Power of knee extensors, non-dominant leg | 0.85 [0.18 – 1.52] | 0.64 [0.01 – 1.27] | -0.16 [-0.73 – 0.41] |
| Power of knee flexors, dominant leg | 0.47 [-0.12 – 1.06] | 0.43 [-0.16 – 1.02] | -0.03 [-0.60 – 0.54] |
| Power of knee flexors, non-dominant leg | 0.75 [0.10 – 1.40] | 0.85 [0.18 – 1.52] | 0.05 [-0.52 – 0.62] |
| †‡ 10-m sprint time | -1.14 [-1.87 – -0.41] | -1.43 [-2.23 – -0.63] | -0.29 [-0.86 – 0.28] |
| ‡§ Agility | -0.18 [-0.75 – 0.39] | -0.73 [-1.36 – -0.10] | -0.57 [-1.18 – 0.04] |
| † Average 35-m running time during RAST | -0.53 [-1.14 – 0.08] | 0.04 [-0.53 – 0.61] | 0.37 [-0.22 – 0.96] |
| † Speed drop during RAST | -1.03 [-1.74 – -0.32] | 0.03 [-0.54 – 0.60] | 1.17 [0.44 – 1.90] |
| †‡ Hamstring and lower back flexibility | 0.40 [-0.19 – 0.99] | 0.65 [0.02 – 1.28] | 0.26 [-0.31 – 0.83] |
| ‡§ V̇O₂max | 0.37 [-0.22 – 0.96] | 0.79 [0.14 – 1.44] | 0.38 [-0.21 – 0.97] |
| † vV̇O₂max | 0.64 [0.01 – 1.27] | 0.31 [-0.28 – 0.90] | -0.26 [-0.83 – 0.31] |
| † vVT₂ | 0.73 [0.10 – 1.36] | 0.34 [-0.25 – 0.93] | -0.45 [-1.04 – 0.14] |
| HRmax at V̇O₂max | -0.34 [-0.93 – 0.25] | -0.40 [-0.99 – 0.19] | -0.07 [-0.64 – 0.50] |
| HR at VT₂ | -0.13 [-0.70 – 0.44] | -0.20 [-0.77 – 0.37] | -0.09 [-0.66 – 0.48] |
| ‡ Lactate at V̇O₂max | 0.26 [-0.31 – 0.83] | 1.55 [0.71 – 2.39] | 0.80 [0.15 – 1.45] |
| † Significant difference between PS and MPS conditions at p ≤ 0.05.  ‡ Significant difference between PS and PC conditions at p ≤ 0.05.  § Significant difference between MPS and PC conditions at p ≤ 0.05.  Abbreviations—CI: confidence interval; HR: heart rate; HRmax: maximal heart rate; LP: limited-play in friendly games during the late pre-season preparation phase; MPS: mid of the pre-season preparation phase; n: sample size; RAST: running based anaerobic sprint test; PS: prior to the initiation of the nine weeks of pre-season preparation phase; PC: one week prior to the initiation of the competition phase; V̇O₂max: maximal oxygen uptake; VT₂: second ventilatory threshold; vV̇O₂max: velocity at V̇O₂max; vVT₂: velocity at VT₂. | | | |

| **Table A7.** Pearson's correlation coefficients (r) from pairwise comparisons between total time played in friendly games and physical, biochemical, hormonal, haematological, and performance variables that were observed one week prior to the initiation of the competition phase in EP and LP cohorts and across all participants. | | | |
| --- | --- | --- | --- |
| **Variable** | **EP  (n=12)** | **LP  (n=12)** | **Participants overall  (total n=24)** |
| Body mass | 0.36 | 0.44 | 0.32 |
| Body fat | -0.13 | -0.13 | -0.47* |
| Rate of perceived exertion | 0.54 | 0.39 | -0.02 |
| Glucose | 0.02 | 0.08 | 0.36 |
| Cholesterol | 0.13 | 0.15 | 0.09 |
| Τriglycerides | -0.14 | -0.09 | -0.14 |
| Serum glutamic-oxaloacetic transaminase | 0.15 | 0.04 | 0.22 |
| Serum glutamic-pyruvic transaminase | 0.04 | 0.30 | 0.12 |
| Creatine phosphokinase | 0.44 | -0.11 | -0.10 |
| Myoglobin | 0.46 | -0.25 | 0.13 |
| Iron | -0.09 | -0.14 | -0.44* |
| Ferritin | -0.11 | 0.42 | 0.44* |
| C-reactive protein | 0.15 | 0.32 | 0.28 |
| Hs C-reactive protein | 0.38 | -0.29 | 0.86* |
| Interleukin-6 | -0.35 | 0.17 | 0.79* |
| Tumor necrosis factor alpha | -0.43 | -0.03 | 0.82* |
| Testosterone | 0.03 | 0.09 | 0.21 |
| Cortisol | -0.15 | 0.14 | -0.03 |
| Testosterone–cortisol ratio | 0.10 | -0.08 | 0.21 |
| Red blood cell count | 0.03 | 0.20 | 0.15 |
| Haemoglobin | 0.34 | -0.33 | -0.03 |
| Haematocrit | 0.36 | -0.16 | -0.02 |
| White blood cell count | 0.19 | 0.23 | 0.13 |
| Platelet count | 0.19 | 0.25 | 0.22 |
| Countermovement jump height | 0.23 | 0.14 | 0.10 |
| Power of knee extensors, dominant leg | -0.09 | 0.33 | 0.55* |
| Power of knee extensors, non-dominant leg | -0.24 | 0.14 | 0.44* |
| Power of knee flexors, dominant leg | -0.15 | 0.39 | 0.67* |
| Power of knee flexors, non-dominant leg | -0.16 | 0.13 | 0.49* |
| 10-m sprint time | -0.38 | 0.39 | 0.38 |
| Agility | -0.22 | -0.22 | -0.37 |
| Average 35-m running time during RAST | -0.13 | -0.13 | -0.52* |
| Speed drop rate during RAST | 0.49 | 0.48 | 0.24 |
| Hamstring and lower back flexibility | 0.12 | -0.11 | 0.16 |
| V̇O₂max | -0.06 | -0.42 | -0.09 |
| vV̇O₂max | -0.26 | -0.38 | 0.38 |
| vVT₂ | -0.40 | -0.48 | 0.55* |
| HRmax at V̇O₂max | -0.41 | 0.14 | -0.27 |
| HR at VT₂ | -0.41 | 0.13 | -0.32 |
| Lactate at V̇O₂max | -0.42 | 0.03 | -0.62* |
| * Significant correlation at p<0.05.  Abbreviations—HR: heart rate; HRmax: maximal heart rate; EP: extended-play; n: sample size; RAST: running based anaerobic sprint test; V̇O₂max: maximal oxygen uptake; VT₂: second ventilatory threshold; vV̇O₂max: velocity at V̇O₂max; vVT₂: velocity at VT₂. | | | |
